# eHealth in Geriatric Rehabilitation: Systematic Review of Effectiveness, Feasibility, and Usability

**DOI:** 10.2196/24015

**Published:** 2021-08-19

**Authors:** Jules J M Kraaijkamp, Eléonore F van Dam van Isselt, Anke Persoon, Anke Versluis, Niels H Chavannes, Wilco P Achterberg

**Affiliations:** 1 Department of Public Health and Primary Care Leiden University Medical Center Leiden Netherlands; 2 ZZG Zorggroep Nijmegen Netherlands; 3 Department of Primary and Community Care Radboud University Medical Center Nijmegen Nijmegen Netherlands

**Keywords:** geriatric rehabilitation, eHealth, mHealth, digital health, effectiveness, feasibility, usability, systematic review

## Abstract

**Background:**

eHealth has the potential to improve outcomes such as physical activity or balance in older adults receiving geriatric rehabilitation. However, several challenges such as scarce evidence on effectiveness, feasibility, and usability hinder the successful implementation of eHealth in geriatric rehabilitation.

**Objective:**

The aim of this systematic review was to assess evidence on the effectiveness, feasibility, and usability of eHealth interventions in older adults in geriatric rehabilitation.

**Methods:**

We searched 7 databases for randomized controlled trials, nonrandomized studies, quantitative descriptive studies, qualitative research, and mixed methods studies that applied eHealth interventions during geriatric rehabilitation. Included studies investigated a combination of effectiveness, usability, and feasibility of eHealth in older patients who received geriatric rehabilitation, with a mean age of ≥70 years. Quality was assessed using the Mixed Methods Appraisal Tool and a narrative synthesis was conducted using a harvest plot.

**Results:**

In total, 40 studies were selected, with clinical heterogeneity across studies. Of 40 studies, 15 studies (38%) found eHealth was at least as effective as non-eHealth interventions (56% of the 27 studies with a control group), 11 studies (41%) found eHealth interventions were more effective than non-eHealth interventions, and 1 study (4%) reported beneficial outcomes in favor of the non-eHealth interventions. Of 17 studies, 16 (94%) concluded that eHealth was feasible. However, high exclusion rates were reported in 7 studies of 40 (18%). Of 40 studies, 4 (10%) included outcomes related to usability and indicated that there were certain aging-related barriers to cognitive ability, physical ability, or perception, which led to difficulties in using eHealth.

**Conclusions:**

eHealth can potentially improve rehabilitation outcomes for older patients receiving geriatric rehabilitation. Simple eHealth interventions were more likely to be feasible for older patients receiving geriatric rehabilitation, especially, in combination with another non-eHealth intervention. However, a lack of evidence on usability might hamper the implementation of eHealth. eHealth applications in geriatric rehabilitation show promise, but more research is required, including research with a focus on usability and participation.

## Introduction

The world’s population is aging rapidly. Currently, 143 million people are aged 80 years or older, and this number is expected to rise to around 426 million in 2050 [1]. Although many older adults are relatively fit, functional decline, multimorbidity, and geriatric syndromes such as frailty or falls are common in older adults [2,3]. A combination of these age-associated conditions triggers an increased risk of adverse outcomes such as hospitalization, functional impairments, and even mortality [4]. Postacute care such as geriatric rehabilitation aims to diminish these age-associated risks. Evidence shows that geriatric rehabilitation can improve functional outcomes and reduce nursing home admissions and mortality [5,6]. On the other hand, the rapidly aging populations and lack of staff are putting pressure on the quality, accessibility, and affordability of geriatric rehabilitation. In regard to these problems, the use of eHealth can be seen as important and promising, as it has the potential to simultaneously improve both rehabilitation outcomes and efficiency.

eHealth can be defined as “the use of digital information and communication to support and/or improve health and health care” [7]. Some examples of eHealth are video communication, exergames (ie, active video games), and mobile apps. Although eHealth can be seen as important and promising, successful implementation of eHealth interventions in geriatric rehabilitation is complex, can be time consuming, and involves a variety of determinants on multiple levels [8-10]. To safely and successfully implement eHealth in geriatric rehabilitation, scientific evaluation of eHealth is key [11,12]. Three important outcome measures for the evaluation of eHealth in geriatric rehabilitation can be identified: effectiveness, feasibility, and usability [9,13].

In terms of effectiveness, previous reviews show that eHealth can improve physical activity, gait, and balance in community-dwelling older adults [14-17]. However, the evidence on effective eHealth in geriatric rehabilitation is scarce and fragmented. To our knowledge, no prior reviews have examined the effectiveness of eHealth in geriatric rehabilitation.

To better understand how eHealth can be used safely, feasibility testing is an important first step [18,19]. The aim of feasibility testing is to “determine whether an intervention is appropriate for further testing” [20,21], but a general accepted standard on feasibility testing is lacking. Examples of factors that can be addressed in feasibility testing are adverse events, adherence, and acceptability [10].

Additionally, usable eHealth is also an important prerequisite for successful implementation [13,19,22]. Usability can be defined as “the extent to which a system, product, or service can be used by specified users to achieve specified goals with effectiveness, efficiency, and satisfaction in a specified context of use” [23]. For older adults receiving geriatric rehabilitation, usability is especially crucial, since there are certain age-related barriers that may hamper the usability of eHealth [24-26]. These barriers can be categorized into 4 patient-related domains: cognition, physical ability, perception, and motivation [27]. For example, poor vision can make it harder to distinguish certain icons on screens, or cognitive impairment might lead to problems understanding certain eHealth interventions. Often, eHealth is insufficiently tailored to these age-related barriers [28].

Therefore, a systematic review of eHealth in geriatric rehabilitation including the concepts feasibility, usability, and effectiveness was needed. This systematic review can help speed up the implementation process of eHealth and ensure successful adoption of eHealth overall. The aim of this review was to assess evidence on the effectiveness, feasibility, and usability of eHealth interventions in older adults in geriatric rehabilitation.

## Methods

### Study Registration and Protocol

This systematic review is registered at PROSPERO, with registration number CRD42019133192 [29]. This systematic review was based on the PRISMA (Preferred Reporting Items for Systematic Reviews and Meta-analyses) statement, which is an evidence-based minimum set of items used for reporting in systematic reviews and meta-analyses [30]. The complete checklist for this review can be found in Multimedia Appendix 1.

### Types of Studies and Participants

In this review, we included randomized controlled trials, nonrandomized studies, quantitative descriptive studies, qualitative research, and mixed methods studies. We excluded systematic reviews, abstracts, editorials, and non-English and nonpeer-reviewed studies. Studies were included that examined older patients with a mean age of ≥70 years who received geriatric rehabilitation, which is in line with consensus statements on the organization and delivery of geriatric rehabilitation across Europe [31]. Because there is variability between countries’ health care systems and consequently also between countries’ provisions of geriatric rehabilitation [31,32], we included studies in different types of settings such as (geriatric) rehabilitation centers, skilled nursing facilities, hospitals, or ambulatory settings. Studies that included patients with a chronic disease with no acute functional decline were excluded.

### Interventions and Outcomes

Studies investigated eHealth interventions applied during postacute geriatric rehabilitation. Outcome measures related to the effectiveness of interventions were included if they could be classified based on the World Health Organization’s International Classification of Functioning, Disability, and Health (ICF) model [33], which covers the following domains: body functions and structure, activities, participation, environmental factors, and personal factors. For the purpose of this review, we chose to specify feasibility within the following domains: adverse events, adherence, and exclusion rates. Usability outcome measures were classified based on the MOLD-US framework, which is an evidence-based framework of aging barriers that influence the usability of eHealth in older adults and includes 4 categories: cognition, motivation, physical ability, and perception [27]. We included both primary and secondary outcome measures.

### Sources and Search Strategy

On March 9, 2019, March 10, 2019, and January 11, 2021, we searched the following databases: MEDLINE, PsycINFO, EMBASE, EMCARE, Cochrane Library, Web of Science, and Central databases. For this review, 3 separate search strings were compiled. The first focused on the effectiveness, the second focused on the feasibility, and the third focused on the usability of eHealth interventions in geriatric rehabilitation. The search string focusing on effectiveness included keywords related to older adults, rehabilitation, and eHealth interventions. Studies were identified when at least 2 of 3 keywords were present. The search strings focusing on feasibility and usability included an additional keyword related to feasibility or usability. In both search strings, keywords were combined using MeSH terms using the Boolean operations “or” and “and.” The complete search strings can be found in Multimedia Appendix 2.

### Selection of Studies and Data Extraction

We first screened titles of the identified studies. The abstracts of all potentially relevant studies were then screened by 2 authors independently. Next, full texts were obtained and reviewed by the same authors. We excluded studies that did not meet the inclusion criteria. Disagreements between the 2 authors were discussed until a consensus was reached. If a disagreement could not be resolved, a third reviewer was consulted. Data extraction was performed using Covidence, which is an online systematic review management tool [34]. In Covidence, a data extraction form was constructed that included details of publication (ie, author, year, title, country of study, and funding), study design, methods (ie, inclusion and exclusion criteria, population, randomization, statistical analysis, and outcome measures), sample characteristics (ie, age, number of participants, gender, and diagnosis), eHealth intervention (ie, name of intervention, goal of intervention, delivery of intervention, and application of intervention), and primary and secondary outcomes. As the complexity of eHealth interventions influences implementation, we sorted eHealth interventions ranging from simple (ie, video communications, health sensors, or gateways) to complex (ie, robotics, exergames, or virtual reality) [9,35]. One author then extracted the data. A subset of the data (10% of included studies) was also extracted by a second author to check interrater reliability.

### Quality Appraisal

The quality of included studies was assessed using the Mixed Methods Appraisal Tool (MMAT) [36], which allowed quality assessment across different study designs. The MMAT is a critical appraisal tool specifically designed to assess the quality of 5 types of study designs: qualitative research, randomized controlled trials, nonrandomized studies, quantitative descriptive studies, and mixed methods studies. For each study design, the MMAT provides 5 quality criteria that must be rated with “Yes,” “No,” or “Can’t tell.” Since the calculation of an overall score from the ratings of each criterion is discouraged [36,37], we reported a separate score for each rating. Nevertheless, an overall score was reported, because it provides a general picture of study quality. Studies were not excluded based on study quality [36]. For the randomized controlled trials and nonrandomized designs, we rated the criterion “Are there complete outcome data?” as “No” when the drop-out rate was over 20% [38]. In nonrandomized designs, we rated the criterion “Are the confounders accounted for in the design and analysis?” as “No” when there was no description of additional therapy offered during the study, functional status, or cognitive status. Quality assessment was carried out by one author, and 10% of the included studies were selected at random and additionally assessed by a second author to check interrater reliability.

### Data Analysis and Data Synthesis

In studies that reported outcomes related to effectiveness and included a control group, a narrative synthesis was conducted using a harvest plot [39]. In the harvest plot, primary and secondary outcomes were described and color coded based on ICF domain. For each study, the bars in the harvest plot indicated the total results for the different ICF domains, and the height of the bars represented the methodical quality based on the MMAT. When a study reported multiple consistent results within the same ICF domain, the results were combined in 1 bar. If a study reported conflicting results within the same ICF domain, both results were presented. Randomized controlled trails were represented by a thick contour around bars. A meta-analysis was not feasible since the included studies were too heterogeneous with regard to population, intervention, and outcome measures.

## Results

### Study Selection

**Figure 1:**
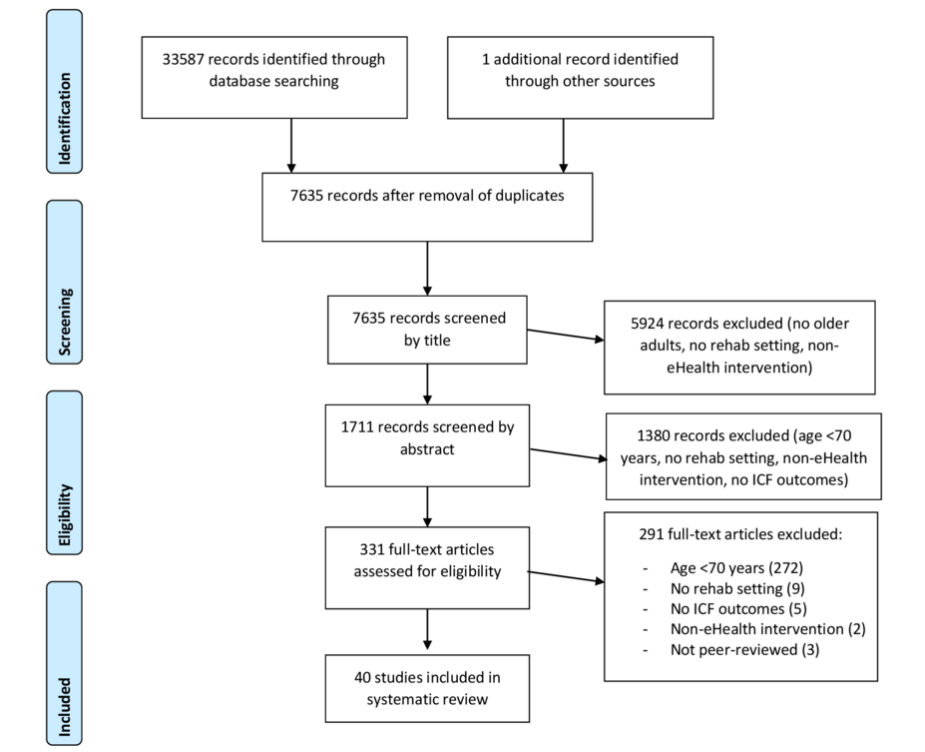
PRISMA flow diagram of search strategy results. ICF: International Classification of Functioning, Disability, and Health.

The search strategy identified a total of 7635 unique records. After exclusion of records based on title and abstract, 331 records remained. During full-text screening, a further 291 records were excluded, resulting in the inclusion of 40 studies in this review. Reasons for exclusion are presented in the study flowchart shown in Figure 1. In 12 cases, a third reviewer was needed to achieve consensus during the process of study selection.

### Study Characteristics

Study characteristics are shown in Table 1. Of the 40 included studies, 18 (45%) were randomized controlled trails [40-57], 2 (5%) had a mixed methods design [58,59], 1 was a qualitative study [60], and 19 (48%) had a quantitative nonrandomized design [61-79], of which 9 studies (of 19, 47%) included a control group [53,61-68,79]. Of 40 studies, 17 studies (43%) were conducted in a hospital setting [41-44,46,50,51,55-57,62,64-66,68,71,79]. Of the 17 hospital-setting studies, 12 (71%) were conducted in a dedicated hospital-rehabilitation unit [41-44,46,50,51,55,56,64,71,79], 2 (12%) were in a hospital-stroke unit [57,68], and 1 (6%) was conducted in a geriatric day hospital [62]. Of the 40 studies, 10 (25%) were conducted in an ambulatory setting [47,48,52-54,60,69,75,76,78], 9 studies (23%) took place in a geriatric rehabilitation setting [40,45,49,58,59,61,63,70,74], 2 studies (5%) were at a tertiary rehabilitation center [60,73], 1 study (3%) was at a skilled nursing facility [77], and 2 studies (5%) did not report the setting [67,72].

**Table 1:** Study characteristics.

Author, year, country	Design	Diagnosis; n; setting	Age (SD); female (%)	Intervention	Use of intervention	Primary outcome domain (primary outcome measure)	Secondary outcome domain(s)
Barnason [53], 2009, United States	RCT^a^	Cardiac; n=55; Ambulatory	71.6 (5.1); 16	Video communication in combination with non-eHealth vs usual care	Daily use, subjects responded to assessment queries, were provided with strategies	Effectiveness, activities (other)	Effectiveness, participation
Backman [59], 2020, United Kingdom	Mixed methods	Orthopedic; n=30; Geriatric rehabilitation	81 (67-96); 63	Mobile apps	Providing access to discharge records during transition to home	Usability	—^b^
Bernocchi [52], 2018, Italy	RCT	Multiple diagnoses; n=146; Ambulatory	79 (6.5); 84	Video communication in combination with non-eHealth vs usual care	Weekly calls; video communication 2×/month; fall prevention program provided by therapist	Effectiveness, activities (other)	Feasibility, effectiveness, activities, participation
Bernocchi [69], 2016, Italy	Quantitative; nonrandomized	Stroke; n=15; Ambulatory	71 (11); 47	Video communication in combination with health sensors	Weekly calls with nurse; weekly video communication with physiotherapist	Feasibility (n completed, n sessions)	Effectiveness, body functions, activities
Cannell [44], 2017, Australia	RCT	Stroke; n=40; Hospital, rehabilitation unit	74 (10); 37.5	Exergames in combination with virtual reality vs usual care	1 hour/session, 5 days/week, in addition to conventional therapy	Effectiveness, activities (maintaining body position)	Effectiveness, activities
Chan [62], 2012, China	Quantitative nonrandomized	Multiple diagnoses; n=90; Geriatric Day hospital	80 (7.1); 73	Exergames vs usual care	10 min/session, 8 sessions total, in addition to conventional therapy	Feasibility (total time spent, average BS^c^ and %MHR^d^)	Effectiveness, activities
Cimarolli [77], 2017, United States	Quantitative; nonrandomized	Multiple diagnoses; n=237; Skilled nurse facility	76 (10.7); 59	Exergames	Recommended use: 2 sessions/week for 15 min, in addition to conventional therapy	Feasibility (time spent, predictors of intense use)	Effectiveness, external factors
Dakin [61], 2011, Australia	Quantitative; nonrandomized	Multiple diagnoses; n=34; Geriatric rehabilitation	77; 47	Health sensors vs usual care	Wore health sensor daily during admission	Effectiveness activities (ADL^e^)	Effectiveness, external factors
Da-Silva [57], 2019, United Kingdom	RCT	Stroke; n=33; Hospital, stroke unit	71; 60.6	Health sensors with reminders vs health sensors without reminders	Wore health sensor for 4 weeks, health sensor vibrated to remind patients to use affected arm	Effectiveness, activities (hand and arm use)	Feasibility, adherence
Doornebosch [70], 2007, Netherlands	Quantitative; nonrandomized	Stroke; n=10; Geriatric rehabilitation	72 (53-94); 80	Robotics	20 minutes/session, 8 sessions total, in addition to conventional therapy	Personal factors (patient’s experience)	Effectiveness, body functions
Edmans [68], 2009, United Kingdom	Quantitative; nonrandomized	Stroke; n=13; Hospital, stroke unit	73; 23	Virtual reality vs usual care	1 hour/session, 5 days/week	Effectiveness, activities (other)	Effectiveness, activities
Franceschini [56], 2020, Italy	RCT	Stroke; n=48; Hospital, rehabilitation unit	72 (64.3); 45.8	Robotics vs usual care	30 minutes/session, 5 days/week over 6 weeks, in addition to conventional therapy	Effectiveness, body functions (muscle power, tone, and reflexes)	Effectiveness, (muscle power, tone, and reflexes)
Gandolfi [71], 2017, Italy	Quantitative; nonrandomized	Stroke; n=2; Hospital, rehabilitation unit	74; 100	Robotics	20 minutes/session, 5 days/week, 10 sessions total, in addition to conventional therapy	Feasibility (compliance, time to set device)	Effectiveness, body functions
Goto [65], 2017, Japan	Quantitative; nonrandomized	Orthopedic; n=20; Hospital	74 (7.5); 90	Robotics vs usual care	Every other day, in addition to conventional therapy	Effectiveness, body functions (mobility of joints)	Effectiveness, body functions
Hesse [42], 2014, Germany	RCT	Stroke; n=50; Hospital, rehabilitation unit	70 (16); 44	Robotics vs usual care	30 minutes/session, 4 days/week, in addition to conventional therapy	Effectiveness, body functions (muscle power, tone, and reflexes)	Effectiveness, body functions, activities, external factors
Hesse [72], 2010, Germany	Quantitative; nonrandomized	Stroke; n=1; Not reported	72; 0	Robotics	25 minutes/session, 5 days/week, 25 sessions in total, in addition to conventional therapy	Effectiveness, body functions (ADL)	—
Hicks [63], 2016, United States	Quantitative; nonrandomized	Cardiac; n=285; Geriatric rehabilitation	79 (48-99); 54.3	Health gateway vs usual care	Encouraged daily use, in addition to conventional therapy	Effectiveness, activities (ADL)	Effectiveness, external factors
Iosa [46], 2015, Italy	RCT	Stroke; n=4; Hospital, rehabilitation unit	71.5 (4.51); 50	Exergames in combination with virtual reality vs usual care	30 minutes/session, 3 days/week, in addition to conventional therapy	Feasibility (motivation, time spent, adverse events)	Effectiveness, body functions, activities
Karner [55], 2019, Germany	RCT	Stroke; n=56.4%; Hospital, rehabilitation unit	73,7 (7.33); 56.4	Robotics vs book reading	30 minutes/session 3 days/week over 3 weeks	Effectiveness, body functions (visual)	—
Koneva [67], 2018, Russia	Quantitative; nonrandomized	Stroke; n=40; Not reported	84 (1.2); 30	Virtual reality vs usual care	Task-specific training	Effectiveness, body functions (neurological)	Effectiveness, body functions, activities, participation
Laver [50], 2012, Australia	RCT	Multiple diagnoses; n=44; Hospital, rehabilitation unit	84.9 (4.5); 80	Exergames vs usual care	25 minutes/session, 5 days/week for duration of stay	Effectiveness, activities (mobility)	Effectiveness, body functions, activities, participation
Levinger [64], 2016, Italy	Quantitative; nonrandomized	Orthopedic; n=4; Hospital, rehabilitation unit	70; 76	Exergames vs usual care	2 sessions/week, in addition to conventional therapy	Effectiveness, activities (mobility)	Effectiveness, body functions, activities, participation
Li [54], 2020, Hong Kong	RCT	Orthopedic; n=31; Ambulatory	79,3 (9.1); 80.6	Mobile apps vs usual care	Use of app based on rehabilitation goals, in addition to conventional therapy	Effectiveness, activities (mobility)	Effectiveness, feasibility, body functions, activities,
Ling [58], 2017, Netherlands	Mixed methods	Orthopedic; n=7; Geriatric rehabilitation	70 (8); 71	Exergames	30 minutes/session, in addition to conventional therapy	Usability (ease of use)	—
Marschollek [75], 2014, Germany	Quantitative; nonrandomized	Orthopedic; n=14; Ambulatory	83.5 (71-90)	Health sensors	Sensors placed at home for monitoring ADL	Feasibility (installation time, downtimes)	Acceptability
Oesch [49], 2017, Switzerland	RCT	Multiple diagnoses; n=54; Geriatric rehabilitation	74 (67-79); 45	Exergames vs self-regulated exercises	30 minutes/session, twice a day	Effectiveness (personal factors)	Effectiveness personal factors, activities
Peel [40], 2016, Australia	RCT	Multiple diagnoses; n=270; Geriatric rehabilitation	81 (8); 58	Health sensors with goal-setting vs health sensors without goal-setting	Daily feedback and goal-setting by therapists, in addition to conventional therapy	Effectiveness, activities (mobility)	Effectiveness, activities, participation, external factors
Peel [78], 2011, Australia	Quantitative; nonrandomized	Multiple diagnoses; n=0; Ambulatory	—	Video communication	All communication conducted through intervention	Feasibility	—
Piqueras [47], 2013, Spain	RCT	Orthopedic; n=142; Ambulatory	73.3 (6.5); 72.4	Video communication in combination with health sensors vs usual care	1 hour/session over 10 days	Effectiveness, body functions (mobility of joints)	Effectiveness, body functions, activities
Pol [48], 2019, Netherlands	RCT	Orthopedic; n=240; Ambulatory	83 (6.9); 79.6	Health sensors in combination with non-eHealth intervention vs non-eHealth intervention vs usual care	Sensors placed at home for monitoring ADL, 4 home visits and 4 telephone consultations	Effectiveness, activities (other)	Effectiveness, participation
Sampson [73], 2012, New Zealand	Quantitative; nonrandomized	Stroke; n=1; Rehabilitation center	76; 100	Robotics in combination with virtual reality	45 minutes/session, 4 sessions/week over 6 weeks, in addition to conventional therapy	Effectiveness, body functions (muscle power, tone, and reflexes)	Effectiveness body functions
Schoone [45], 2011, Netherlands	RCT	Stroke; n=24; Geriatric rehabilitation	71.3 (8.2); 33	Robotics	10-30 minutes/sessions, 3 sessions/week over 6 weeks, in addition to conventional therapy	Effectiveness, body functions, activities (hand and arm use)	Effectiveness participation, external factors
Schwickert [74], 2011, Germany	Quantitative; nonrandomized	Orthopedic; n=8; Geriatric rehabilitation	79.5; 50	Robotics, virtual reality	30-45 minutes/session, 2-3 sessions/week for 2-4 weeks, in addition to conventional therapy	Feasibility (adherence, satisfaction)	Effectiveness, body functions, activities, participation
Takano [79], 2020, Japan	Quantitative; nonrandomized	Orthopedic; n=27; Hospital, rehabilitation unit	81 (6.3); 89	Robotics in combination with exergames	20 min/session 6 sessions/week for 2 weeks in addition to conventional therapy	Effectiveness activities (mobility)	Effectiveness, activities,
Taveggia [43], 2016, Italy	RCT	Stroke; n=28; Hospital, rehabilitation unit	72 (6); 39	Robotics vs usual care	30 minutes/session, 5 sessions/week over 5 weeks, in addition to conventional therapy	Effectiveness, activities (mobility)	Effectiveness, activities, participation
Tousignant [76], 2006, Canada	Quantitative; nonrandomized	Multiple diagnoses; n=4; Ambulatory	70,75; 50	Video communication	1 hour/session, 3 sessions/week over 4 weeks	Effectiveness, activities (ADL)	Effectiveness, body functions, activities
Van den Berg [51], 2015, Australia	RCT	Multiple diagnoses; n=58; Hospital, rehabilitation unit	80 (12); 62	Exergames vs usual care	1 hour/session, 5 session/week, in addition to conventional therapy	Effectiveness, activities (mobility)	Usability; Effectiveness, activities, participation
Vanoglio [41], 2017, Italy	RCT	Stroke; n= 30; Hospital, rehabilitation unit	71 (12); 53	Robotics vs usual care	40 minutes/session, 5 sessions/week over 6 weeks	Feasibility (n completed, adverse events, difficulty)	Effectiveness, body functions, external factors
White [60], 2015, Australia	Qualitative	Stroke; N=12; Rehabilitation center, ambulatory	73 (53-83); 33	Mobile apps	Therapist installed apps; patients encouraged to explore iPad	Usability	—
Yoshikawa [66], 2018, Japan	Quantitative; nonrandomized	Orthopedic; n=19; Hospital	76 (6.85); 81	Robotics vs usual care	14 minutes/session, 12-14 session in 4 weeks, in addition to conventional therapy	Effectiveness, activities (mobility)	Effectiveness, body functions

^a^RCT: randomized controlled trial.

^b^Not available.

^c^BS: Borg Perceived Exertion Scale.

^d^%MHR: maximum heart rate.

^e^ADL: activities of daily living.

Of 40 studies, 17 (43%) included participants who were diagnosed with stroke [41-46,55-57,60,67-73], 10 (25%) included participants with multiple diagnoses [40,49-52,59,61,62,76-78], 11 (28%) included participants with orthopedic problems [47,48,54,58,59,64-66,74,75,79], and 2 studies (5%) included participants with cardiac-related diagnoses [53,63]. Across all studies, the included sample size ranged from 1 to 285 participants.

Various types of eHealth interventions were used. Of 40 studies, 11 studies (28%) delivered the intervention via robotics [41-43,45,55,56,65,66,70-72], 2 studies (5%) combined robotics with virtual reality [73,74], and 1 study (3%) combined robotics with exergames [79]. Additionally, 9 studies (of 40, 23%) investigated exergames [44,46,49-51,58,62,64,77], of which 2 (of 9, 22%) combined exergames with virtual reality [44,46] and 1 (of 9, 11%) combined exergames with health sensors [51]. Of 40 studies, 2 (5%) examined video communication [76,78], 3 (8%) combined video communication with health sensors [47,53,69], and 1 (3%) combined video communication with a non-eHealth intervention [52]. Of 40 studies, health sensors were used in 6 studies (15%) [40,48,57,61,63,75], including 1 (of 6, 17%) in combination with a health gateway [63] and 1 (of 6, 17%) in combination with a non-eHealth intervention [48]. Of 40 studies, 3 studies (8%) investigated mobile apps [54,59,60], and 2 studies (5%) examined virtual reality [67,68].

Outcome measures related to effectiveness were reported in 24 of 40 studies (60%) [40,42-45,47-50,53,55,56,61,63-68, 70,72,73,76,79], and 10 of 40 studies (25%) included outcome measures related to effectiveness and feasibility [41,46,52,54,57,62,69,71,74,77]. Of 40 studies, 2 studies (5%) included outcomes related to usability [58,60], 2 studies (5%) included outcomes related only to feasibility [75,78], 1 study (3%) included outcomes related to effectiveness and usability [51], and 1 study (3%) included outcomes related to feasibility and usability [59]. A detailed description of all included studies can be found in Multimedia Appendix 3.

### Study Quality

**Figure 2:**
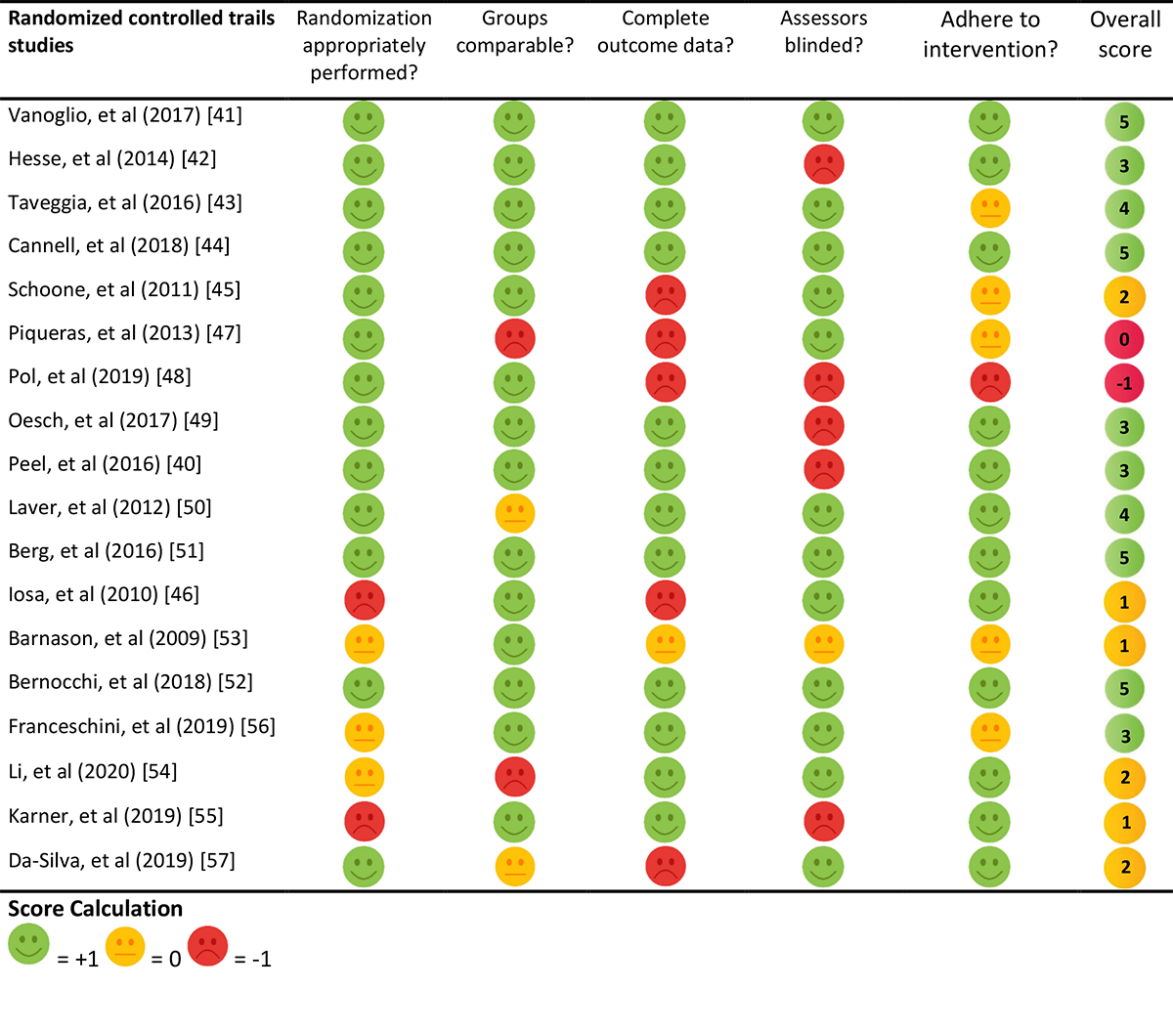
Quality appraisal for randomized controlled trial studies.

**Figure 3:**
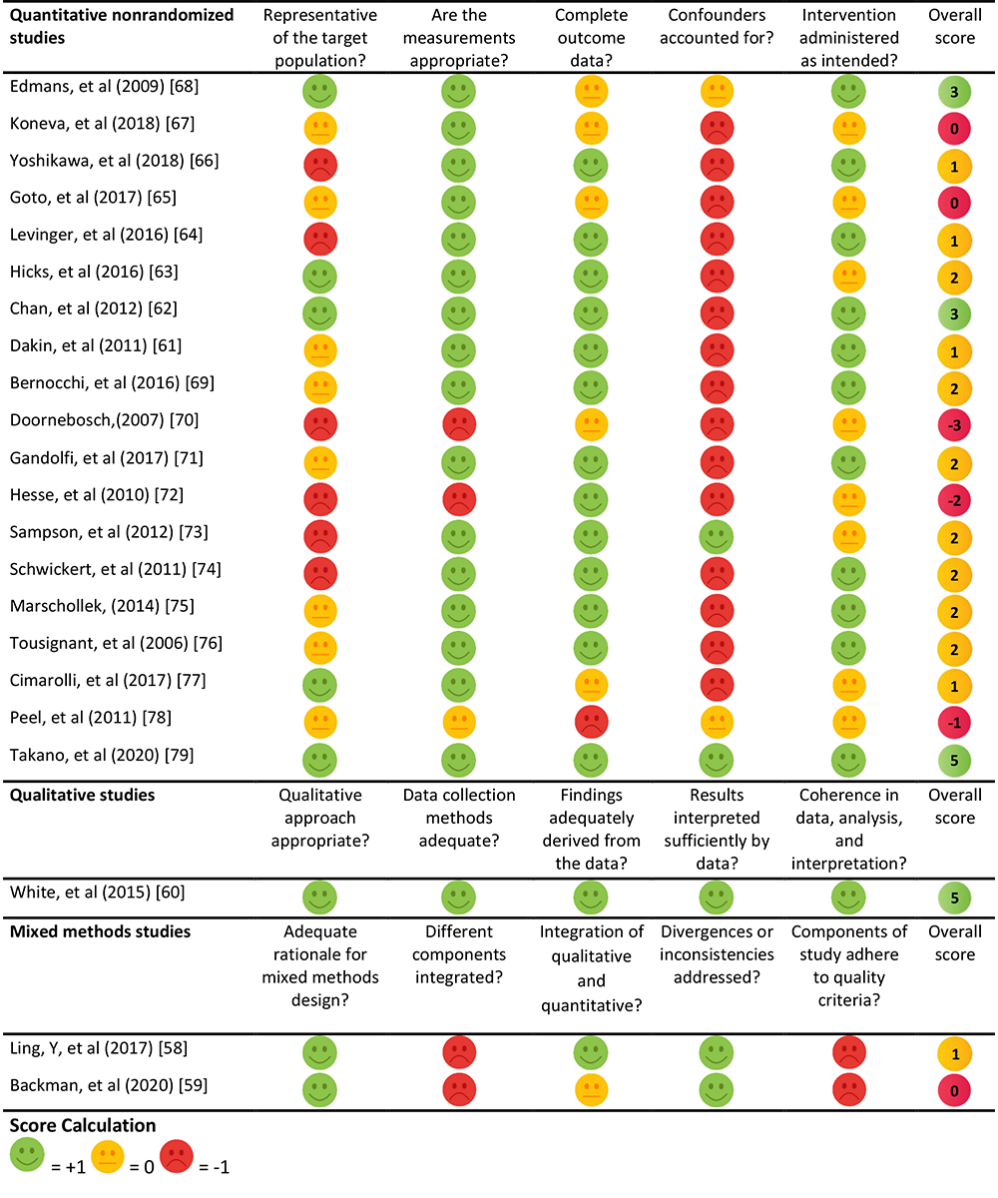
Quality appraisal for quantitative nonrandomized, qualitative, and mixed methods studies.

Results of the quality assessment are presented in Figure 2 and Figure 3. The quality of the included studies ranged from –3 to 5 (on a scale ranging from –5 to 5). The mean overall score was 3 for randomized controlled trails, 1 for quantitative nonrandomized studies, 1 for a mixed methods studies, and 5 for a qualitative study (based on 1 study). In quantitative nonrandomized studies, the most frequent shortcoming was insufficient reporting of confounders; only 2 of 19 studies (11%) accounted for confounders in design and analysis [73,79]. The representativeness of the target population in quantitative nonrandomized studies was also often insufficient; 9 of the 19 studies (47%) reported insufficient information, lacking either adequate explanation of why certain eligible participants chose not to participate or a clear description of the target population [53,61,65,67,69,71,75,76,78]. Additionally, 6 of the 19 studies (32%) included a sample size of less than 20 [64,66,70,72-74].

### Effectiveness

#### Main Results for Effectiveness

**Figure 4:**
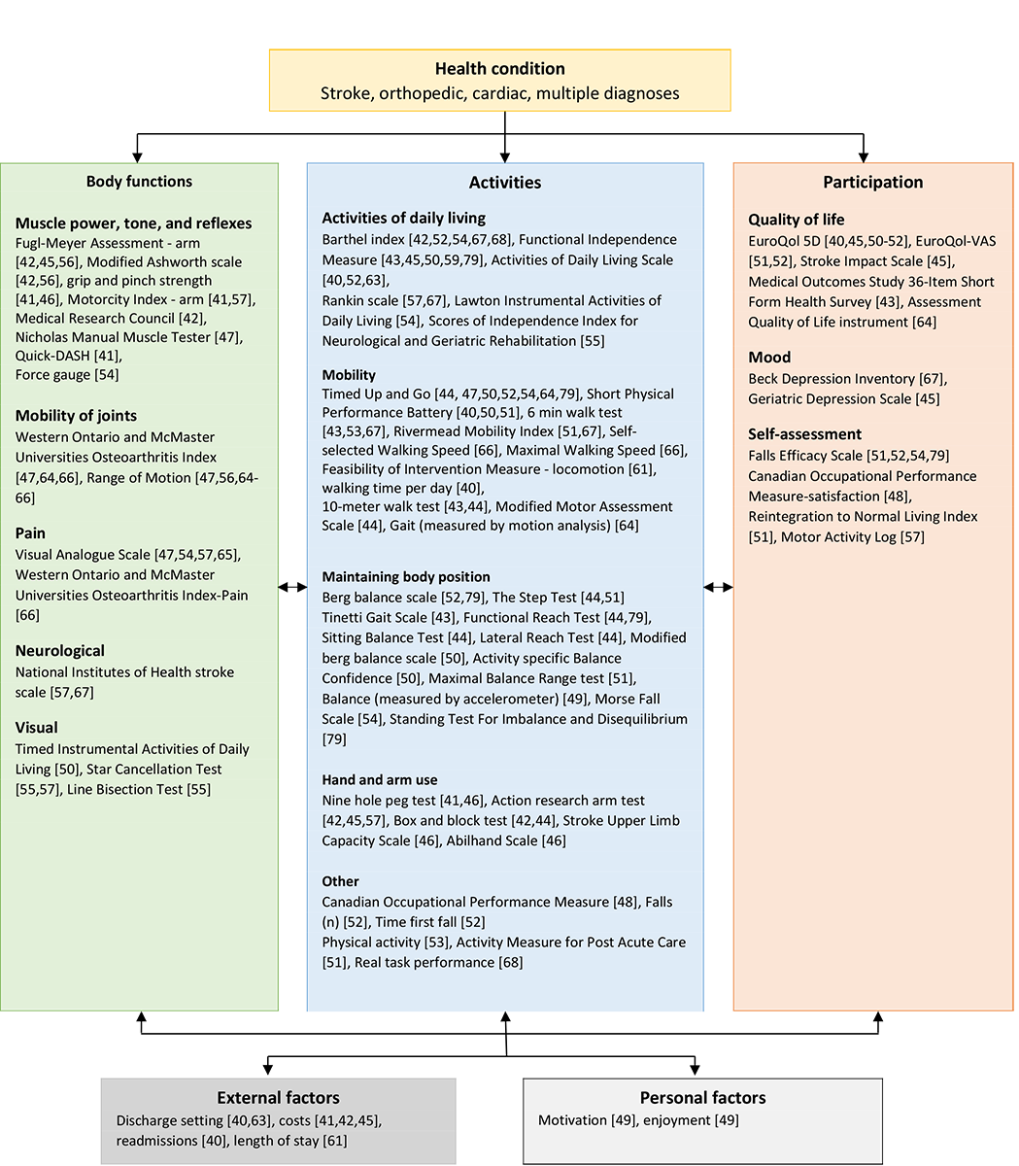
Outcome measures classified by the International Classification of Functioning, Disability, and Health model.

**Figure 5:**
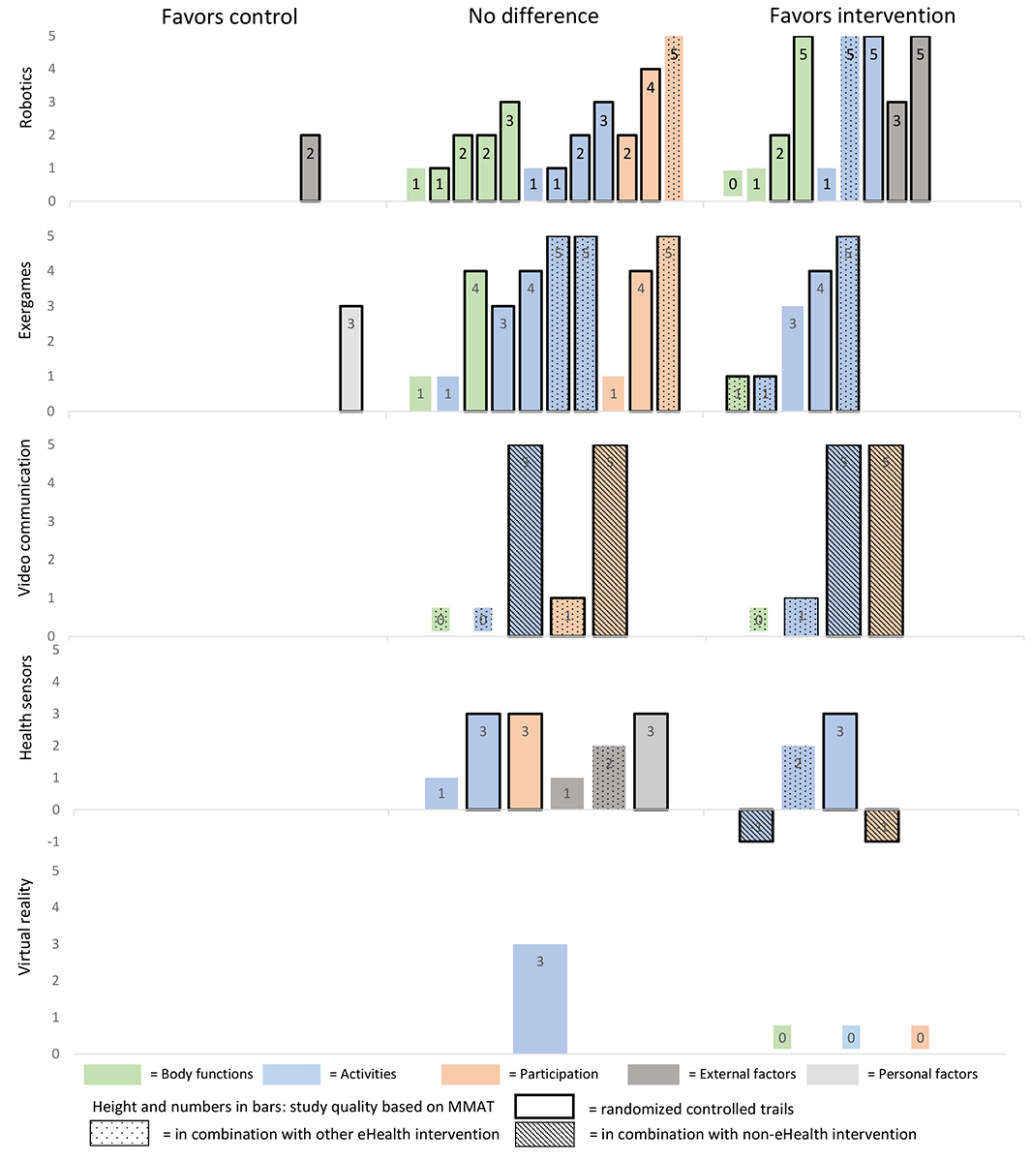
Harvest plot: effectiveness of eHealth interventions. MMAT: Mixed Methods Appraisal Tool.

Across all studies with a control group (n=27; 27/40, 68%), 73 different outcome measures were reported that were related to effectiveness, including 16 (22%) within the ICF domain “body functions,” 40 (55%) in the domain “activities,” 11 (15%) in the domain “participation,” 4 (5%) in the domain “external factors,” and 2 (3%) in the domain “personal factors” (Figure 4). In 15 studies (of 27, 56%), eHealth interventions were found to be at least as effective as non-eHealth interventions when focusing on the primary outcome measure, and 11 studies (of 27, 41%) reported eHealth interventions to be more effective than non-eHealth interventions. Of 27 studies, 1 study (4%) reported beneficial outcomes in favor of the non-eHealth interventions. Results for each ICF domain are described in detail below. A harvest plot illustrating the evidence regarding effectiveness is presented in Figure 5.

#### Body Functions

Of 40 total studies, 14 studies (35%) included 16 outcomes related to body functions [41,42,45-47,50,54-57,64-67]. Of these 14 studies, 9 studies (64%) found, in 7 outcome measures, significant improvements in favor of the intervention group (Figure 5) [41,46,47,54-56,65-67]. Of 14 studies, 4 studies (29%) reported improved muscle power through robotics [56,65], exergames [46], or mobile apps [54]. Of 14 studies, 4 studies (29%) found that the addition of robotics [56,65,66] or video communication in combination with health sensors [47] improved the mobility of joints when compared with physical therapy alone. Another 2 studies (of 14, 14%) reported that the use of robotics could decrease pain when compared with conventional physiotherapy [65,66]. Koneva and colleagues [67] reported that the use of virtual reality improved neurological status, as measured by the National Institutes of Health stroke scale, when compared with usual care (5.2±0.4 vs 6.3±0.5; *P*<.001).

#### Activities

Of all 40 included studies, 25 studies (63%) reported 40 outcomes related to activities [40-55,57,61-64,66-68,79], and 13 studies (33%) found, in 17 outcomes, a significant outcome in favor of the intervention group [40,41,46,48,50-53,62,63,66,67,79]. Of 40 studies, 5 studies (13%) demonstrated that eHealth was effective in improving activities of daily living when the intervention was delivered via video communication in combination with health sensors and a non-eHealth intervention [52] or when the intervention was delivered via health sensors in combination with health gateways [63], exergames [62], robotics [79], or virtual reality [67]. In these studies, eHealth was compared with usual care [52,67], physiotherapy [62,79], or no intervention [63]. Another 6 studies (of 40, 15%) found that eHealth could contribute to improved mobility through the use of robotics [52,79], exergames [50], virtual reality [67], video communication in combination with health sensors [52], or health sensors in combination with goal setting [40]. These interventions were compared with physiotherapy [50,66,79], usual care [52,67], or health sensors without goal setting [40]. Of 40 studies, 4 studies (10%) reported improvements in balance when the intervention was delivered via robotics [79], exergames [50], exergames in combination with health sensors [51], or video communication in combination with health sensors [52], when compared with physiotherapy [50,51,79] or usual care [52]. Another 2 studies (of 40, 5%) reported that either robotics [41] or exergames in combination with health sensors [46] could improve hand and arm function when compared with physiotherapy [41] or no intervention [46]. Pol and colleagues [48] found that patient-reported daily functioning significantly improved with the use of health sensors in combination with cognitive behavioral treatment, compared with cognitive behavioral treatment alone, reporting a difference of 1.17 (95% CI 0.47-1.87; *P*<.001). Bernocchi and colleagues [52] reported that the use of video communication in combination with health sensors and a non-eHealth intervention was effective in preventing falls in patients who were at high risk of falling, when compared with usual care (29 falls vs 56 falls; *P*<.001). Of 40 studies, 1 study (3%) demonstrated that the use of video communication in combination with health sensors improved physical activity when compared with usual care [53].

#### Participation

Of 40 studies, 12 studies (30%) included 11 outcome measures within the participation domain [40,43,45,48,50-53,57,64,67,79]. Of these 12 studies, 3 studies (27%) reported a significant difference in quality of life [52], mood [67], or self-assessment [48] when the intervention was delivered via the use of video communication in combination with health sensors and a non-eHealth intervention [52], virtual reality [67], or the use of health sensors in combination with a non-eHealth intervention [48]. Particularly, Bernocchi and colleagues [52] demonstrated that the use of video communication in combination with health sensors and a non-eHealth intervention significantly improved scores on the EuroQol Visual Analog Scale at 6 months, when compared with usual care (mean 63.8 vs mean 53.5; *P*<.001). Koneva and colleagues [67] reported that the use of virtual reality decreased the severity of depression as measured by the Beck Depression Inventory, when compared with usual care (mean 9.5, SD 5.52 vs mean 10.3, SD 6.03; *P*<.05). Additionally, Pol and colleagues [48] found that the use of health sensors in combination with a non-eHealth intervention significantly improved the performance satisfaction in daily functioning at 6 months, when compared with usual care, reporting a difference of 0.94 (95% CI 0.37-1.52; *P*<.001).

#### External Factors

Across all 40 studies, 5 studies (13%) included outcome measures related to external factors [40,42,45,61,63]. Of these 5 studies, 2 studies (40%) included robotics as interventions and found significant differences in cost, in favor of the intervention group [41,42]. Of the 5 studies, 1 study (20%) included robotics as an intervention and found a difference in favor of the control group [45]. Hesse and colleagues [42] and Vanoglio and colleagues [41] reported decreases in cost with the use of robotics in comparison with either regular arm therapy (€4.15 [US $4.92] for robotic interventions vs €10.00 [US $11.85] for regular arm therapy, for each patient per session) [42] or physiotherapy (€237 [US $280.73] for robotic intervention vs €480 [US $568.57] for physiotherapy, for each patient per 30 days) [41]. In contrast, Schoone and colleagues [45] reported an increase in total costs when compared with physiotherapy (€644.14 [US $762.99] for robotic interventions vs €423.74 [US $501.93] for physiotherapy). Across all studies, no differences were found with regard to discharge settings [40,63], readmissions [40], or lengths of stay [61].

#### Personal Factors

Oesch and colleagues [49] found that self-regulated exercise using instruction leaflets was superior to exergames in terms of enjoyment (effect size: 0.88, range 0.32-1.44; *P*<.001) and motivation (effect size: 0.59, range 0.05-1.14; *P=*.046).

### Feasibility

#### Main Results for Feasibility

Of the 40 included studies, 20 studies (50%) evaluated the feasibility of the eHealth intervention used [41,46,50-52,54,57,59,60,62,64,65,69,71,72,74-78], of which 19 (of 20, 95%) concluded that the eHealth intervention was feasible when it was delivered via robotics [41,65,71,72], robotics in combination with exergames [74], exergames [50,62,64,77], exergames in combination with health sensors [46,51], video communication [76], video communication in combination with health sensors [52,69], health sensors [57], health gateways in combination with health sensors [75], or mobile apps [54,59,60]. Peel and colleagues [78] reported that the use of video communication was not feasible due to problems related to patient limitations, staff issues, and the logistics of the system.

The outcome measures applied to evaluate feasibility varied considerably among studies, and a total of 19 different outcome measures were used. Of the 20 studies that reported feasibility, 6 studies (30%) reported outcomes related to “adverse events,” 7 studies (35%) reported outcomes related to “adherence,” and 7 studies (35%) reported outcomes related to “exclusion rate.” Another 4 studies (of 20, 20%) did not specify the outcome measure used to evaluate feasibility but used outcomes related to effectiveness to establish feasibility [54,64,65,72].

#### Adverse Events

None of the included studies reported serious adverse events during the study period [41,46,50,51,74,76]. However, 2 studies (of 40, 5%) reported that some participants experienced discomfort during exergames [49,50].

#### Adherence

Of 40 studies, adherence was reported in 7 studies (18%) [49-52,57,74], and 5 studies (13%) reported information regarding the number of completed sessions [41,50-52,69]. Of the 7 studies reporting adherence, 5 studies (71%) reported high levels of adherence, ranging from 76% [52] to 100% [74]. Of the 7 studies, 2 studies (29%) reported low adherence in patients assigned to an exergame intervention when compared with either a non-eHealth intervention [49] or use of the exergame intervention below the recommended level (<30 minutes per week) [77].

#### Exclusion Rate

Of 40 studies, high exclusion rates were found in 7 studies (18%). Specifically, of these 7 studies, 1 study (14%) reported an exclusion rate of 64% [47], 2 studies (29%) reported an exclusion rate of 75% [49,51], and 4 studies (57%) reported an exclusion rate over 80% [42,45,50,68]. In these latter studies, eHealth was delivered through complex eHealth interventions: robotics [42,45], exergames [50], and virtual reality [68]. The most commonly reported reasons for exclusion were cognitive impairment [45,47,49,51], physical impairment [45,49], and refusal to participate [42,47,49-51,68]. Of these 7 studies, in 2 studies (29%), the reason given for declining to participate was “no interest” in eHealth [50,51].

### Usability

#### Main Results for Usability

Of 40 studies, outcomes related to the usability of eHealth interventions were addressed in 4 studies (10%): 2 studies (5%) evaluated the usability of exergames [51,58], and another 2 studies (5%) evaluated mobile apps [59,60]. Evaluation of usability consisted of a system usability scale [51], a survey of patients and therapists [58,59], or semistructured interviews [59,60]. Of the 4 studies that reported usability, 2 studies (50%) included outcomes related to the barrier “cognition,” 4 studies (100%) included outcomes related to the aging barrier “motivation,” and 1 study (25%) included outcomes related to the barrier “physical ability.” None of the studies included outcomes related to the barrier “perception.”

#### Cognition

Ling and colleagues [58] reported that some patients found exergames too complicated because of the requirement to engage in multiple activities simultaneously, and they experienced difficulties in following instructions. To tailor the exergames to older patients with cognitive impairments, the authors advised to minimize the amount of information presented on the screen, which might help older patients to perceive the information better [58]. Additionally, White and colleagues [60] reported that patients with cognitive impairments experienced difficulties in operating mobile apps and needed their partner for support.

#### Motivation

Van den Berg and colleagues [51] reported a mean score of 62 (SD 21), on the system usability scale (scores ranging from 0 to 100), indicating that participants were generally comfortable with exergames and that they would like to use exergames more frequently. Similar findings were reported by Ling and colleagues [58], who concluded that patients and therapists both found exergames easy to use and therapists intended to use the exergame in the future. Therapists rated the exergame as highly satisfactory for motor rehabilitation in older patients after hip surgery. Findings regarding mobile apps indicated that patients readily grasped the skills required for use and that this was a beneficial source of extrinsic motivation [59,60].

#### Physical Ability

Ling and colleagues [58] reported that some patients with physical disabilities had difficulties playing certain exergames that required stepping exercises because these patients were unable to maintain balance during exergames.

## Discussion

### Principal Findings

This review aimed to provide an overview of the effectiveness, feasibility, and usability of eHealth in geriatric rehabilitation. The review included 40 studies that applied eHealth interventions in older patients receiving geriatric rehabilitation. The majority of the included studies showed that eHealth interventions in geriatric rehabilitation are at least as effective as non-eHealth interventions. All studies that delivered eHealth in combination with another non-eHealth intervention reported positive outcomes. Most studies included outcome measures related to the ICF domain “activities.” Very few studies included outcomes related to the ICF domain “participation.” eHealth seems to be feasible in geriatric rehabilitation, since no serious adverse events were reported and most studies reported high levels of adherence. However, high exclusion rates were found in some studies. Results related to usability indicate that there are certain age-related barriers, such as cognition and physical ability, that lead to difficulties in using eHealth. Very few studies included outcomes related to feasibility and usability. However, these are important prerequisites to maximize the likelihood of successful implementation, and they thereby influence the effectiveness of eHealth.

### Comparison With Prior Work

Our findings suggest that eHealth delivered via robotics, exergames, or health sensors is often found to be at least as effective as non-eHealth. Previous reviews that examined robotics [80], exergames [16], or health sensors [81,82] often found more beneficial results in favor of the intervention group. These reviews did not focus on older adults who were admitted for geriatric rehabilitation, and this could indicate that there are certain age-related barriers that affect the effectiveness of eHealth in older adults receiving geriatric rehabilitation. All of the included studies that delivered eHealth in combination with a non-eHealth intervention reported beneficial outcomes in favor of the intervention group. This is in line with other studies in which eHealth was delivered in combination with a non-eHealth intervention [83-85]. This indicates that eHealth is more beneficial when provided through blended care, where eHealth is delivered in combination with face-to-face treatment. This may provide a better quality of care by combining the best of the two types of interventions. This seems to especially be the case when blended care is delivered via video communication [52] or health sensors [48], since it offers the possibility to monitor and treat patients remotely.

Almost all of the studies that included outcomes related to feasibility concluded that eHealth was feasible in older adults receiving geriatric rehabilitation. None of the studies reported serious adverse events, which is in line with other reviews concerning feasibility of exergames [15,86]. The majority of the studies that included outcomes related to adherence or completed sessions reported high levels of adherence. Previous reviews that examined exergames also reported high adherence rates [86]. Some studies where eHealth was delivered via robotics or exergames reported a high exclusion rate (up to 88%). All studies with exclusion rates of ≥75% were conducted in a geriatric rehabilitation setting [45,49] or in a hospital with a dedicated rehabilitation unit [50,51]. Reasons for exclusion were mostly cognitive or physical impairments, problems that are often present in older patients receiving geriatric rehabilitation. These findings indicate that eHealth in geriatric rehabilitation is safe to use and overall adherence is expected to be high, but complex eHealth interventions such as robotics and exergames might only be feasible in a selective group of older patients receiving geriatric rehabilitation.

There is limited available evidence on the usability of eHealth interventions. The studies included in our review indicate that exergames and mobile apps are usable once older patients have been trained in their use. However, there were certain age-related barriers associated with cognitive or physical ability that led to difficulties in using eHealth. While we did not find studies that reported problems in the use of eHealth due to problems in perception, 2 of 4 studies (50%) that included usability outcome measures explicitly excluded patients with visual impairments [51,58]. This might suggest that poor usability was expected in patients with visual impairments; this is in line with findings from other studies [27]. These findings suggest that usability problems are expected in older patients receiving geriatric rehabilitation, since they often suffer from cognitive, physical, or visual impairments. eHealth should be tailored to these specific age-related barriers to maximize the probability of successful use and implementation [22,27]. Furthermore, most studies did not incorporate clear usability endpoints, and the evaluation of usability varied considerably among studies. The lack of using clear endpoints or reliable and validated questionnaires combined with task metrics (preferably, task completion) to evaluate usability hampers the ability to pinpoint usability issues and prevents comparisons across different eHealth types [25,87].

### Strengths and Limitations

The first strength of this review is the extensive search strategy that covered a broad range of search databases and included all types of research designs. Another strength of this review is the categorization of outcome measures based on the ICF model, providing a clear overview of different types of outcome domains evaluated in the included studies. Nonetheless, several limitations of this systematic review should be noted. While this review provides a broad overview of the literature on 3 different concepts, our study design led to a vast variety of different outcome measures related to effectiveness. The inclusion of various outcomes measures, in combination with various eHealth interventions and diagnoses, limited our ability to draw definitive conclusions. Since a meta-analysis was not feasible, we were unable to report an effect size and publication bias. We instead provided an overview of the effectiveness of eHealth interventions using a harvest plot. Lastly, while we used a separate search string that included keywords related to usability, we only found 4 studies that included outcomes on usability. A possible explanation might be that we did not include specific Computer Science search databases, which might include more studies that are related to usability [88]. Furthermore, despite the massive growth in eHealth studies, only a small portion publish their usability results [89].

### Conclusions

In conclusion, eHealth can improve rehabilitation outcomes in older adults receiving geriatric rehabilitation. Based on our findings, comparisons to literature, and the strengths and limitations of our review, our main results and recommendations for further research and the use of eHealth in clinical practice are (1) keep it simple, (2) include evidence on usability, (3) focus on participation, and (4) ensure consensus. First, simple interventions have the most potential to improve rehabilitation outcomes in older adults receiving geriatric rehabilitation, especially, when they are provided as blended care. Additionally, simple eHealth interventions have a higher chance of feasibility in older patients receiving geriatric rehabilitation who often suffer from cognitive or physical impairments. Second, scarce evidence on the usability of eHealth might hamper the implementation of eHealth in older patients receiving geriatric rehabilitation and could negatively influence effectiveness and feasibility. Further research on this topic with clear endpoints is needed. Health care professionals need to be aware of the usability of eHealth interventions they are providing. Third, participation is a key concept in geriatric rehabilitation and plays an important role in enabling older patients to continue living as independently as possible. Future research on eHealth interventions should consider including outcome measures related to participation. Fourth, current evidence on the use and evaluation of eHealth in geriatric rehabilitation is diverse, making it hard to compare outcomes and draw evident conclusions. Consensus on the use and evaluation of eHealth is needed for further development and implementation of eHealth in geriatric rehabilitation.

## Appendix

Multimedia Appendix 1 PRISMA checklist.

Multimedia Appendix 2 Keyword strings used for searching databases.

Multimedia Appendix 3 Detailed description of all included studies.

## Abbreviations

ICF: International Classification of Functioning, Disability, and Health.
MMAT: Mixed Methods Appraisal Tool.
PRISMA: Preferred Reporting Items for Systematic Reviews and Meta-analyses.
