# Supplementary material for: eHealth in Geriatric Rehabilitation: Systematic Review of Effectiveness, Feasibility, and Usability
Source: J Med Internet Res. 2021 Aug 19;23(8):e24015. doi: 10.2196/24015 (PMC8414304; doi:10.2196/24015)
Supplement: Multimedia Appendix 2 [file jmir_v23i8e24015_app2.pdf]

Effectiviteit eHealth in de geriatrische revalidatiezorg.

+ usability

27564857 29289643

1. Effectiveness of eHealth interventions for the promotion of physical activity in older adults: A systematic review. Muellmann S1, Forberger S2, Möllers T3, Bröring E4, Zeeb H5, Pischke CR6. Prev Med. 2018 Mar;108:93-110. doi: 10.1016/j.ypmed.2017.12.026. Epub 2017 Dec 28.
2. Peel NM, Paul SK, Cameron ID, Crotty M, Kurrle SE, Gray LC. Promoting Activity in Geriatric Rehabilitation: A Randomized Controlled Trial of Accelerometry. PLoS One. 2016 Aug 26;11(8):e0160906.

Databases:

PubMed
http://www.ncbi.nlm.nih.gov/pubmed?otool=leiden

8-1-2021

Concreet gaat het om het volgende:
- (1) een herhaling van beide zoekstrings waarbij de nadruk wordt gelegd op alle drie de componenten (eHealth AND ouderen AND revalidatie) of (2) vier componenten in geval van usability
- (3) Een nieuwe zoekstring met een omschrijving van de term feasibility. Ook weer met de nadruk op alle vier de componenten

1.

(("Rehabilitation"[majr] OR rehab*[ti] OR "Rehabilitation Nursing"[majr] OR "Rehabilitation Centers"[majr] OR "Activities of Daily Living"[ti] OR "Animal Assisted Therapy"[ti] OR "Equine-Assisted Therapy"[ti] OR "Art Therapy"[ti] OR "Bibliotherapy"[ti] OR "Correction of Hearing Impairment"[ti] OR "Total Communication Methods"[ti] OR "Lipreading"[ti] OR "Manual Communication"[ti] OR "Dance Therapy"[ti] OR "Early Ambulation"[ti] OR "Exercise Therapy"[ti] OR "Endurance Training"[ti] OR "Continuous Passive Motion Therapy"[ti] OR "Muscle Stretching"[ti] OR "Plyometric Exercise"[ti] OR "Resistance Training"[ti] OR "Music Therapy"[ti] OR "Occupational Therapy"[ti] OR "Recreation Therapy"[ti] OR "Language Therapy"[ti] OR "Myofunctional Therapy"[ti] OR "Speech Therapy"[ti] OR "Alaryngeal Speech"[ti] OR "Voice Training"[ti] OR "Telerehabilitation"[ti] OR "Activity of Daily Living"[ti] OR "Plyometric Exercises"[ti] OR "Exercise"[majr] OR "exercise"[ti] OR "exercises"[ti] OR "physical activity"[ti]) AND ("Aged"[mesh] OR "elderly"[ti] OR "elder"[ti] OR "elders"[ti] OR geriatr*[ti] OR "Homes for the Aged"[majr] OR "Health Services for the Aged"[majr] OR "Senior Centers"[majr] OR older person*[ti] OR old person*[ti] OR older patient*[ti] OR old patient*[ti] OR "older women"[ti] OR "old women"[ti] OR "older men"[ti] OR "old men"[ti] OR old adult*[ti] OR older adult*[ti]

OR "Older individual"[ti] OR "Older individuals"[ti] OR "old people"[ti] OR "older people"[ti] OR "Oldest Old"[ti] OR "Nonagenarians"[ti] OR "Nonagenarian"[ti] OR "Octogenarians"[ti] OR "Octogenarian"[ti] OR "Centenarians"[ti] OR "Centenarian"[ti] OR "septuagenarian"[ti] OR "septuagenarians"[ti] OR "Aging"[majr] OR "aging"[ti] OR "ageing"[ti] OR "older population"[ti] OR "aging population"[ti] OR "aging population"[ti] OR geront*[ti] OR "old-aged"[ti] OR "old-age"[ti] OR "old aged"[ti] OR "old age"[ti]) AND ("accelerometers"[ti] OR "accelerometer"[ti] OR "accelerometry"[ti] OR acceleromet*[ti] OR "Telemedicine"[majr] OR "web portals"[ti] OR "web portal"[ti] OR web portal*[ti] OR e-consult*[ti] OR econsult*[ti] OR telemed*[ti] OR "ehealth"[ti] OR "e-health"[ti] OR "mhealth"[ti] OR "m-health"[ti] OR "mobile health"[ti] OR "telehealth"[ti] OR "tele-health"[ti] OR "tele health"[ti] OR **electronic communication*[ti]** OR "remote consultation"[ti] OR "remote health care"[ti] OR "remote healthcare"[ti] OR "remote care"[ti] OR "remote monitoring"[ti] OR "teleconsultation"[ti] OR teleconsult*[ti] OR **mobile*[ti] OR "webbased"[ti] OR "web-based"[ti] OR "Electronic Mail"[majr] OR "Electronic Mail"[ti] OR e-mail*[ti] OR email*[ti]** OR "mobile apps"[ti] OR "mobile app"[ti] OR webapp*[ti] OR "SMS"[ti] OR "Cell Phones"[ti] OR "Smartphone"[ti] OR "Text Messaging"[ti] OR "Cell Phone"[majr] OR "Cell Phone"[ti] OR "Smartphones"[ti] OR iphon*[ti] OR text messag*[ti] OR "texting"[ti] OR **"mobile"[ti]** OR "cellular phone"[ti] OR "cellular phones"[ti] OR "smart phone"[ti] OR "telemedicine"[ti] OR "tele-care"[ti] OR "telecare"[ti] OR "tele-monitoring"[ti] OR "telemonitoring"[ti] OR **"website"[ti] OR "websites"[ti]** OR "personal digital assistant"[ti] OR "computer-assisted instruction"[ti] OR "ipad"[ti] OR ipad*[ti] OR "telenursing"[ti] OR telenurs*[ti] OR "virtual community"[ti] **OR "webpage"[ti] OR "webpages"[ti]** OR "web application"[ti] OR "web applications"[ti] OR "short message service"[ti] OR **"Internet"[majr] OR "internet"[ti] OR "online"[ti] OR "digital"[ti] OR digital*[ti]** OR "Reminder Systems"[majr] OR "Reminder Systems"[ti] OR "Reminder System"[ti] OR "Reminder Device"[ti] OR "Reminder Devices"[ti] OR "reminder messages"[ti] OR "reminder message"[ti] OR **"web"[ti]** OR "Virtual Reality"[majr] OR "Virtual Reality"[ti] OR "smart technology"[ti] OR smart technol*[ti] OR "wearable technology"[ti] OR "wearable technologies"[ti] OR "telerehabilitation"[ti] OR "Therapy, computer-assisted"[majr:noexp] OR "computer-assisted therapy"[ti] OR "computer assisted therapy"[ti] OR "online therapy"[ti] OR "Computer Mediated Communication"[ti] OR "Computer Mediated Communications"[ti] OR **"remote"[ti] OR "on-line"[ti] OR "on line"[ti]** OR "wearable devices"[ti] OR "wearable device"[ti] OR "healthsensor"[ti] OR "healthsensors"[ti] OR "health sensor"[ti] OR "health sensors"[ti] OR "Robotics"[majr] OR "robotics"[ti] OR robot*[ti] OR "exergame"[ti] OR "exergames"[ti] OR exergam*[ti] OR "Video Games"[majr] OR "app-based"[ti] OR "wearable app"[ti] OR "wearable apps"[ti] OR "wearables"[ti] OR "Nintendo"[ti] OR "Wii"[ti] OR "gaming console"[ti] OR "gaming consoles"[ti]))

(2) vier componenten in geval van usability

((("Rehabilitation"[majr] OR rehab*[ti] OR "Rehabilitation Nursing"[majr] OR "Rehabilitation Centers"[majr] OR "Activities of Daily Living"[ti] OR "Animal Assisted Therapy"[ti] OR "Equine-Assisted Therapy"[ti] OR "Art Therapy"[ti] OR

"Bibliotherapy"[ti] OR "Correction of Hearing Impairment"[ti] OR "Total Communication Methods"[ti] OR "Lipreading"[ti] OR "Manual Communication"[ti] OR "Dance Therapy"[ti] OR "Early Ambulation"[ti] OR "Exercise Therapy"[ti] OR "Endurance Training"[ti] OR "Continuous Passive Motion Therapy"[ti] OR "Muscle Stretching"[ti] OR "Plyometric Exercise"[ti] OR "Resistance Training"[ti] OR "Music Therapy"[ti] OR "Occupational Therapy"[ti] OR "Recreation Therapy"[ti] OR "Language Therapy"[ti] OR "Myofunctional Therapy"[ti] OR "Speech Therapy"[ti] OR "Alaryngeal Speech"[ti] OR "Voice Training"[ti] OR "Telerehabilitation"[ti] OR "Activity of Daily Living"[ti] OR "Plyometric Exercises"[ti] OR "Exercise"[majr] OR "exercise"[ti] OR "exercises"[ti] OR "physical activity"[ti]) AND ("Aged"[mesh] OR "elderly"[ti] OR "elder"[ti] OR "elders"[ti] OR geriatr*[ti] OR "Homes for the Aged"[majr] OR "Health Services for the Aged"[majr] OR "Senior Centers"[majr] OR older person*[ti] OR old person*[ti] OR older patient*[ti] OR old patient*[ti] OR "older women"[ti] OR "old women"[ti] OR "older men"[ti] OR "old men"[ti] OR old adult*[ti] OR older adult*[ti] OR "Older individual"[ti] OR "Older individuals"[ti] OR "old people"[ti] OR "older people"[ti] OR "Oldest Old"[ti] OR "Nonagenarians"[ti] OR "Nonagenarian"[ti] OR "Octogenarians"[ti] OR "Octogenarian"[ti] OR "Centenarians"[ti] OR "Centenarian"[ti] OR "septuagenarian"[ti] OR "septuagenarians"[ti] OR "Aging"[majr] OR "aging"[ti] OR "ageing"[ti] OR "older population"[ti] OR "aging population"[ti] OR "aging population"[ti] OR geront*[ti] OR "old-aged"[ti] OR "old-age"[ti] OR "old aged"[ti] OR "old age"[ti]) AND ("accelerometers"[tw] OR "accelerometer"[tw] OR "accelerometry"[tw] OR acceleromet*[tw] OR "Telemedicine"[mesh] OR "web portals"[tw] OR "web portal"[tw] OR web portal*[tw] OR e-consult*[tw] OR econsult*[tw] OR telemed*[tw] OR "ehealth"[tw] OR "e-health"[tw] OR "mhealth"[tw] OR "m-health"[tw] OR "mobile health"[tw] OR "telehealth"[tw] OR "tele-health"[tw] OR "tele health"[tw] OR **electronic communication*[ti]** OR "remote consultation"[tw] OR "remote health care"[tw] OR "remote healthcare"[tw] OR "remote care"[tw] OR "remote monitoring"[tw] OR "teleconsultation"[tw] OR teleconsult*[tw] OR **mobile*[ti] OR "webbased"[ti] OR "web-based"[ti] OR "Electronic Mail"[majr] OR "Electronic Mail"[ti] OR e-mail*[ti] OR email*[ti]** OR "Mobile Applications"[Mesh] OR "mobile apps"[tw] OR "mobile app"[tw] OR webapp*[tw] OR "SMS"[tw] OR "Cell Phones"[tw] OR "Smartphone"[tw] OR "Text Messaging"[tw] OR "Cell Phone"[mesh] OR "Cell Phone"[tw] OR "Smartphones"[tw] OR iphon*[tw] OR text messag*[tw] OR "texting"[tw] OR **"mobile"[ti]** OR "cellular phone"[tw] OR "cellular phones"[tw] OR "smart phone"[tw] OR "telemedicine"[tw] OR "tele-care"[tw] OR "telecare"[tw] OR "tele-monitoring"[tw] OR "telemonitoring"[tw] OR **"website"[ti] OR "websites"[ti]** OR "personal digital assistant"[tw] OR "computer-assisted instruction"[tw] OR "ipad"[tw] OR ipad*[tw] OR "telenursing"[tw] OR telenurs*[tw] OR "virtual community"[tw] **OR "webpage"[ti] OR "webpages"[ti]** OR "web application"[tw] OR "web applications"[tw] OR "short message service"[tw] OR **"Internet"[majr] OR "internet"[ti] OR "online"[ti] OR "digital"[ti] OR digital*[ti]** OR "Reminder Systems"[mesh] OR "Reminder Systems"[tw] OR "Reminder System"[tw] OR "Reminder Device"[tw] OR "Reminder Devices"[tw] OR "reminder messages"[tw] OR "reminder message"[tw] OR **"web"[ti]** OR "Virtual Reality"[mesh] OR "Virtual Reality"[tw] OR "smart technology"[tw] OR smart technol*[tw] OR "wearable technology"[tw] OR "wearable technologies"[tw] OR "telerehabilitation"[tw] OR

"Therapy, computer-assisted"[mesh:noexp] OR "computer-assisted therapy"[tw] OR "computer assisted therapy"[tw] OR "online therapy"[tw] OR "Computer Mediated Communication"[tw] OR "Computer Mediated Communications"[tw] OR **"remote"[ti] OR "on-line"[ti] OR "on line"[ti]** OR "wearable devices"[tw] OR "wearable device"[tw] OR "healthsensor"[tw] OR "healthsensors"[tw] OR "health sensor"[tw] OR "health sensors"[tw] OR "Robotics"[mesh] OR "robotics"[tw] OR robot*[tw] OR "exergame"[tw] OR "exergames"[tw] OR exergam*[tw] OR "Video Games"[mesh] OR "app-based"[tw] OR "wearable app"[tw] OR "wearable apps"[tw] OR "wearables"[tw] OR "Nintendo"[tw] OR "Wii"[tw] OR "gaming console"[tw] OR "gaming consoles"[tw])) **OR** (("Rehabilitation"[mesh] OR "rehabilitation"[subheading] OR rehab*[tw] OR "Rehabilitation Nursing"[mesh] OR "Rehabilitation Centers"[mesh] OR "Activities of Daily Living"[tw] OR "Animal Assisted Therapy"[tw] OR "Equine-Assisted Therapy"[tw] OR "Art Therapy"[tw] OR "Bibliotherapy"[tw] OR "Correction of Hearing Impairment"[tw] OR "Total Communication Methods"[tw] OR "Lipreading"[tw] OR "Manual Communication"[tw] OR "Dance Therapy"[tw] OR "Early Ambulation"[tw] OR "Exercise Therapy"[tw] OR "Endurance Training"[tw] OR "Continuous Passive Motion Therapy"[tw] OR "Muscle Stretching"[tw] OR "Plyometric Exercise"[tw] OR "Resistance Training"[tw] OR "Music Therapy"[tw] OR "Occupational Therapy"[tw] OR "Recreation Therapy"[tw] OR "Language Therapy"[tw] OR "Myofunctional Therapy"[tw] OR "Speech Therapy"[tw] OR "Alaryngeal Speech"[tw] OR "Voice Training"[tw] OR "Telerehabilitation"[tw] OR "Activity of Daily Living"[tw] OR "Plyometric Exercises"[tw] OR "Exercise"[mesh] OR "exercise"[tw] OR "exercises"[tw] OR "physical activity"[tw]) AND ("Aged"[mesh] OR "elderly"[ti] OR "elder"[ti] OR "elders"[ti] OR geriatr*[ti] OR "Homes for the Aged"[majr] OR "Health Services for the Aged"[majr] OR "Senior Centers"[majr] OR older person*[ti] OR old person*[ti] OR older patient*[ti] OR old patient*[ti] OR "older women"[ti] OR "old women"[ti] OR "older men"[ti] OR "old men"[ti] OR old adult*[ti] OR older adult*[ti] OR "Older individual"[ti] OR "Older individuals"[ti] OR "old people"[ti] OR "older people"[ti] OR "Oldest Old"[ti] OR "Nonagenarians"[ti] OR "Nonagenarian"[ti] OR "Octogenarians"[ti] OR "Octogenarian"[ti] OR "Centenarians"[ti] OR "Centenarian"[ti] OR "septuagenarian"[ti] OR "septuagenarians"[ti] OR "Aging"[majr] OR "aging"[ti] OR "ageing"[ti] OR "older population"[ti] OR "aging population"[ti] OR "aging population"[ti] OR geront*[ti] OR "old-aged"[ti] OR "old-age"[ti] OR "old aged"[ti] OR "old age"[ti]) AND ("accelerometers"[ti] OR "accelerometer"[ti] OR "accelerometry"[ti] OR acceleromet*[ti] OR "Telemedicine"[majr] OR "web portals"[ti] OR "web portal"[ti] OR web portal*[ti] OR e-consult*[ti] OR econsult*[ti] OR telemed*[ti] OR "ehealth"[ti] OR "e-health"[ti] OR "mhealth"[ti] OR "m-health"[ti] OR "mobile health"[ti] OR "telehealth"[ti] OR "tele-health"[ti] OR "tele health"[ti] OR **electronic communication*[ti]** OR "remote consultation"[ti] OR "remote health care"[ti] OR "remote healthcare"[ti] OR "remote care"[ti] OR "remote monitoring"[ti] OR "teleconsultation"[ti] OR teleconsult*[ti] OR **mobile*[ti] OR "webbased"[ti] OR "web-based"[ti] OR "Electronic Mail"[majr] OR "Electronic Mail"[ti] OR e-mail*[ti] OR email*[ti]** OR "Mobile Applications"[majr] OR "mobile apps"[ti] OR "mobile app"[ti] OR webapp*[ti] OR "SMS"[ti] OR "Cell Phones"[ti] OR "Smartphone"[ti] OR "Text Messaging"[ti] OR "Cell Phone"[majr] OR "Cell Phone"[ti] OR "Smartphones"[ti] OR iphon*[ti] OR text messag*[ti] OR "texting"[ti] OR

"mobile"[ti] OR "cellular phone"[ti] OR "cellular phones"[ti] OR "smart phone"[ti] OR "telemedicine"[ti] OR "tele-care"[ti] OR "telecare"[ti] OR "tele-monitoring"[ti] OR "telemonitoring"[ti] OR **"website"[ti] OR "websites"[ti]** OR "personal digital assistant"[ti] OR "computer-assisted instruction"[ti] OR "ipad"[ti] OR ipad*[ti] OR "telenursing"[ti] OR telenurs*[ti] OR "virtual community"[ti] **OR "webpage"[ti] OR "webpages"[ti]** OR "web application"[ti] OR "web applications"[ti] OR "short message service"[ti] OR **"Internet"[majr] OR "internet"[ti] OR "online"[ti] OR "digital"[ti] OR digital*[ti]** OR "Reminder Systems"[majr] OR "Reminder Systems"[ti] OR "Reminder System"[ti] OR "Reminder Device"[ti] OR "Reminder Devices"[ti] OR "reminder messages"[ti] OR "reminder message"[ti] OR **"web"[ti]** OR "Virtual Reality"[majr] OR "Virtual Reality"[ti] OR "smart technology"[ti] OR smart technol*[ti] OR "wearable technology"[ti] OR "wearable technologies"[ti] OR "telerehabilitation"[ti] OR "Therapy, computer-assisted"[majr:noexp] OR "computer-assisted therapy"[ti] OR "computer assisted therapy"[ti] OR "online therapy"[ti] OR "Computer Mediated Communication"[ti] OR "Computer Mediated Communications"[ti] OR **"remote"[ti] OR "on-line"[ti] OR "on line"[ti]** OR "wearable devices"[ti] OR "wearable device"[ti] OR "healthsensor"[ti] OR "healthsensors"[ti] OR "health sensor"[ti] OR "health sensors"[ti] OR "Robotics"[majr] OR "robotics"[ti] OR robot*[ti] OR "exergame"[ti] OR "exergames"[ti] OR exergam*[ti] OR "Video Games"[majr] OR "app-based"[ti] OR "wearable app"[ti] OR "wearable apps"[ti] OR "wearables"[ti] OR "Nintendo"[ti] OR "Wii"[ti] OR "gaming console"[ti] OR "gaming consoles"[ti]))**)** AND ("usability"[tw] OR "Meaningful Use"[Mesh] OR "Meaningful Use"[tw] OR "User experience"[tw] OR "User experiences"[tw] OR "usefulness"[tw] OR "Attitude to Computers"[Mesh] OR "use"[ti] OR "user"[ti] OR "users"[ti] OR "user interaction"[tw] OR "user interactions"[tw] OR useful*[tw] OR useless*[tw]))

(3) Een nieuwe zoekstring met een omschrijving van de term feasibility. Ook weer met de nadruk op alle vier de componenten

("feasibility"[tw] OR "feasible"[tw] OR "Feasibility Studies"[Mesh]) AND (("Rehabilitation"[majr] OR rehab*[ti] OR "Rehabilitation Nursing"[majr] OR "Rehabilitation Centers"[majr] OR "Activities of Daily Living"[ti] OR "Animal Assisted Therapy"[ti] OR "Equine-Assisted Therapy"[ti] OR "Art Therapy"[ti] OR "Bibliotherapy"[ti] OR "Correction of Hearing Impairment"[ti] OR "Total Communication Methods"[ti] OR "Lipreading"[ti] OR "Manual Communication"[ti] OR "Dance Therapy"[ti] OR "Early Ambulation"[ti] OR "Exercise Therapy"[ti] OR "Endurance Training"[ti] OR "Continuous Passive Motion Therapy"[ti] OR "Muscle Stretching"[ti] OR "Plyometric Exercise"[ti] OR "Resistance Training"[ti] OR "Music Therapy"[ti] OR "Occupational Therapy"[ti] OR "Recreation Therapy"[ti] OR "Language Therapy"[ti] OR "Myofunctional Therapy"[ti] OR "Speech Therapy"[ti] OR "Alaryngeal Speech"[ti] OR "Voice Training"[ti] OR "Telerehabilitation"[ti] OR "Activity of Daily Living"[ti] OR "Plyometric Exercises"[ti] OR "Exercise"[majr] OR "exercise"[ti] OR "exercises"[ti] OR "physical activity"[ti]) AND ("Aged"[mesh] OR "elderly"[ti] OR "elder"[ti] OR "elders"[ti] OR geriatr*[ti] OR "Homes for the Aged"[majr] OR "Health Services for the Aged"[majr] OR "Senior Centers"[majr] OR older person*[ti] OR old person*[ti] OR older patient*[ti] OR old patient*[ti] OR "older women"[ti] OR "old

women"[ti] OR "older men"[ti] OR "old men"[ti] OR old adult*[ti] OR older adult*[ti] OR "Older individual"[ti] OR "Older individuals"[ti] OR "old people"[ti] OR "older people"[ti] OR "Oldest Old"[ti] OR "Nonagenarians"[ti] OR "Nonagenarian"[ti] OR "Octogenarians"[ti] OR "Octogenarian"[ti] OR "Centenarians"[ti] OR "Centenarian"[ti] OR "septuagenarian"[ti] OR "septuagenarians"[ti] OR "Aging"[majr] OR "aging"[ti] OR "ageing"[ti] OR "older population"[ti] OR "aging population"[ti] OR "aging population"[ti] OR geront*[ti] OR "old-aged"[ti] OR "old-age"[ti] OR "old aged"[ti] OR "old age"[ti]) AND ("accelerometers"[ti] OR "accelerometer"[ti] OR "accelerometry"[ti] OR acceleromet*[ti] OR "Telemedicine"[majr] OR "web portals"[ti] OR "web portal"[ti] OR web portal*[ti] OR e-consult*[ti] OR econsult*[ti] OR telemed*[ti] OR "ehealth"[ti] OR "e-health"[ti] OR "mhealth"[ti] OR "m-health"[ti] OR "mobile health"[ti] OR "telehealth"[ti] OR "tele-health"[ti] OR "tele health"[ti] OR **electronic communication*[ti]** OR "remote consultation"[ti] OR "remote health care"[ti] OR "remote healthcare"[ti] OR "remote care"[ti] OR "remote monitoring"[ti] OR "teleconsultation"[ti] OR teleconsult*[ti] OR **mobile*[ti] OR "webbased"[ti] OR "web-based"[ti] OR "Electronic Mail"[majr] OR "Electronic Mail"[ti] OR e-mail*[ti] OR email*[ti]** OR "mobile apps"[ti] OR "mobile app"[ti] OR webapp*[ti] OR "SMS"[ti] OR "Cell Phones"[ti] OR "Smartphone"[ti] OR "Text Messaging"[ti] OR "Cell Phone"[majr] OR "Cell Phone"[ti] OR "Smartphones"[ti] OR iphon*[ti] OR text messag*[ti] OR "texting"[ti] OR **"mobile"[ti]** OR "cellular phone"[ti] OR "cellular phones"[ti] OR "smart phone"[ti] OR "telemedicine"[ti] OR "tele-care"[ti] OR "telecare"[ti] OR "tele-monitoring"[ti] OR "telemonitoring"[ti] OR **"website"[ti] OR "websites"[ti]** OR "personal digital assistant"[ti] OR "computer-assisted instruction"[ti] OR "ipad"[ti] OR ipad*[ti] OR "telenursing"[ti] OR telenurs*[ti] OR "virtual community"[ti] **OR "webpage"[ti] OR "webpages"[ti]** OR "web application"[ti] OR "web applications"[ti] OR "short message service"[ti] OR **"Internet"[majr] OR "internet"[ti] OR "online"[ti] OR "digital"[ti] OR digital*[ti]** OR "Reminder Systems"[majr] OR "Reminder Systems"[ti] OR "Reminder System"[ti] OR "Reminder Device"[ti] OR "Reminder Devices"[ti] OR "reminder messages"[ti] OR "reminder message"[ti] OR **"web"[ti]** OR "Virtual Reality"[majr] OR "Virtual Reality"[ti] OR "smart technology"[ti] OR smart technol*[ti] OR "wearable technology"[ti] OR "wearable technologies"[ti] OR "telerehabilitation"[ti] OR "Therapy, computer-assisted"[majr:noexp] OR "computer-assisted therapy"[ti] OR "computer assisted therapy"[ti] OR "online therapy"[ti] OR "Computer Mediated Communication"[ti] OR "Computer Mediated Communications"[ti] OR **"remote"[ti] OR "on-line"[ti] OR "on line"[ti]** OR "wearable devices"[ti] OR "wearable device"[ti] OR "healthsensor"[ti] OR "healthsensors"[ti] OR "health sensor"[ti] OR "health sensors"[ti] OR "Robotics"[majr] OR "robotics"[ti] OR robot*[ti] OR "exergame"[ti] OR "exergames"[ti] OR exergam*[ti] OR "Video Games"[majr] OR "app-based"[ti] OR "wearable app"[ti] OR "wearable apps"[ti] OR "wearables"[ti] OR "Nintendo"[ti] OR "Wii"[ti] OR "gaming console"[ti] OR "gaming consoles"[ti]))

10-4-2019

B. Nadruk op Revalidatie en/óf E-health: : 6.099 refs d.d. 9-4-2019

((("Rehabilitation"[majr] OR rehab*[ti] OR "Rehabilitation Nursing"[majr] OR "Rehabilitation Centers"[majr] OR "Activities of Daily Living"[ti] OR "Animal Assisted Therapy"[ti] OR "Equine-Assisted Therapy"[ti] OR "Art Therapy"[ti] OR "Bibliotherapy"[ti] OR "Correction of Hearing Impairment"[ti] OR "Total Communication Methods"[ti] OR "Lipreading"[ti] OR "Manual Communication"[ti] OR "Dance Therapy"[ti] OR "Early Ambulation"[ti] OR "Exercise Therapy"[ti] OR "Endurance Training"[ti] OR "Continuous Passive Motion Therapy"[ti] OR "Muscle Stretching"[ti] OR "Plyometric Exercise"[ti] OR "Resistance Training"[ti] OR "Music Therapy"[ti] OR "Occupational Therapy"[ti] OR "Recreation Therapy"[ti] OR "Language Therapy"[ti] OR "Myofunctional Therapy"[ti] OR "Speech Therapy"[ti] OR "Alaryngeal Speech"[ti] OR "Voice Training"[ti] OR "Telerehabilitation"[ti] OR "Activity of Daily Living"[ti] OR "Plyometric Exercises"[ti] OR "Exercise"[majr] OR "exercise"[ti] OR "exercises"[ti] OR "physical activity"[ti]) AND ("Aged"[mesh] OR "elderly"[ti] OR "elder"[ti] OR "elders"[ti] OR geriatr*[ti] OR "Homes for the Aged"[majr] OR "Health Services for the Aged"[majr] OR "Senior Centers"[majr] OR older person*[ti] OR old person*[ti] OR older patient*[ti] OR old patient*[ti] OR "older women"[ti] OR "old women"[ti] OR "older men"[ti] OR "old men"[ti] OR old adult*[ti] OR older adult*[ti] OR "Older individual"[ti] OR "Older individuals"[ti] OR "old people"[ti] OR "older people"[ti] OR "Oldest Old"[ti] OR "Nonagenarians"[ti] OR "Nonagenarian"[ti] OR "Octogenarians"[ti] OR "Octogenarian"[ti] OR "Centenarians"[ti] OR "Centenarian"[ti] OR "septuagenarian"[ti] OR "septuagenarians"[ti] OR "Aging"[majr] OR "aging"[ti] OR "ageing"[ti] OR "older population"[ti] OR "aging population"[ti] OR "aging population"[ti] OR geront*[ti] OR "old-aged"[ti] OR "old-age"[ti] OR "old aged"[ti] OR "old age"[ti]) AND ("accelerometers"[tw] OR "accelerometer"[tw] OR "accelerometry"[tw] OR acceleromet*[tw] OR "Telemedicine"[mesh] OR "web portals"[tw] OR "web portal"[tw] OR web portal*[tw] OR e-consult*[tw] OR econsult*[tw] OR telemed*[tw] OR "ehealth"[tw] OR "e-health"[tw] OR "mhealth"[tw] OR "m-health"[tw] OR "mobile health"[tw] OR "telehealth"[tw] OR "tele-health"[tw] OR "tele health"[tw] OR **electronic communication*[ti]** OR "remote consultation"[tw] OR "remote health care"[tw] OR "remote healthcare"[tw] OR "remote care"[tw] OR "remote monitoring"[tw] OR "teleconsultation"[tw] OR teleconsult*[tw] OR **mobile*[ti] OR "webbased"[ti] OR "web-based"[ti] OR "Electronic Mail"[majr] OR "Electronic Mail"[ti] OR e-mail*[ti] OR email*[ti]** OR "Mobile Applications"[Mesh] OR "mobile apps"[tw] OR "mobile app"[tw] OR webapp*[tw] OR "SMS"[tw] OR "Cell Phones"[tw] OR "Smartphone"[tw] OR "Text Messaging"[tw] OR "Cell Phone"[mesh] OR "Cell Phone"[tw] OR "Smartphones"[tw] OR iphon*[tw] OR text messag*[tw] OR "texting"[tw] OR **"mobile"[ti]** OR "cellular phone"[tw] OR "cellular phones"[tw] OR "smart phone"[tw] OR "telemedicine"[tw] OR "tele-care"[tw] OR "telecare"[tw] OR "tele-monitoring"[tw] OR "telemonitoring"[tw] OR **"website"[ti] OR "websites"[ti]** OR "personal digital assistant"[tw] OR "computer-assisted instruction"[tw] OR "ipad"[tw] OR ipad*[tw] OR "telenursing"[tw] OR telenurs*[tw] OR "virtual community"[tw] **OR "webpage"[ti] OR "webpages"[ti]** OR "web application"[tw] OR "web applications"[tw] OR "short message service"[tw] OR **"Internet"[majr] OR "internet"[ti] OR "online"[ti] OR "digital"[ti] OR digital*[ti]** OR "Reminder Systems"[mesh] OR "Reminder Systems"[tw] OR "Reminder System"[tw] OR

"Reminder Device"[tw] OR "Reminder Devices"[tw] OR "reminder messages"[tw] OR "reminder message"[tw] OR **"web"[ti]** OR "Virtual Reality"[mesh] OR "Virtual Reality"[tw] OR "smart technology"[tw] OR smart technol*[tw] OR "wearable technology"[tw] OR "wearable technologies"[tw] OR "telerehabilitation"[tw] OR "Therapy, computer-assisted"[mesh:noexp] OR "computer-assisted therapy"[tw] OR "computer assisted therapy"[tw] OR "online therapy"[tw] OR "Computer Mediated Communication"[tw] OR "Computer Mediated Communications"[tw] OR **"remote"[ti] OR "on-line"[ti] OR "on line"[ti]** OR "wearable devices"[tw] OR "wearable device"[tw] OR "healthsensor"[tw] OR "healthsensors"[tw] OR "health sensor"[tw] OR "health sensors"[tw] OR "Robotics"[mesh] OR "robotics"[tw] OR robot*[tw] OR "exergame"[tw] OR "exergames"[tw] OR exergam*[tw] OR "Video Games"[mesh] OR "app-based"[tw] OR "wearable app"[tw] OR "wearable apps"[tw] OR "wearables"[tw] OR "Nintendo"[tw] OR "Wii"[tw] OR "gaming console"[tw] OR "gaming consoles"[tw])) **OR** (("Rehabilitation"[mesh] OR "rehabilitation"[subheading] OR rehab*[tw] OR "Rehabilitation Nursing"[mesh] OR "Rehabilitation Centers"[mesh] OR "Activities of Daily Living"[tw] OR "Animal Assisted Therapy"[tw] OR "Equine-Assisted Therapy"[tw] OR "Art Therapy"[tw] OR "Bibliotherapy"[tw] OR "Correction of Hearing Impairment"[tw] OR "Total Communication Methods"[tw] OR "Lipreading"[tw] OR "Manual Communication"[tw] OR "Dance Therapy"[tw] OR "Early Ambulation"[tw] OR "Exercise Therapy"[tw] OR "Endurance Training"[tw] OR "Continuous Passive Motion Therapy"[tw] OR "Muscle Stretching"[tw] OR "Plyometric Exercise"[tw] OR "Resistance Training"[tw] OR "Music Therapy"[tw] OR "Occupational Therapy"[tw] OR "Recreation Therapy"[tw] OR "Language Therapy"[tw] OR "Myofunctional Therapy"[tw] OR "Speech Therapy"[tw] OR "Alaryngeal Speech"[tw] OR "Voice Training"[tw] OR "Telerehabilitation"[tw] OR "Activity of Daily Living"[tw] OR "Plyometric Exercises"[tw] OR "Exercise"[mesh] OR "exercise"[tw] OR "exercises"[tw] OR "physical activity"[tw]) AND ("Aged"[mesh] OR "elderly"[ti] OR "elder"[ti] OR "elders"[ti] OR geriatr*[ti] OR "Homes for the Aged"[majr] OR "Health Services for the Aged"[majr] OR "Senior Centers"[majr] OR older person*[ti] OR old person*[ti] OR older patient*[ti] OR old patient*[ti] OR "older women"[ti] OR "old women"[ti] OR "older men"[ti] OR "old men"[ti] OR old adult*[ti] OR older adult*[ti] OR "Older individual"[ti] OR "Older individuals"[ti] OR "old people"[ti] OR "older people"[ti] OR "Oldest Old"[ti] OR "Nonagenarians"[ti] OR "Nonagenarian"[ti] OR "Octogenarians"[ti] OR "Octogenarian"[ti] OR "Centenarians"[ti] OR "Centenarian"[ti] OR "septuagenarian"[ti] OR "septuagenarians"[ti] OR "Aging"[majr] OR "aging"[ti] OR "ageing"[ti] OR "older population"[ti] OR "aging population"[ti] OR "aging population"[ti] OR geront*[ti] OR "old-aged"[ti] OR "old-age"[ti] OR "old aged"[ti] OR "old age"[ti]) AND ("accelerometers"[ti] OR "accelerometer"[ti] OR "accelerometry"[ti] OR acceleromet*[ti] OR "Telemedicine"[majr] OR "web portals"[ti] OR "web portal"[ti] OR web portal*[ti] OR e-consult*[ti] OR econsult*[ti] OR telemed*[ti] OR "ehealth"[ti] OR "e-health"[ti] OR "mhealth"[ti] OR "m-health"[ti] OR "mobile health"[ti] OR "telehealth"[ti] OR "tele-health"[ti] OR "tele health"[ti] OR **electronic communication*[ti]** OR "remote consultation"[ti] OR "remote health care"[ti] OR "remote healthcare"[ti] OR "remote care"[ti] OR "remote monitoring"[ti] OR "teleconsultation"[ti] OR teleconsult*[ti] OR **mobile*[ti] OR "webbased"[ti] OR "web-based"[ti] OR "Electronic Mail"[majr] OR "Electronic Mail"[ti] OR e-**

mail*[ti] OR email*[ti] OR "Mobile Applications"[majr] OR "mobile apps"[ti] OR "mobile app"[ti] OR webapp*[ti] OR "SMS"[ti] OR "Cell Phones"[ti] OR "Smartphone"[ti] OR "Text Messaging"[ti] OR "Cell Phone"[majr] OR "Cell Phone"[ti] OR "Smartphones"[ti] OR iphon*[ti] OR text messag*[ti] OR "texting"[ti] OR **"mobile"[ti]** OR "cellular phone"[ti] OR "cellular phones"[ti] OR "smart phone"[ti] OR "telemedicine"[ti] OR "tele-care"[ti] OR "telecare"[ti] OR "tele-monitoring"[ti] OR "telemonitoring"[ti] OR **"website"[ti] OR "websites"[ti]** OR "personal digital assistant"[ti] OR "computer-assisted instruction"[ti] OR "ipad"[ti] OR ipad*[ti] OR "telenursing"[ti] OR telenurs*[ti] OR "virtual community"[ti] **OR "webpage"[ti] OR "webpages"[ti]** OR "web application"[ti] OR "web applications"[ti] OR "short message service"[ti] OR **"Internet"[majr] OR "internet"[ti] OR "online"[ti] OR "digital"[ti] OR digital*[ti]** OR "Reminder Systems"[majr] OR "Reminder Systems"[ti] OR "Reminder System"[ti] OR "Reminder Device"[ti] OR "Reminder Devices"[ti] OR "reminder messages"[ti] OR "reminder message"[ti] OR **"web"[ti]** OR "Virtual Reality"[majr] OR "Virtual Reality"[ti] OR "smart technology"[ti] OR smart technol*[ti] OR "wearable technology"[ti] OR "wearable technologies"[ti] OR "telerehabilitation"[ti] OR "Therapy, computer-assisted"[majr:noexp] OR "computer-assisted therapy"[ti] OR "computer assisted therapy"[ti] OR "online therapy"[ti] OR "Computer Mediated Communication"[ti] OR "Computer Mediated Communications"[ti] OR **"remote"[ti] OR "on-line"[ti] OR "on line"[ti]** OR "wearable devices"[ti] OR "wearable device"[ti] OR "healthsensor"[ti] OR "healthsensors"[ti] OR "health sensor"[ti] OR "health sensors"[ti] OR "Robotics"[majr] OR "robotics"[ti] OR robot*[ti] OR "exergame"[ti] OR "exergames"[ti] OR exergam*[ti] OR "Video Games"[majr] OR "app-based"[ti] OR "wearable app"[ti] OR "wearable apps"[ti] OR "wearables"[ti] OR "Nintendo"[ti] OR "Wii"[ti] OR "gaming console"[ti] OR "gaming consoles"[ti])))

B.2 - Nadruk op Revalidatie en/óf E-health – usability: 692 refs d.d. 9-4-2019

((("Rehabilitation"[majr] OR rehab*[ti] OR "Rehabilitation Nursing"[majr] OR "Rehabilitation Centers"[majr] OR "Activities of Daily Living"[ti] OR "Animal Assisted Therapy"[ti] OR "Equine-Assisted Therapy"[ti] OR "Art Therapy"[ti] OR "Bibliotherapy"[ti] OR "Correction of Hearing Impairment"[ti] OR "Total Communication Methods"[ti] OR "Lipreading"[ti] OR "Manual Communication"[ti] OR "Dance Therapy"[ti] OR "Early Ambulation"[ti] OR "Exercise Therapy"[ti] OR "Endurance Training"[ti] OR "Continuous Passive Motion Therapy"[ti] OR "Muscle Stretching"[ti] OR "Plyometric Exercise"[ti] OR "Resistance Training"[ti] OR "Music Therapy"[ti] OR "Occupational Therapy"[ti] OR "Recreation Therapy"[ti] OR "Language Therapy"[ti] OR "Myofunctional Therapy"[ti] OR "Speech Therapy"[ti] OR "Alaryngeal Speech"[ti] OR "Voice Training"[ti] OR "Telerehabilitation"[ti] OR "Activity of Daily Living"[ti] OR "Plyometric Exercises"[ti] OR "Exercise"[majr] OR "exercise"[ti] OR "exercises"[ti] OR "physical activity"[ti]) AND ("Aged"[mesh] OR "elderly"[ti] OR "elder"[ti] OR "elders"[ti] OR geriatr*[ti] OR "Homes for the Aged"[majr] OR "Health Services for the Aged"[majr] OR "Senior Centers"[majr] OR older person*[ti] OR old person*[ti] OR older patient*[ti] OR old patient*[ti] OR "older women"[ti] OR "old women"[ti] OR "older men"[ti] OR "old men"[ti] OR old adult*[ti] OR older adult*[ti] OR "Older individual"[ti] OR "Older individuals"[ti] OR "old people"[ti] OR "older

people"[ti] OR "Oldest Old"[ti] OR "Nonagenarians"[ti] OR "Nonagenarian"[ti] OR "Octogenarians"[ti] OR "Octogenarian"[ti] OR "Centenarians"[ti] OR "Centenarian"[ti] OR "septuagenarian"[ti] OR "septuagenarians"[ti] OR "Aging"[majr] OR "aging"[ti] OR "ageing"[ti] OR "older population"[ti] OR "aging population"[ti] OR "aging population"[ti] OR geront*[ti] OR "old-aged"[ti] OR "old-age"[ti] OR "old aged"[ti] OR "old age"[ti]) AND ("accelerometers"[tw] OR "accelerometer"[tw] OR "accelerometry"[tw] OR acceleromet*[tw] OR "Telemedicine"[mesh] OR "web portals"[tw] OR "web portal"[tw] OR web portal*[tw] OR e-consult*[tw] OR econsult*[tw] OR telemed*[tw] OR "ehealth"[tw] OR "e-health"[tw] OR "mhealth"[tw] OR "m-health"[tw] OR "mobile health"[tw] OR "telehealth"[tw] OR "tele-health"[tw] OR "tele health"[tw] OR **electronic communication*[ti]** OR "remote consultation"[tw] OR "remote health care"[tw] OR "remote healthcare"[tw] OR "remote care"[tw] OR "remote monitoring"[tw] OR "teleconsultation"[tw] OR teleconsult*[tw] OR **mobile*[ti] OR "webbased"[ti] OR "web-based"[ti] OR "Electronic Mail"[majr] OR "Electronic Mail"[ti] OR e-mail*[ti] OR email*[ti]** OR "Mobile Applications"[Mesh] OR "mobile apps"[tw] OR "mobile app"[tw] OR webapp*[tw] OR "SMS"[tw] OR "Cell Phones"[tw] OR "Smartphone"[tw] OR "Text Messaging"[tw] OR "Cell Phone"[mesh] OR "Cell Phone"[tw] OR "Smartphones"[tw] OR iphon*[tw] OR text messag*[tw] OR "texting"[tw] OR **"mobile"[ti]** OR "cellular phone"[tw] OR "cellular phones"[tw] OR "smart phone"[tw] OR "telemedicine"[tw] OR "tele-care"[tw] OR "telecare"[tw] OR "tele-monitoring"[tw] OR "telemonitoring"[tw] OR **"website"[ti] OR "websites"[ti]** OR "personal digital assistant"[tw] OR "computer-assisted instruction"[tw] OR "ipad"[tw] OR ipad*[tw] OR "telenursing"[tw] OR telenurs*[tw] OR "virtual community"[tw] **OR "webpage"[ti] OR "webpages"[ti]** OR "web application"[tw] OR "web applications"[tw] OR "short message service"[tw] OR **"Internet"[majr] OR "internet"[ti] OR "online"[ti] OR "digital"[ti] OR digital*[ti]** OR "Reminder Systems"[mesh] OR "Reminder Systems"[tw] OR "Reminder System"[tw] OR "Reminder Device"[tw] OR "Reminder Devices"[tw] OR "reminder messages"[tw] OR "reminder message"[tw] OR **"web"[ti]** OR "Virtual Reality"[mesh] OR "Virtual Reality"[tw] OR "smart technology"[tw] OR smart technol*[tw] OR "wearable technology"[tw] OR "wearable technologies"[tw] OR "telerehabilitation"[tw] OR "Therapy, computer-assisted"[mesh:noexp] OR "computer-assisted therapy"[tw] OR "computer assisted therapy"[tw] OR "online therapy"[tw] OR "Computer Mediated Communication"[tw] OR "Computer Mediated Communications"[tw] OR **"remote"[ti] OR "on-line"[ti] OR "on line"[ti]** OR "wearable devices"[tw] OR "wearable device"[tw] OR "healthsensor"[tw] OR "healthsensors"[tw] OR "health sensor"[tw] OR "health sensors"[tw] OR "Robotics"[mesh] OR "robotics"[tw] OR robot*[tw] OR "exergame"[tw] OR "exergames"[tw] OR exergam*[tw] OR "Video Games"[mesh] OR "app-based"[tw] OR "wearable app"[tw] OR "wearable apps"[tw] OR "wearables"[tw] OR "Nintendo"[tw] OR "Wii"[tw] OR "gaming console"[tw] OR "gaming consoles"[tw])) **OR** (("Rehabilitation"[mesh] OR "rehabilitation"[subheading] OR rehab*[tw] OR "Rehabilitation Nursing"[mesh] OR "Rehabilitation Centers"[mesh] OR "Activities of Daily Living"[tw] OR "Animal Assisted Therapy"[tw] OR "Equine-Assisted Therapy"[tw] OR "Art Therapy"[tw] OR "Bibliotherapy"[tw] OR "Correction of Hearing Impairment"[tw] OR "Total Communication Methods"[tw] OR "Lipreading"[tw] OR "Manual Communication"[tw] OR "Dance Therapy"[tw] OR "Early Ambulation"[tw]

OR "Exercise Therapy"[tw] OR "Endurance Training"[tw] OR "Continuous Passive Motion Therapy"[tw] OR "Muscle Stretching"[tw] OR "Plyometric Exercise"[tw] OR "Resistance Training"[tw] OR "Music Therapy"[tw] OR "Occupational Therapy"[tw] OR "Recreation Therapy"[tw] OR "Language Therapy"[tw] OR "Myofunctional Therapy"[tw] OR "Speech Therapy"[tw] OR "Alaryngeal Speech"[tw] OR "Voice Training"[tw] OR "Telerehabilitation"[tw] OR "Activity of Daily Living"[tw] OR "Plyometric Exercises"[tw] OR "Exercise"[mesh] OR "exercise"[tw] OR "exercises"[tw] OR "physical activity"[tw]) AND ("Aged"[mesh] OR "elderly"[ti] OR "elder"[ti] OR "elders"[ti] OR geriatr*[ti] OR "Homes for the Aged"[majr] OR "Health Services for the Aged"[majr] OR "Senior Centers"[majr] OR older person*[ti] OR old person*[ti] OR older patient*[ti] OR old patient*[ti] OR "older women"[ti] OR "old women"[ti] OR "older men"[ti] OR "old men"[ti] OR old adult*[ti] OR older adult*[ti] OR "Older individual"[ti] OR "Older individuals"[ti] OR "old people"[ti] OR "older people"[ti] OR "Oldest Old"[ti] OR "Nonagenarians"[ti] OR "Nonagenarian"[ti] OR "Octogenarians"[ti] OR "Octogenarian"[ti] OR "Centenarians"[ti] OR "Centenarian"[ti] OR "septuagenarian"[ti] OR "septuagenarians"[ti] OR "Aging"[majr] OR "aging"[ti] OR "ageing"[ti] OR "older population"[ti] OR "aging population"[ti] OR "aging population"[ti] OR geront*[ti] OR "old-aged"[ti] OR "old-age"[ti] OR "old aged"[ti] OR "old age"[ti]) AND ("accelerometers"[ti] OR "accelerometer"[ti] OR "accelerometry"[ti] OR acceleromet*[ti] OR "Telemedicine"[majr] OR "web portals"[ti] OR "web portal"[ti] OR web portal*[ti] OR e-consult*[ti] OR econsult*[ti] OR telemed*[ti] OR "ehealth"[ti] OR "e-health"[ti] OR "mhealth"[ti] OR "m-health"[ti] OR "mobile health"[ti] OR "telehealth"[ti] OR "tele-health"[ti] OR "tele health"[ti] OR **electronic communication*[ti]** OR "remote consultation"[ti] OR "remote health care"[ti] OR "remote healthcare"[ti] OR "remote care"[ti] OR "remote monitoring"[ti] OR "teleconsultation"[ti] OR teleconsult*[ti] OR **mobile*[ti] OR "webbased"[ti] OR "web-based"[ti] OR "Electronic Mail"[majr] OR "Electronic Mail"[ti] OR e-mail*[ti] OR email*[ti]** OR "Mobile Applications"[majr] OR "mobile apps"[ti] OR "mobile app"[ti] OR webapp*[ti] OR "SMS"[ti] OR "Cell Phones"[ti] OR "Smartphone"[ti] OR "Text Messaging"[ti] OR "Cell Phone"[majr] OR "Cell Phone"[ti] OR "Smartphones"[ti] OR iphon*[ti] OR text messag*[ti] OR "texting"[ti] OR **"mobile"[ti]** OR "cellular phone"[ti] OR "cellular phones"[ti] OR "smart phone"[ti] OR "telemedicine"[ti] OR "tele-care"[ti] OR "telecare"[ti] OR "tele-monitoring"[ti] OR "telemonitoring"[ti] OR **"website"[ti] OR "websites"[ti]** OR "personal digital assistant"[ti] OR "computer-assisted instruction"[ti] OR "ipad"[ti] OR ipad*[ti] OR "telenursing"[ti] OR telenurs*[ti] OR "virtual community"[ti] **OR "webpage"[ti] OR "webpages"[ti]** OR "web application"[ti] OR "web applications"[ti] OR "short message service"[ti] OR **"Internet"[majr] OR "internet"[ti] OR "online"[ti] OR "digital"[ti] OR digital*[ti]** OR "Reminder Systems"[majr] OR "Reminder Systems"[ti] OR "Reminder System"[ti] OR "Reminder Device"[ti] OR "Reminder Devices"[ti] OR "reminder messages"[ti] OR "reminder message"[ti] OR **"web"[ti]** OR "Virtual Reality"[majr] OR "Virtual Reality"[ti] OR "smart technology"[ti] OR smart technol*[ti] OR "wearable technology"[ti] OR "wearable technologies"[ti] OR "telerehabilitation"[ti] OR "Therapy, computer-assisted"[majr:noexp] OR "computer-assisted therapy"[ti] OR "computer assisted therapy"[ti] OR "online therapy"[ti] OR "Computer Mediated Communication"[ti] OR "Computer Mediated Communications"[ti] OR **"remote"[ti]**

OR "on-line"[ti] OR "on line"[ti] OR "wearable devices"[ti] OR "wearable device"[ti] OR "healthsensor"[ti] OR "healthsensors"[ti] OR "health sensor"[ti] OR "health sensors"[ti] OR "Robotics"[majr] OR "robotics"[ti] OR robot*[ti] OR "exergame"[ti] OR "exergames"[ti] OR exergam*[ti] OR "Video Games"[majr] OR "app-based"[ti] OR "wearable app"[ti] OR "wearable apps"[ti] OR "wearables"[ti] OR "Nintendo"[ti] OR "Wii"[ti] OR "gaming console"[ti] OR "gaming consoles"[ti]))) AND ("usability"[tw] OR "Meaningful Use"[Mesh] OR "Meaningful Use"[tw] OR "User experience"[tw] OR "User experiences"[tw] OR "usefulness"[tw] OR "Attitude to Computers"[Mesh] OR "use"[ti] OR "user"[ti] OR "users"[ti] OR "user interaction"[tw] OR "user interactions"[tw] OR useful*[tw] OR useless*[tw]))

A. Nadruk op drie componenten

(("Rehabilitation"[majr] OR rehab*[ti] OR "Rehabilitation Nursing"[majr] OR "Rehabilitation Centers"[majr] OR "Activities of Daily Living"[ti] OR "Animal Assisted Therapy"[ti] OR "Equine-Assisted Therapy"[ti] OR "Art Therapy"[ti] OR "Bibliotherapy"[ti] OR "Correction of Hearing Impairment"[ti] OR "Total Communication Methods"[ti] OR "Lipreading"[ti] OR "Manual Communication"[ti] OR "Dance Therapy"[ti] OR "Early Ambulation"[ti] OR "Exercise Therapy"[ti] OR "Endurance Training"[ti] OR "Continuous Passive Motion Therapy"[ti] OR "Muscle Stretching"[ti] OR "Plyometric Exercise"[ti] OR "Resistance Training"[ti] OR "Music Therapy"[ti] OR "Occupational Therapy"[ti] OR "Recreation Therapy"[ti] OR "Language Therapy"[ti] OR "Myofunctional Therapy"[ti] OR "Speech Therapy"[ti] OR "Alaryngeal Speech"[ti] OR "Voice Training"[ti] OR "Telerehabilitation"[ti] OR "Activity of Daily Living"[ti] OR "Plyometric Exercises"[ti] OR "Exercise"[majr] OR "exercise"[ti] OR "exercises"[ti] OR "physical activity"[ti]) AND ("Aged"[mesh] OR "elderly"[ti] OR "elder"[ti] OR "elders"[ti] OR geriatr*[ti] OR "Homes for the Aged"[majr] OR "Health Services for the Aged"[majr] OR "Senior Centers"[majr] OR older person*[ti] OR old person*[ti] OR older patient*[ti] OR old patient*[ti] OR "older women"[ti] OR "old women"[ti] OR "older men"[ti] OR "old men"[ti] OR old adult*[ti] OR older adult*[ti] OR "Older individual"[ti] OR "Older individuals"[ti] OR "old people"[ti] OR "older people"[ti] OR "Oldest Old"[ti] OR "Nonagenarians"[ti] OR "Nonagenarian"[ti] OR "Octogenarians"[ti] OR "Octogenarian"[ti] OR "Centenarians"[ti] OR "Centenarian"[ti] OR "septuagenarian"[ti] OR "septuagenarians"[ti] OR "Aging"[majr] OR "aging"[ti] OR "ageing"[ti] OR "older population"[ti] OR "aging population"[ti] OR "aging population"[ti] OR geront*[ti] OR "old-aged"[ti] OR "old-age"[ti] OR "old aged"[ti] OR "old age"[ti]) AND ("accelerometers"[ti] OR "accelerometer"[ti] OR "accelerometry"[ti] OR acceleromet*[ti] OR "Telemedicine"[majr] OR "web portals"[ti] OR "web portal"[ti] OR web portal*[ti] OR e-consult*[ti] OR econsult*[ti] OR telemed*[ti] OR "ehealth"[ti] OR "e-health"[ti] OR "mhealth"[ti] OR "m-health"[ti] OR "mobile health"[ti] OR "telehealth"[ti] OR "tele-health"[ti] OR "tele health"[ti] OR **electronic communication*[ti]** OR "remote consultation"[ti] OR "remote health care"[ti] OR "remote healthcare"[ti] OR "remote care"[ti] OR "remote monitoring"[ti] OR "teleconsultation"[ti] OR teleconsult*[ti] OR **mobile*[ti] OR "webbased"[ti] OR**

"web-based"[ti] OR "Electronic Mail"[majr] OR "Electronic Mail"[ti] OR e-mail*[ti] OR email*[ti] OR "mobile apps"[ti] OR "mobile app"[ti] OR webapp*[ti] OR "SMS"[ti] OR "Cell Phones"[ti] OR "Smartphone"[ti] OR "Text Messaging"[ti] OR "Cell Phone"[majr] OR "Cell Phone"[ti] OR "Smartphones"[ti] OR iphon*[ti] OR text messag*[ti] OR "texting"[ti] OR **"mobile"[ti]** OR "cellular phone"[ti] OR "cellular phones"[ti] OR "smart phone"[ti] OR "telemedicine"[ti] OR "tele-care"[ti] OR "telecare"[ti] OR "tele-monitoring"[ti] OR "telemonitoring"[ti] OR **"website"[ti] OR "websites"[ti]** OR "personal digital assistant"[ti] OR "computer-assisted instruction"[ti] OR "ipad"[ti] OR ipad*[ti] OR "telenursing"[ti] OR telenurs*[ti] OR "virtual community"[ti] **OR "webpage"[ti] OR "webpages"[ti]** OR "web application"[ti] OR "web applications"[ti] OR "short message service"[ti] OR **"Internet"[majr] OR "internet"[ti] OR "online"[ti] OR "digital"[ti] OR digital*[ti]** OR "Reminder Systems"[majr] OR "Reminder Systems"[ti] OR "Reminder System"[ti] OR "Reminder Device"[ti] OR "Reminder Devices"[ti] OR "reminder messages"[ti] OR "reminder message"[ti] OR **"web"[ti]** OR "Virtual Reality"[majr] OR "Virtual Reality"[ti] OR "smart technology"[ti] OR smart technol*[ti] OR "wearable technology"[ti] OR "wearable technologies"[ti] OR "telerehabilitation"[ti] OR "Therapy, computer-assisted"[majr:noexp] OR "computer-assisted therapy"[ti] OR "computer assisted therapy"[ti] OR "online therapy"[ti] OR "Computer Mediated Communication"[ti] OR "Computer Mediated Communications"[ti] OR **"remote"[ti] OR "on-line"[ti] OR "on line"[ti]** OR "wearable devices"[ti] OR "wearable device"[ti] OR "healthsensor"[ti] OR "healthsensors"[ti] OR "health sensor"[ti] OR "health sensors"[ti] OR "Robotics"[majr] OR "robotics"[ti] OR robot*[ti] OR "exergame"[ti] OR "exergames"[ti] OR exergam*[ti] OR "Video Games"[majr] OR "app-based"[ti] OR "wearable app"[ti] OR "wearable apps"[ti] OR "wearables"[ti] OR "Nintendo"[ti] OR "Wii"[ti] OR "gaming console"[ti] OR "gaming consoles"[ti]))

C. Breed

(("Rehabilitation"[Mesh] OR rehab*[tw] OR "rehabilitation"[Subheading] OR "Rehabilitation Nursing"[Mesh] OR "Rehabilitation Centers"[Mesh] OR "Activities of Daily Living"[tw] OR "Animal Assisted Therapy"[tw] OR "Equine-Assisted Therapy"[tw] OR "Art Therapy"[tw] OR "Bibliotherapy"[tw] OR "Correction of Hearing Impairment"[tw] OR "Total Communication Methods"[tw] OR "Lipreading"[tw] OR "Manual Communication"[tw] OR "Dance Therapy"[tw] OR "Early Ambulation"[tw] OR "Exercise Therapy"[tw] OR "Endurance Training"[tw] OR "Continuous Passive Motion Therapy"[tw] OR "Muscle Stretching"[tw] OR "Plyometric Exercise"[tw] OR "Resistance Training"[tw] OR "Music Therapy"[tw] OR "Occupational Therapy"[tw] OR "Recreation Therapy"[tw] OR "Language Therapy"[tw] OR "Myofunctional Therapy"[tw] OR "Speech Therapy"[tw] OR "Alaryngeal Speech"[tw] OR "Voice Training"[tw] OR "Telerehabilitation"[tw] OR "Activity of Daily Living"[tw] OR "Plyometric Exercises"[tw] OR "Exercise"[mesh] OR "exercise"[tw] OR "exercises"[tw] OR "physical activity"[tw]) AND ("Aged"[mesh] OR "elderly"[all fields] OR "elder"[all fields] OR "elders"[all fields] OR geriatr*[all fields] OR "Homes for the Aged"[mesh] OR "Health Services for the Aged"[mesh] OR "Senior Centers"[mesh] OR older

person*[all fields] OR old person*[all fields] OR older patient*[all fields] OR old patient*[all fields] OR "older women"[all fields] OR "old women"[all fields] OR "older men"[all fields] OR "old men"[all fields] OR old adult*[all fields] OR older adult*[all fields] OR "Older individual"[all fields] OR "Older individuals"[all fields] OR "old people"[all fields] OR "older people"[all fields] OR "Oldest Old"[all fields] OR "Nonagenarians"[all fields] OR "Nonagenarian"[all fields] OR "Octogenarians"[all fields] OR "Octogenarian"[all fields] OR "Centenarians"[all fields] OR "Centenarian"[all fields] OR "septuagenarian"[all fields] OR "septuagenarians"[all fields] OR "Aging"[mesh] OR "aging"[all fields] OR "ageing"[all fields] OR "older population"[all fields] OR "aging population"[all fields] OR "aging population"[all fields] OR geront*[all fields] OR "old-aged"[all fields] OR "old-age"[all fields] OR "old aged"[all fields] OR "old age"[all fields]) AND ("accelerometers"[tw] OR "accelerometer"[tw] OR "accelerometry"[tw] OR acceleromet*[tw] OR "Telemedicine"[mesh] OR "web portals"[tw] OR "web portal"[tw] OR web portal*[tw] OR e-consult*[tw] OR econsult*[tw] OR telemed*[tw] OR "ehealth"[tw] OR "e-health"[tw] OR "mhealth"[tw] OR "m-health"[tw] OR "mobile health"[tw] OR "telehealth"[tw] OR "tele-health"[tw] OR "tele health"[tw] OR **electronic communication*[ti]** OR "remote consultation"[tw] OR "remote health care"[tw] OR "remote healthcare"[tw] OR "remote care"[tw] OR "remote monitoring"[tw] OR "teleconsultation"[tw] OR teleconsult*[tw] OR **mobile*[ti] OR "webbased"[ti] OR "web-based"[ti] OR "Electronic Mail"[majr] OR "Electronic Mail"[ti] OR e-mail*[ti] OR email*[ti]** OR "Mobile Applications"[Mesh] OR "mobile apps"[tw] OR "mobile app"[tw] OR webapp*[tw] OR "SMS"[tw] OR "Cell Phones"[tw] OR "Smartphone"[tw] OR "Text Messaging"[tw] OR "Cell Phone"[mesh] OR "Cell Phone"[tw] OR "Smartphones"[tw] OR iphon*[tw] OR text messag*[tw] OR "texting"[tw] OR **"mobile"[ti]** OR "cellular phone"[tw] OR "cellular phones"[tw] OR "smart phone"[tw] OR "telemedicine"[tw] OR "tele-care"[tw] OR "telecare"[tw] OR "tele-monitoring"[tw] OR "telemonitoring"[tw] OR **"website"[ti] OR "websites"[ti]** OR "personal digital assistant"[tw] OR "computer-assisted instruction"[tw] OR "ipad"[tw] OR ipad*[tw] OR "telenursing"[tw] OR telenurs*[tw] OR "virtual community"[tw] **OR "webpage"[ti] OR "webpages"[ti]** OR "web application"[tw] OR "web applications"[tw] OR "short message service"[tw] OR **"Internet"[majr] OR "internet"[ti] OR "online"[ti] OR "digital"[ti] OR digital*[ti]** OR "Reminder Systems"[mesh] OR "Reminder Systems"[tw] OR "Reminder System"[tw] OR "Reminder Device"[tw] OR "Reminder Devices"[tw] OR "reminder messages"[tw] OR "reminder message"[tw] OR **"web"[ti]** OR "Virtual Reality"[mesh] OR "Virtual Reality"[tw] OR "smart technology"[tw] OR smart technol*[tw] OR "wearable technology"[tw] OR "wearable technologies"[tw] OR "telerehabilitation"[tw] OR "Therapy, computer-assisted"[mesh:noexp] OR "computer-assisted therapy"[tw] OR "computer assisted therapy"[tw] OR "online therapy"[tw] OR "Computer Mediated Communication"[tw] OR "Computer Mediated Communications"[tw] OR **"remote"[ti] OR "on-line"[ti] OR "on line"[ti]** OR "wearable devices"[tw] OR "wearable device"[tw] OR "healthsensor"[tw] OR "healthsensors"[tw] OR "health sensor"[tw] OR "health sensors"[tw] OR "Robotics"[Mesh] OR "robotics"[tw] OR robot*[tw] OR "exergame"[tw] OR "exergames"[tw] OR exergam*[tw] OR "Video Games"[mesh] OR "app-based"[tw] OR "wearable app"[tw] OR "wearable apps"[tw] OR "wearables"[tw] OR "Nintendo"[tw] OR "Wii"[tw] OR "gaming console"[tw] OR "gaming consoles"[tw])

AND ("usability"[tw] OR "Meaningful Use"[Mesh] OR "Meaningful Use"[tw] OR "User experience"[tw] OR "User experiences"[tw] OR "usefulness"[tw]))

Embase
http://ovidsp.ovid.com/ovidweb.cgi?T=JS&PAGE=main&MODE=ovid&D=oemezd

8-1-2021

1.

((exp *"Rehabilitation"/ OR rehab*.ti OR exp *"Rehabilitation Nursing"/ OR *"Rehabilitation Center"/ OR "Activities of Daily Living".ti OR "Animal Assisted Therapy".ti OR "Equine-Assisted Therapy".ti OR "Art Therapy".ti OR "Bibliotherapy".ti OR "Correction of Hearing Impairment".ti OR "Total Communication Methods".ti OR "Lipreading".ti OR "Manual Communication".ti OR "Dance Therapy".ti OR "Early Ambulation".ti OR "Exercise Therapy".ti OR "Endurance Training".ti OR "Continuous Passive Motion Therapy".ti OR "Muscle Stretching".ti OR "Plyometric Exercise".ti OR "Resistance Training".ti OR "Music Therapy".ti OR "Occupational Therapy".ti OR "Recreation Therapy".ti OR "Language Therapy".ti OR "Myofunctional Therapy".ti OR "Speech Therapy".ti OR "Alaryngeal Speech".ti OR "Voice Training".ti OR "Telerehabilitation".ti OR "Activity of Daily Living".ti OR "Plyometric Exercises".ti OR exp *"Exercise"/ OR "exercise".ti OR "exercises".ti OR "physical activity".ti) AND (exp "Aged"/ OR "elderly".ti OR "elder".ti OR "elders".ti OR geriatr*.ti OR exp *"Homes for the Aged"/ OR exp *"Elderly Care"/ OR exp *"Senior Centers"/ OR older person*.ti OR old person*.ti OR older patient*.ti OR old patient*.ti OR "older women".ti OR "old women".ti OR "older men".ti OR "old men".ti OR old adult*.ti OR older adult*.ti OR "Older individual".ti OR "Older individuals".ti OR "old people".ti OR "older people".ti OR "Oldest Old".ti OR "Nonagenarians".ti OR "Nonagenarian".ti OR "Octogenarians".ti OR "Octogenarian".ti OR "Centenarians".ti OR "Centenarian".ti OR "septuagenarian".ti OR "septuagenarians".ti OR exp *"Aging"/ OR "aging".ti OR "ageing".ti OR "older population".ti OR "aging population".ti OR "aging population".ti OR geront*.ti OR "old-aged".ti OR "old-age".ti OR "old aged".ti OR "old age".ti) AND ("accelerometers".ti OR "accelerometer".ti OR "accelerometry".ti OR acceleromet*.ti OR exp *"Telehealth"/ OR "web portals".ti OR "web portal".ti OR "web portal*".ti OR "e-consult*".ti OR econsult*.ti OR telemed*.ti OR "ehealth".ti OR "e-health".ti OR "mhealth".ti OR "m-health".ti OR "mobile health".ti OR "telehealth".ti OR "tele-health".ti OR "tele health".ti OR "**electronic communication*".ti** OR "remote consultation".ti OR "remote health care".ti OR "remote healthcare".ti OR "remote care".ti OR "remote monitoring".ti OR "teleconsultation".ti OR teleconsult*.ti OR **mobile*.ti OR "webbased".ti OR "web-based".ti OR exp *"Electronic Mail"/ OR "Electronic Mail".ti OR "e-mail*".ti OR email*.ti** OR exp *"Mobile Application"/ OR "mobile apps".ti OR "mobile app".ti OR webapp*.ti OR "SMS".ti OR "Cell Phones".ti OR "Smartphone".ti OR "Text Messaging".ti OR exp *"Mobile Phone"/ OR "Cell Phone".ti OR "Smartphones".ti OR iphon*.ti OR "text messag*".ti OR "texting".ti OR **"mobile".ti** OR "cellular phone".ti OR "cellular phones".ti OR "smart phone".ti OR "telemedicine".ti OR "tele-care".ti OR

"telecare".ti OR "tele-monitoring".ti OR "telemonitoring".ti OR **"website".ti OR "websites".ti** OR "personal digital assistant".ti OR "computer-assisted instruction".ti OR "ipad".ti OR ipad*.ti OR "telenursing".ti OR telenurs*.ti OR "virtual community".ti **OR "webpage".ti OR "webpages".ti** OR "web application".ti OR "web applications".ti OR "short message service".ti OR **exp *"Internet"/ OR "internet".ti OR "online".ti OR "digital".ti OR digital*.ti** OR exp *"Reminder System"/ OR "Reminder Systems".ti OR "Reminder System".ti OR "Reminder Device".ti OR "Reminder Devices".ti OR "reminder messages".ti OR "reminder message".ti OR **"web".ti** OR exp *"Virtual Reality"/ OR "Virtual Reality".ti OR "smart technology".ti OR "smart technol*".ti OR "wearable technology".ti OR "wearable technologies".ti OR "telerehabilitation".ti OR "computer-assisted therapy"/ OR "computer-assisted therapy".ti OR "computer assisted therapy".ti OR "online therapy".ti OR "Computer Mediated Communication".ti OR "Computer Mediated Communications".ti OR **"remote".ti OR "on-line".ti OR "on line".ti** OR "wearable devices".ti OR "wearable device".ti OR "healthsensor".ti OR "healthsensors".ti OR "health sensor".ti OR "health sensors".ti OR exp *"Robotics"/ OR "robotics".ti OR robot*.ti OR "exergame".ti OR "exergames".ti OR exergam*.ti OR exp *"Video Game"/ OR "app-based".ti OR "wearable app".ti OR "wearable apps".ti OR "wearables".ti OR "Nintendo".ti OR "Wii".ti OR "gaming console".ti OR "gaming consoles".ti)) NOT (conference review or conference abstract).pt

(2) vier componenten in geval van usability

(((exp *"Rehabilitation"/ OR rehab*.ti OR exp *"Rehabilitation Nursing"/ OR *"Rehabilitation Center"/ OR "Activities of Daily Living".ti OR "Animal Assisted Therapy".ti OR "Equine-Assisted Therapy".ti OR "Art Therapy".ti OR "Bibliotherapy".ti OR "Correction of Hearing Impairment".ti OR "Total Communication Methods".ti OR "Lipreading".ti OR "Manual Communication".ti OR "Dance Therapy".ti OR "Early Ambulation".ti OR "Exercise Therapy".ti OR "Endurance Training".ti OR "Continuous Passive Motion Therapy".ti OR "Muscle Stretching".ti OR "Plyometric Exercise".ti OR "Resistance Training".ti OR "Music Therapy".ti OR "Occupational Therapy".ti OR "Recreation Therapy".ti OR "Language Therapy".ti OR "Myofunctional Therapy".ti OR "Speech Therapy".ti OR "Alaryngeal Speech".ti OR "Voice Training".ti OR "Telerehabilitation".ti OR "Activity of Daily Living".ti OR "Plyometric Exercises".ti OR exp *"Exercise"/ OR "exercise".ti OR "exercises".ti OR "physical activity".ti) AND (exp "Aged"/ OR "elderly".ti OR "elder".ti OR "elders".ti OR geriatr*.ti OR exp *"Homes for the Aged"/ OR exp *"Elderly Care"/ OR exp *"Senior Centers"/ OR older person*.ti OR old person*.ti OR older patient*.ti OR old patient*.ti OR "older women".ti OR "old women".ti OR "older men".ti OR "old men".ti OR old adult*.ti OR older adult*.ti OR "Older individual".ti OR "Older individuals".ti OR "old people".ti OR "older people".ti OR "Oldest Old".ti OR "Nonagenarians".ti OR "Nonagenarian".ti OR "Octogenarians".ti OR "Octogenarian".ti OR "Centenarians".ti OR "Centenarian".ti OR "septuagenarian".ti OR "septuagenarians".ti OR exp *"Aging"/ OR "aging".ti OR "ageing".ti OR "older population".ti OR "aging population".ti OR "aging population".ti OR geront*.ti OR "old-aged".ti OR "old-age".ti OR "old aged".ti OR "old age".ti) AND ("accelerometers".ti,ab OR "accelerometer".ti,ab OR "accelerometry".ti,ab OR acceleromet*.ti,ab OR exp *"Telehealth"/ OR "web portals".ti,ab OR "web portal".ti,ab OR "web portal*".ti,ab OR

"e-consult*".ti,ab OR econsult*.ti,ab OR telemed*.ti,ab OR "ehealth".ti,ab OR "e-health".ti,ab OR "mhealth".ti,ab OR "m-health".ti,ab OR "mobile health".ti,ab OR "telehealth".ti,ab OR "tele-health".ti,ab OR "tele health".ti,ab OR "**electronic communication*".ti** OR "remote consultation".ti,ab OR "remote health care".ti,ab OR "remote healthcare".ti,ab OR "remote care".ti,ab OR "remote monitoring".ti,ab OR "teleconsultation".ti,ab OR teleconsult*.ti,ab OR **mobile*.ti OR "webbased".ti OR "web-based".ti OR exp *"Electronic Mail"/ OR "Electronic Mail".ti OR "e-mail*".ti OR email*.ti** OR exp *"Mobile Application"/ OR "mobile apps".ti,ab OR "mobile app".ti,ab OR webapp*.ti,ab OR "SMS".ti,ab OR "Cell Phones".ti,ab OR "Smartphone".ti,ab OR "Text Messaging".ti,ab OR exp *"Mobile Phone"/ OR "Cell Phone".ti,ab OR "Smartphones".ti,ab OR iphon*.ti,ab OR "text messag*".ti,ab OR "texting".ti,ab OR **"mobile".ti** OR "cellular phone".ti,ab OR "cellular phones".ti,ab OR "smart phone".ti,ab OR "telemedicine".ti,ab OR "tele-care".ti,ab OR "telecare".ti,ab OR "tele-monitoring".ti,ab OR "telemonitoring".ti,ab OR **"website".ti OR "websites".ti** OR "personal digital assistant".ti,ab OR "computer-assisted instruction".ti,ab OR "ipad".ti,ab OR ipad*.ti,ab OR "telenursing".ti,ab OR telenurs*.ti,ab OR "virtual community".ti,ab **OR "webpage".ti OR "webpages".ti** OR "web application".ti,ab OR "web applications".ti,ab OR "short message service".ti,ab OR **exp *"Internet"/ OR "internet".ti OR "online".ti OR "digital".ti OR digital*.ti** OR exp *"Reminder System"/ OR "Reminder Systems".ti,ab OR "Reminder System".ti,ab OR "Reminder Device".ti,ab OR "Reminder Devices".ti,ab OR "reminder messages".ti,ab OR "reminder message".ti,ab OR **"web".ti** OR exp *"Virtual Reality"/ OR "Virtual Reality".ti,ab OR "smart technology".ti,ab OR "smart technol*".ti,ab OR "wearable technology".ti,ab OR "wearable technologies".ti,ab OR "telerehabilitation".ti,ab OR *"computer-assisted therapy"/ OR "computer-assisted therapy".ti,ab OR "computer assisted therapy".ti,ab OR "online therapy".ti,ab OR "Computer Mediated Communication".ti,ab OR "Computer Mediated Communications".ti,ab OR **"remote".ti OR "on-line".ti OR "on line".ti** OR "wearable devices".ti,ab OR "wearable device".ti,ab OR "healthsensor".ti,ab OR "healthsensors".ti,ab OR "health sensor".ti,ab OR "health sensors".ti,ab OR exp *"Robotics"/ OR "robotics".ti,ab OR robot*.ti,ab OR "exergame".ti,ab OR "exergames".ti,ab OR exergam*.ti,ab OR exp *"Video Game"/ OR "app-based".ti,ab OR "wearable app".ti,ab OR "wearable apps".ti,ab OR "wearables".ti,ab OR "Nintendo".ti,ab OR "Wii".ti,ab OR "gaming console".ti,ab OR "gaming consoles".ti,ab)) **OR** ((exp *"Rehabilitation"/ OR rehab*.ti,ab OR exp *"Rehabilitation Nursing"/ OR exp *"Rehabilitation Center"/ OR "Activities of Daily Living".ti,ab OR "Animal Assisted Therapy".ti,ab OR "Equine-Assisted Therapy".ti,ab OR "Art Therapy".ti,ab OR "Bibliotherapy".ti,ab OR "Correction of Hearing Impairment".ti,ab OR "Total Communication Methods".ti,ab OR "Lipreading".ti,ab OR "Manual Communication".ti,ab OR "Dance Therapy".ti,ab OR "Early Ambulation".ti,ab OR "Exercise Therapy".ti,ab OR "Endurance Training".ti,ab OR "Continuous Passive Motion Therapy".ti,ab OR "Muscle Stretching".ti,ab OR "Plyometric Exercise".ti,ab OR "Resistance Training".ti,ab OR "Music Therapy".ti,ab OR "Occupational Therapy".ti,ab OR "Recreation Therapy".ti,ab OR "Language Therapy".ti,ab OR "Myofunctional Therapy".ti,ab OR "Speech Therapy".ti,ab OR "Alaryngeal Speech".ti,ab OR "Voice Training".ti,ab OR "Telerehabilitation".ti,ab OR "Activity of Daily Living".ti,ab OR "Plyometric Exercises".ti,ab OR exp *"Exercise"/ OR "exercise".ti,ab OR

"exercises".ti,ab OR "physical activity".ti,ab) AND (exp "Aged"/ OR "elderly".ti OR "elder".ti OR "elders".ti OR geriatr*.ti OR exp "Homes for the Aged"/ OR exp "Elderly Care"/ OR exp "Senior Centers"/ OR older person*.ti OR old person*.ti OR older patient*.ti OR old patient*.ti OR "older women".ti OR "old women".ti OR "older men".ti OR "old men".ti OR old adult*.ti OR older adult*.ti OR "Older individual".ti OR "Older individuals".ti OR "old people".ti OR "older people".ti OR "Oldest Old".ti OR "Nonagenarians".ti OR "Nonagenarian".ti OR "Octogenarians".ti OR "Octogenarian".ti OR "Centenarians".ti OR "Centenarian".ti OR "septuagenarian".ti OR "septuagenarians".ti OR exp "Aging"/ OR "aging".ti OR "ageing".ti OR "older population".ti OR "aging population".ti OR "aging population".ti OR geront*.ti OR "old-aged".ti OR "old-age".ti OR "old aged".ti OR "old age".ti) AND ("accelerometers".ti,ab OR "accelerometer".ti,ab OR "accelerometry".ti,ab OR acceleromet*.ti,ab OR exp *"Telehealth"/ OR "web portals".ti,ab OR "web portal".ti,ab OR "web portal*".ti,ab OR "e-consult*".ti,ab OR econsult*.ti,ab OR telemed*.ti,ab OR "ehealth".ti,ab OR "e-health".ti,ab OR "mhealth".ti,ab OR "m-health".ti,ab OR "mobile health".ti,ab OR "telehealth".ti,ab OR "tele-health".ti,ab OR "tele health".ti,ab OR "**electronic communication*".ti** OR "remote consultation".ti,ab OR "remote health care".ti,ab OR "remote healthcare".ti,ab OR "remote care".ti,ab OR "remote monitoring".ti,ab OR "teleconsultation".ti,ab OR teleconsult*.ti,ab OR **mobile*.ti OR "webbased".ti OR "web-based".ti OR exp *"Electronic Mail"/ OR "Electronic Mail".ti OR "e-mail*".ti OR email*.ti** OR exp *"Mobile Application"/ OR "mobile apps".ti,ab OR "mobile app".ti,ab OR webapp*.ti,ab OR "SMS".ti,ab OR "Cell Phones".ti,ab OR "Smartphone".ti,ab OR "Text Messaging".ti,ab OR exp *"Mobile Phone"/ OR "Cell Phone".ti,ab OR "Smartphones".ti,ab OR iphon*.ti,ab OR "text messag*".ti,ab OR "texting".ti,ab OR **"mobile".ti** OR "cellular phone".ti,ab OR "cellular phones".ti,ab OR "smart phone".ti,ab OR "telemedicine".ti,ab OR "tele-care".ti,ab OR "telecare".ti,ab OR "tele-monitoring".ti,ab OR "telemonitoring".ti,ab OR **"website".ti OR "websites".ti** OR "personal digital assistant".ti,ab OR "computer-assisted instruction".ti,ab OR "ipad".ti,ab OR ipad*.ti,ab OR "telenursing".ti,ab OR telenurs*.ti,ab OR "virtual community".ti,ab **OR "webpage".ti OR "webpages".ti** OR "web application".ti,ab OR "web applications".ti,ab OR "short message service".ti,ab OR **exp *"Internet"/ OR "internet".ti OR "online".ti OR "digital".ti OR digital*.ti** OR exp *"Reminder System"/ OR "Reminder Systems".ti,ab OR "Reminder System".ti,ab OR "Reminder Device".ti,ab OR "Reminder Devices".ti,ab OR "reminder messages".ti,ab OR "reminder message".ti,ab OR **"web".ti** OR exp *"Virtual Reality"/ OR "Virtual Reality".ti,ab OR "smart technology".ti,ab OR "smart technol*".ti,ab OR "wearable technology".ti,ab OR "wearable technologies".ti,ab OR "telerehabilitation".ti,ab OR "computer-assisted therapy"/ OR "computer-assisted therapy".ti,ab OR "computer assisted therapy".ti,ab OR "online therapy".ti,ab OR "Computer Mediated Communication".ti,ab OR "Computer Mediated Communications".ti,ab OR **"remote".ti OR "on-line".ti OR "on line".ti** OR "wearable devices".ti,ab OR "wearable device".ti,ab OR "healthsensor".ti,ab OR "healthsensors".ti,ab OR "health sensor".ti,ab OR "health sensors".ti,ab OR exp *"Robotics"/ OR "robotics".ti,ab OR robot*.ti,ab OR "exergame".ti,ab OR "exergames".ti,ab OR exergam*.ti,ab OR exp *"Video Game"/ OR "app-based".ti,ab OR "wearable app".ti,ab OR "wearable apps".ti,ab OR "wearables".ti,ab OR "Nintendo".ti,ab OR "Wii".ti,ab OR "gaming console".ti,ab OR "gaming consoles".ti,ab))) NOT (conference

review or conference abstract).pt AND ("usability".mp OR "Meaningful Use".mp OR "User experience".mp OR "User experiences".mp OR "usefulness".mp)

(3) Een nieuwe zoekstring met een omschrijving van de term feasibility. Ook weer met de nadruk op alle vier de componenten

("feasibility".mp OR "feasible".mp OR "Feasibility Study"/ OR "program feasibility"/) AND ((exp *"Rehabilitation"/ OR rehab*.ti OR exp *"Rehabilitation Nursing"/ OR *"Rehabilitation Center"/ OR "Activities of Daily Living".ti OR "Animal Assisted Therapy".ti OR "Equine-Assisted Therapy".ti OR "Art Therapy".ti OR "Bibliotherapy".ti OR "Correction of Hearing Impairment".ti OR "Total Communication Methods".ti OR "Lipreading".ti OR "Manual Communication".ti OR "Dance Therapy".ti OR "Early Ambulation".ti OR "Exercise Therapy".ti OR "Endurance Training".ti OR "Continuous Passive Motion Therapy".ti OR "Muscle Stretching".ti OR "Plyometric Exercise".ti OR "Resistance Training".ti OR "Music Therapy".ti OR "Occupational Therapy".ti OR "Recreation Therapy".ti OR "Language Therapy".ti OR "Myofunctional Therapy".ti OR "Speech Therapy".ti OR "Alaryngeal Speech".ti OR "Voice Training".ti OR "Telerehabilitation".ti OR "Activity of Daily Living".ti OR "Plyometric Exercises".ti OR exp *"Exercise"/ OR "exercise".ti OR "exercises".ti OR "physical activity".ti) AND (exp "Aged"/ OR "elderly".ti OR "elder".ti OR "elders".ti OR geriatr*.ti OR exp *"Homes for the Aged"/ OR exp *"Elderly Care"/ OR exp *"Senior Centers"/ OR older person*.ti OR old person*.ti OR older patient*.ti OR old patient*.ti OR "older women".ti OR "old women".ti OR "older men".ti OR "old men".ti OR old adult*.ti OR older adult*.ti OR "Older individual".ti OR "Older individuals".ti OR "old people".ti OR "older people".ti OR "Oldest Old".ti OR "Nonagenarians".ti OR "Nonagenarian".ti OR "Octogenarians".ti OR "Octogenarian".ti OR "Centenarians".ti OR "Centenarian".ti OR "septuagenarian".ti OR "septuagenarians".ti OR exp *"Aging"/ OR "aging".ti OR "ageing".ti OR "older population".ti OR "aging population".ti OR "aging population".ti OR geront*.ti OR "old-aged".ti OR "old-age".ti OR "old aged".ti OR "old age".ti) AND ("accelerometers".ti OR "accelerometer".ti OR "accelerometry".ti OR acceleromet*.ti OR exp *"Telehealth"/ OR "web portals".ti OR "web portal".ti OR "web portal*".ti OR "e-consult*".ti OR econsult*.ti OR telemed*.ti OR "ehealth".ti OR "e-health".ti OR "mhealth".ti OR "m-health".ti OR "mobile health".ti OR "telehealth".ti OR "tele-health".ti OR "tele health".ti OR "**electronic communication*".ti** OR "remote consultation".ti OR "remote health care".ti OR "remote healthcare".ti OR "remote care".ti OR "remote monitoring".ti OR "teleconsultation".ti OR teleconsult*.ti OR **mobile*.ti OR "webbased".ti OR "web-based".ti OR exp *"Electronic Mail"/ OR "Electronic Mail".ti OR "e-mail*".ti OR email*.ti** OR exp *"Mobile Application"/ OR "mobile apps".ti OR "mobile app".ti OR webapp*.ti OR "SMS".ti OR "Cell Phones".ti OR "Smartphone".ti OR "Text Messaging".ti OR exp *"Mobile Phone"/ OR "Cell Phone".ti OR "Smartphones".ti OR iphon*.ti OR "text messag*".ti OR "texting".ti OR **"mobile".ti** OR "cellular phone".ti OR "cellular phones".ti OR "smart phone".ti OR "telemedicine".ti OR "tele-care".ti OR "telecare".ti OR "tele-monitoring".ti OR "telemonitoring".ti OR **"website".ti OR "websites".ti** OR "personal digital assistant".ti OR "computer-assisted instruction".ti OR "ipad".ti OR ipad*.ti OR "telenursing".ti OR telenurs*.ti OR "virtual community".ti **OR "webpage".ti OR "webpages".ti** OR "web application".ti OR "web applications".ti OR

"short message service".ti OR **exp *"Internet"/ OR "internet".ti OR "online".ti OR "digital".ti OR digital*.ti** OR exp *"Reminder System"/ OR "Reminder Systems".ti OR "Reminder System".ti OR "Reminder Device".ti OR "Reminder Devices".ti OR "reminder messages".ti OR "reminder message".ti OR **"web".ti** OR exp *"Virtual Reality"/ OR "Virtual Reality".ti OR "smart technology".ti OR "smart technol*".ti OR "wearable technology".ti OR "wearable technologies".ti OR "telerehabilitation".ti OR "computer-assisted therapy"/ OR "computer-assisted therapy".ti OR "computer assisted therapy".ti OR "online therapy".ti OR "Computer Mediated Communication".ti OR "Computer Mediated Communications".ti OR **"remote".ti OR "on-line".ti OR "on line".ti** OR "wearable devices".ti OR "wearable device".ti OR "healthsensor".ti OR "healthsensors".ti OR "health sensor".ti OR "health sensors".ti OR exp *"Robotics"/ OR "robotics".ti OR robot*.ti OR "exergame".ti OR "exergames".ti OR exergam*.ti OR exp *"Video Game"/ OR "app-based".ti OR "wearable app".ti OR "wearable apps".ti OR "wearables".ti OR "Nintendo".ti OR "Wii".ti OR "gaming console".ti OR "gaming consoles".ti)) NOT (conference review or conference abstract).pt

10-4-2019

B. Nadruk op Revalidatie en/óf E-health

(((exp *"Rehabilitation"/ OR rehab*.ti OR exp *"Rehabilitation Nursing"/ OR *"Rehabilitation Center"/ OR "Activities of Daily Living".ti OR "Animal Assisted Therapy".ti OR "Equine-Assisted Therapy".ti OR "Art Therapy".ti OR "Bibliotherapy".ti OR "Correction of Hearing Impairment".ti OR "Total Communication Methods".ti OR "Lipreading".ti OR "Manual Communication".ti OR "Dance Therapy".ti OR "Early Ambulation".ti OR "Exercise Therapy".ti OR "Endurance Training".ti OR "Continuous Passive Motion Therapy".ti OR "Muscle Stretching".ti OR "Plyometric Exercise".ti OR "Resistance Training".ti OR "Music Therapy".ti OR "Occupational Therapy".ti OR "Recreation Therapy".ti OR "Language Therapy".ti OR "Myofunctional Therapy".ti OR "Speech Therapy".ti OR "Alaryngeal Speech".ti OR "Voice Training".ti OR "Telerehabilitation".ti OR "Activity of Daily Living".ti OR "Plyometric Exercises".ti OR exp *"Exercise"/ OR "exercise".ti OR "exercises".ti OR "physical activity".ti) AND (exp "Aged"/ OR "elderly".ti OR "elder".ti OR "elders".ti OR geriatr*.ti OR exp *"Homes for the Aged"/ OR exp *"Elderly Care"/ OR exp *"Senior Centers"/ OR older person*.ti OR old person*.ti OR older patient*.ti OR old patient*.ti OR "older women".ti OR "old women".ti OR "older men".ti OR "old men".ti OR old adult*.ti OR older adult*.ti OR "Older individual".ti OR "Older individuals".ti OR "old people".ti OR "older people".ti OR "Oldest Old".ti OR "Nonagenarians".ti OR "Nonagenarian".ti OR "Octogenarians".ti OR "Octogenarian".ti OR "Centenarians".ti OR "Centenarian".ti OR "septuagenarian".ti OR "septuagenarians".ti OR exp *"Aging"/ OR "aging".ti OR "ageing".ti OR "older population".ti OR "aging population".ti OR "aging population".ti OR geront*.ti OR "old-aged".ti OR "old-age".ti OR "old aged".ti OR "old age".ti) AND ("accelerometers".ti,ab OR "accelerometer".ti,ab OR "accelerometry".ti,ab OR acceleromet*.ti,ab OR exp *"Telehealth"/ OR "web portals".ti,ab OR "web portal".ti,ab OR "web portal*".ti,ab OR "e-consult*".ti,ab OR econsult*.ti,ab OR telemed*.ti,ab OR "ehealth".ti,ab OR "e-health".ti,ab OR "mhealth".ti,ab OR "m-health".ti,ab OR "mobile health".ti,ab OR

"telehealth".ti,ab OR "tele-health".ti,ab OR "tele health".ti,ab OR "**electronic communication*".ti** OR "remote consultation".ti,ab OR "remote health care".ti,ab OR "remote healthcare".ti,ab OR "remote care".ti,ab OR "remote monitoring".ti,ab OR "teleconsultation".ti,ab OR teleconsult*.ti,ab OR **mobile*.ti OR "webbased".ti OR "web-based".ti OR exp *"Electronic Mail"/ OR "Electronic Mail".ti OR "e-mail*".ti OR email*.ti** OR exp *"Mobile Application"/ OR "mobile apps".ti,ab OR "mobile app".ti,ab OR webapp*.ti,ab OR "SMS".ti,ab OR "Cell Phones".ti,ab OR "Smartphone".ti,ab OR "Text Messaging".ti,ab OR exp *"Mobile Phone"/ OR "Cell Phone".ti,ab OR "Smartphones".ti,ab OR iphon*.ti,ab OR "text messag*".ti,ab OR "texting".ti,ab OR **"mobile".ti** OR "cellular phone".ti,ab OR "cellular phones".ti,ab OR "smart phone".ti,ab OR "telemedicine".ti,ab OR "tele-care".ti,ab OR "telecare".ti,ab OR "tele-monitoring".ti,ab OR "telemonitoring".ti,ab OR **"website".ti OR "websites".ti** OR "personal digital assistant".ti,ab OR "computer-assisted instruction".ti,ab OR "ipad".ti,ab OR ipad*.ti,ab OR "telenursing".ti,ab OR telenurs*.ti,ab OR "virtual community".ti,ab **OR "webpage".ti OR "webpages".ti** OR "web application".ti,ab OR "web applications".ti,ab OR "short message service".ti,ab OR **exp *"Internet"/ OR "internet".ti OR "online".ti OR "digital".ti OR digital*.ti** OR exp *"Reminder System"/ OR "Reminder Systems".ti,ab OR "Reminder System".ti,ab OR "Reminder Device".ti,ab OR "Reminder Devices".ti,ab OR "reminder messages".ti,ab OR "reminder message".ti,ab OR **"web".ti** OR exp *"Virtual Reality"/ OR "Virtual Reality".ti,ab OR "smart technology".ti,ab OR "smart technol*".ti,ab OR "wearable technology".ti,ab OR "wearable technologies".ti,ab OR "telerehabilitation".ti,ab OR *"computer-assisted therapy"/ OR "computer-assisted therapy".ti,ab OR "computer assisted therapy".ti,ab OR "online therapy".ti,ab OR "Computer Mediated Communication".ti,ab OR "Computer Mediated Communications".ti,ab OR **"remote".ti OR "on-line".ti OR "on line".ti** OR "wearable devices".ti,ab OR "wearable device".ti,ab OR "healthsensor".ti,ab OR "healthsensors".ti,ab OR "health sensor".ti,ab OR "health sensors".ti,ab OR exp *"Robotics"/ OR "robotics".ti,ab OR robot*.ti,ab OR "exergame".ti,ab OR "exergames".ti,ab OR exergam*.ti,ab OR exp *"Video Game"/ OR "app-based".ti,ab OR "wearable app".ti,ab OR "wearable apps".ti,ab OR "wearables".ti,ab OR "Nintendo".ti,ab OR "Wii".ti,ab OR "gaming console".ti,ab OR "gaming consoles".ti,ab)) **OR** ((exp *"Rehabilitation"/ OR rehab*.ti,ab OR exp *"Rehabilitation Nursing"/ OR exp *"Rehabilitation Center"/ OR "Activities of Daily Living".ti,ab OR "Animal Assisted Therapy".ti,ab OR "Equine-Assisted Therapy".ti,ab OR "Art Therapy".ti,ab OR "Bibliotherapy".ti,ab OR "Correction of Hearing Impairment".ti,ab OR "Total Communication Methods".ti,ab OR "Lipreading".ti,ab OR "Manual Communication".ti,ab OR "Dance Therapy".ti,ab OR "Early Ambulation".ti,ab OR "Exercise Therapy".ti,ab OR "Endurance Training".ti,ab OR "Continuous Passive Motion Therapy".ti,ab OR "Muscle Stretching".ti,ab OR "Plyometric Exercise".ti,ab OR "Resistance Training".ti,ab OR "Music Therapy".ti,ab OR "Occupational Therapy".ti,ab OR "Recreation Therapy".ti,ab OR "Language Therapy".ti,ab OR "Myofunctional Therapy".ti,ab OR "Speech Therapy".ti,ab OR "Alaryngeal Speech".ti,ab OR "Voice Training".ti,ab OR "Telerehabilitation".ti,ab OR "Activity of Daily Living".ti,ab OR "Plyometric Exercises".ti,ab OR exp *"Exercise"/ OR "exercise".ti,ab OR "exercises".ti,ab OR "physical activity".ti,ab) AND (exp "Aged"/ OR "elderly".ti OR "elder".ti OR "elders".ti OR geriatr*.ti OR exp "Homes for the Aged"/ OR exp "Elderly

Care"/ OR exp "Senior Centers"/ OR older person*.ti OR old person*.ti OR older patient*.ti OR old patient*.ti OR "older women".ti OR "old women".ti OR "older men".ti OR "old men".ti OR old adult*.ti OR older adult*.ti OR "Older individual".ti OR "Older individuals".ti OR "old people".ti OR "older people".ti OR "Oldest Old".ti OR "Nonagenarians".ti OR "Nonagenarian".ti OR "Octogenarians".ti OR "Octogenarian".ti OR "Centenarians".ti OR "Centenarian".ti OR "septuagenarian".ti OR "septuagenarians".ti OR exp "Aging"/ OR "aging".ti OR "ageing".ti OR "older population".ti OR "aging population".ti OR "aging population".ti OR geront*.ti OR "old-aged".ti OR "old-age".ti OR "old aged".ti OR "old age".ti) AND ("accelerometers".ti,ab OR "accelerometer".ti,ab OR "accelerometry".ti,ab OR acceleromet*.ti,ab OR exp *"Telehealth"/ OR "web portals".ti,ab OR "web portal".ti,ab OR "web portal*".ti,ab OR "e-consult*".ti,ab OR econsult*.ti,ab OR telemed*.ti,ab OR "ehealth".ti,ab OR "e-health".ti,ab OR "mhealth".ti,ab OR "m-health".ti,ab OR "mobile health".ti,ab OR "telehealth".ti,ab OR "tele-health".ti,ab OR "tele health".ti,ab OR "**electronic communication*".ti** OR "remote consultation".ti,ab OR "remote health care".ti,ab OR "remote healthcare".ti,ab OR "remote care".ti,ab OR "remote monitoring".ti,ab OR "teleconsultation".ti,ab OR teleconsult*.ti,ab OR **mobile*.ti OR "webbased".ti OR "web-based".ti OR exp *"Electronic Mail"/ OR "Electronic Mail".ti OR "e-mail*".ti OR email*.ti** OR exp *"Mobile Application"/ OR "mobile apps".ti,ab OR "mobile app".ti,ab OR webapp*.ti,ab OR "SMS".ti,ab OR "Cell Phones".ti,ab OR "Smartphone".ti,ab OR "Text Messaging".ti,ab OR exp *"Mobile Phone"/ OR "Cell Phone".ti,ab OR "Smartphones".ti,ab OR iphon*.ti,ab OR "text messag*".ti,ab OR "texting".ti,ab OR **"mobile".ti** OR "cellular phone".ti,ab OR "cellular phones".ti,ab OR "smart phone".ti,ab OR "telemedicine".ti,ab OR "tele-care".ti,ab OR "telecare".ti,ab OR "tele-monitoring".ti,ab OR "telemonitoring".ti,ab OR **"website".ti OR "websites".ti** OR "personal digital assistant".ti,ab OR "computer-assisted instruction".ti,ab OR "ipad".ti,ab OR ipad*.ti,ab OR "telenursing".ti,ab OR telenurs*.ti,ab OR "virtual community".ti,ab **OR "webpage".ti OR "webpages".ti** OR "web application".ti,ab OR "web applications".ti,ab OR "short message service".ti,ab OR **exp *"Internet"/ OR "internet".ti OR "online".ti OR "digital".ti OR digital*.ti** OR exp *"Reminder System"/ OR "Reminder Systems".ti,ab OR "Reminder System".ti,ab OR "Reminder Device".ti,ab OR "Reminder Devices".ti,ab OR "reminder messages".ti,ab OR "reminder message".ti,ab OR **"web".ti** OR exp *"Virtual Reality"/ OR "Virtual Reality".ti,ab OR "smart technology".ti,ab OR "smart technol*".ti,ab OR "wearable technology".ti,ab OR "wearable technologies".ti,ab OR "telerehabilitation".ti,ab OR "computer-assisted therapy"/ OR "computer-assisted therapy".ti,ab OR "computer assisted therapy".ti,ab OR "online therapy".ti,ab OR "Computer Mediated Communication".ti,ab OR "Computer Mediated Communications".ti,ab OR **"remote".ti OR "on-line".ti OR "on line".ti** OR "wearable devices".ti,ab OR "wearable device".ti,ab OR "healthsensor".ti,ab OR "healthsensors".ti,ab OR "health sensor".ti,ab OR "health sensors".ti,ab OR exp *"Robotics"/ OR "robotics".ti,ab OR robot*.ti,ab OR "exergame".ti,ab OR "exergames".ti,ab OR exergam*.ti,ab OR exp *"Video Game"/ OR "app-based".ti,ab OR "wearable app".ti,ab OR "wearable apps".ti,ab OR "wearables".ti,ab OR "Nintendo".ti,ab OR "Wii".ti,ab OR "gaming console".ti,ab OR "gaming consoles".ti,ab))) NOT (conference review or conference abstract).pt

B.2 - Nadruk op Revalidatie en/óf E-health – usability

(((exp *"Rehabilitation"/ OR rehab*.ti OR exp *"Rehabilitation Nursing"/ OR *"Rehabilitation Center"/ OR "Activities of Daily Living".ti OR "Animal Assisted Therapy".ti OR "Equine-Assisted Therapy".ti OR "Art Therapy".ti OR "Bibliotherapy".ti OR "Correction of Hearing Impairment".ti OR "Total Communication Methods".ti OR "Lipreading".ti OR "Manual Communication".ti OR "Dance Therapy".ti OR "Early Ambulation".ti OR "Exercise Therapy".ti OR "Endurance Training".ti OR "Continuous Passive Motion Therapy".ti OR "Muscle Stretching".ti OR "Plyometric Exercise".ti OR "Resistance Training".ti OR "Music Therapy".ti OR "Occupational Therapy".ti OR "Recreation Therapy".ti OR "Language Therapy".ti OR "Myofunctional Therapy".ti OR "Speech Therapy".ti OR "Alaryngeal Speech".ti OR "Voice Training".ti OR "Telerehabilitation".ti OR "Activity of Daily Living".ti OR "Plyometric Exercises".ti OR exp *"Exercise"/ OR "exercise".ti OR "exercises".ti OR "physical activity".ti) AND (exp "Aged"/ OR "elderly".ti OR "elder".ti OR "elders".ti OR geriatr*.ti OR exp *"Homes for the Aged"/ OR exp *"Elderly Care"/ OR exp *"Senior Centers"/ OR older person*.ti OR old person*.ti OR older patient*.ti OR old patient*.ti OR "older women".ti OR "old women".ti OR "older men".ti OR "old men".ti OR old adult*.ti OR older adult*.ti OR "Older individual".ti OR "Older individuals".ti OR "old people".ti OR "older people".ti OR "Oldest Old".ti OR "Nonagenarians".ti OR "Nonagenarian".ti OR "Octogenarians".ti OR "Octogenarian".ti OR "Centenarians".ti OR "Centenarian".ti OR "septuagenarian".ti OR "septuagenarians".ti OR exp *"Aging"/ OR "aging".ti OR "ageing".ti OR "older population".ti OR "aging population".ti OR "aging population".ti OR geront*.ti OR "old-aged".ti OR "old-age".ti OR "old aged".ti OR "old age".ti) AND ("accelerometers".ti,ab OR "accelerometer".ti,ab OR "accelerometry".ti,ab OR acceleromet*.ti,ab OR exp *"Telehealth"/ OR "web portals".ti,ab OR "web portal".ti,ab OR "web portal*".ti,ab OR "e-consult*".ti,ab OR econsult*.ti,ab OR telemed*.ti,ab OR "ehealth".ti,ab OR "e-health".ti,ab OR "mhealth".ti,ab OR "m-health".ti,ab OR "mobile health".ti,ab OR "telehealth".ti,ab OR "tele-health".ti,ab OR "tele health".ti,ab OR "**electronic communication*".ti** OR "remote consultation".ti,ab OR "remote health care".ti,ab OR "remote healthcare".ti,ab OR "remote care".ti,ab OR "remote monitoring".ti,ab OR "teleconsultation".ti,ab OR teleconsult*.ti,ab OR **mobile*.ti OR "webbased".ti OR "web-based".ti OR exp *"Electronic Mail"/ OR "Electronic Mail".ti OR "e-mail*".ti OR email*.ti** OR exp *"Mobile Application"/ OR "mobile apps".ti,ab OR "mobile app".ti,ab OR webapp*.ti,ab OR "SMS".ti,ab OR "Cell Phones".ti,ab OR "Smartphone".ti,ab OR "Text Messaging".ti,ab OR exp *"Mobile Phone"/ OR "Cell Phone".ti,ab OR "Smartphones".ti,ab OR iphon*.ti,ab OR "text messag*".ti,ab OR "texting".ti,ab OR **"mobile".ti** OR "cellular phone".ti,ab OR "cellular phones".ti,ab OR "smart phone".ti,ab OR "telemedicine".ti,ab OR "tele-care".ti,ab OR "telecare".ti,ab OR "tele-monitoring".ti,ab OR "telemonitoring".ti,ab OR **"website".ti OR "websites".ti** OR "personal digital assistant".ti,ab OR "computer-assisted instruction".ti,ab OR "ipad".ti,ab OR ipad*.ti,ab OR "telenursing".ti,ab OR telenurs*.ti,ab OR "virtual community".ti,ab **OR "webpage".ti OR "webpages".ti** OR "web application".ti,ab OR "web applications".ti,ab OR "short message service".ti,ab OR **exp *"Internet"/ OR "internet".ti OR "online".ti OR "digital".ti OR digital*.ti** OR exp *"Reminder System"/ OR "Reminder Systems".ti,ab OR "Reminder System".ti,ab OR "Reminder

Device".ti,ab OR "Reminder Devices".ti,ab OR "reminder messages".ti,ab OR "reminder message".ti,ab OR **"web".ti** OR exp *"Virtual Reality"/ OR "Virtual Reality".ti,ab OR "smart technology".ti,ab OR "smart technol*".ti,ab OR "wearable technology".ti,ab OR "wearable technologies".ti,ab OR "telerehabilitation".ti,ab OR *"computer-assisted therapy"/ OR "computer-assisted therapy".ti,ab OR "computer assisted therapy".ti,ab OR "online therapy".ti,ab OR "Computer Mediated Communication".ti,ab OR "Computer Mediated Communications".ti,ab OR **"remote".ti OR "on-line".ti OR "on line".ti** OR "wearable devices".ti,ab OR "wearable device".ti,ab OR "healthsensor".ti,ab OR "healthsensors".ti,ab OR "health sensor".ti,ab OR "health sensors".ti,ab OR exp *"Robotics"/ OR "robotics".ti,ab OR robot*.ti,ab OR "exergame".ti,ab OR "exergames".ti,ab OR exergam*.ti,ab OR exp *"Video Game"/ OR "app-based".ti,ab OR "wearable app".ti,ab OR "wearable apps".ti,ab OR "wearables".ti,ab OR "Nintendo".ti,ab OR "Wii".ti,ab OR "gaming console".ti,ab OR "gaming consoles".ti,ab)) **OR** ((exp *"Rehabilitation"/ OR rehab*.ti,ab OR exp *"Rehabilitation Nursing"/ OR exp *"Rehabilitation Center"/ OR "Activities of Daily Living".ti,ab OR "Animal Assisted Therapy".ti,ab OR "Equine-Assisted Therapy".ti,ab OR "Art Therapy".ti,ab OR "Bibliotherapy".ti,ab OR "Correction of Hearing Impairment".ti,ab OR "Total Communication Methods".ti,ab OR "Lipreading".ti,ab OR "Manual Communication".ti,ab OR "Dance Therapy".ti,ab OR "Early Ambulation".ti,ab OR "Exercise Therapy".ti,ab OR "Endurance Training".ti,ab OR "Continuous Passive Motion Therapy".ti,ab OR "Muscle Stretching".ti,ab OR "Plyometric Exercise".ti,ab OR "Resistance Training".ti,ab OR "Music Therapy".ti,ab OR "Occupational Therapy".ti,ab OR "Recreation Therapy".ti,ab OR "Language Therapy".ti,ab OR "Myofunctional Therapy".ti,ab OR "Speech Therapy".ti,ab OR "Alaryngeal Speech".ti,ab OR "Voice Training".ti,ab OR "Telerehabilitation".ti,ab OR "Activity of Daily Living".ti,ab OR "Plyometric Exercises".ti,ab OR exp *"Exercise"/ OR "exercise".ti,ab OR "exercises".ti,ab OR "physical activity".ti,ab) AND (exp "Aged"/ OR "elderly".ti OR "elder".ti OR "elders".ti OR geriatr*.ti OR exp "Homes for the Aged"/ OR exp "Elderly Care"/ OR exp "Senior Centers"/ OR older person*.ti OR old person*.ti OR older patient*.ti OR old patient*.ti OR "older women".ti OR "old women".ti OR "older men".ti OR "old men".ti OR old adult*.ti OR older adult*.ti OR "Older individual".ti OR "Older individuals".ti OR "old people".ti OR "older people".ti OR "Oldest Old".ti OR "Nonagenarians".ti OR "Nonagenarian".ti OR "Octogenarians".ti OR "Octogenarian".ti OR "Centenarians".ti OR "Centenarian".ti OR "septuagenarian".ti OR "septuagenarians".ti OR exp "Aging"/ OR "aging".ti OR "ageing".ti OR "older population".ti OR "aging population".ti OR "aging population".ti OR geront*.ti OR "old-aged".ti OR "old-age".ti OR "old aged".ti OR "old age".ti) AND ("accelerometers".ti,ab OR "accelerometer".ti,ab OR "accelerometry".ti,ab OR acceleromet*.ti,ab OR exp *"Telehealth"/ OR "web portals".ti,ab OR "web portal".ti,ab OR "web portal*".ti,ab OR "e-consult*".ti,ab OR econsult*.ti,ab OR telemed*.ti,ab OR "ehealth".ti,ab OR "e-health".ti,ab OR "mhealth".ti,ab OR "m-health".ti,ab OR "mobile health".ti,ab OR "telehealth".ti,ab OR "tele-health".ti,ab OR "tele health".ti,ab OR "**electronic communication*".ti** OR "remote consultation".ti,ab OR "remote health care".ti,ab OR "remote healthcare".ti,ab OR "remote care".ti,ab OR "remote monitoring".ti,ab OR "teleconsultation".ti,ab OR teleconsult*.ti,ab OR **mobile*.ti OR "webbased".ti OR "web-based".ti OR exp *"Electronic Mail"/ OR "Electronic Mail".ti OR "e-**

mail*".ti OR email*.ti OR exp *"Mobile Application"/ OR "mobile apps".ti,ab OR "mobile app".ti,ab OR webapp*.ti,ab OR "SMS".ti,ab OR "Cell Phones".ti,ab OR "Smartphone".ti,ab OR "Text Messaging".ti,ab OR exp *"Mobile Phone"/ OR "Cell Phone".ti,ab OR "Smartphones".ti,ab OR iphon*.ti,ab OR "text messag*".ti,ab OR "texting".ti,ab OR **"mobile".ti** OR "cellular phone".ti,ab OR "cellular phones".ti,ab OR "smart phone".ti,ab OR "telemedicine".ti,ab OR "tele-care".ti,ab OR "telecare".ti,ab OR "tele-monitoring".ti,ab OR "telemonitoring".ti,ab OR **"website".ti OR "websites".ti** OR "personal digital assistant".ti,ab OR "computer-assisted instruction".ti,ab OR "ipad".ti,ab OR ipad*.ti,ab OR "telenursing".ti,ab OR telenurs*.ti,ab OR "virtual community".ti,ab **OR "webpage".ti OR "webpages".ti** OR "web application".ti,ab OR "web applications".ti,ab OR "short message service".ti,ab OR **exp *"Internet"/ OR "internet".ti OR "online".ti OR "digital".ti OR digital*.ti** OR exp *"Reminder System"/ OR "Reminder Systems".ti,ab OR "Reminder System".ti,ab OR "Reminder Device".ti,ab OR "Reminder Devices".ti,ab OR "reminder messages".ti,ab OR "reminder message".ti,ab OR **"web".ti** OR exp *"Virtual Reality"/ OR "Virtual Reality".ti,ab OR "smart technology".ti,ab OR "smart technol*".ti,ab OR "wearable technology".ti,ab OR "wearable technologies".ti,ab OR "telerehabilitation".ti,ab OR "computer-assisted therapy"/ OR "computer-assisted therapy".ti,ab OR "computer assisted therapy".ti,ab OR "online therapy".ti,ab OR "Computer Mediated Communication".ti,ab OR "Computer Mediated Communications".ti,ab OR **"remote".ti OR "on-line".ti OR "on line".ti** OR "wearable devices".ti,ab OR "wearable device".ti,ab OR "healthsensor".ti,ab OR "healthsensors".ti,ab OR "health sensor".ti,ab OR "health sensors".ti,ab OR exp *"Robotics"/ OR "robotics".ti,ab OR robot*.ti,ab OR "exergame".ti,ab OR "exergames".ti,ab OR exergam*.ti,ab OR exp *"Video Game"/ OR "app-based".ti,ab OR "wearable app".ti,ab OR "wearable apps".ti,ab OR "wearables".ti,ab OR "Nintendo".ti,ab OR "Wii".ti,ab OR "gaming console".ti,ab OR "gaming consoles".ti,ab))**)** NOT (conference review or conference abstract).pt AND ("usability".mp OR "Meaningful Use".mp OR "User experience".mp OR "User experiences".mp OR "usefulness".mp)

Web of Science
http://isiknowledge.com/wos

8-1-2021

1.

(ti=("Rehabilitation" OR rehab* OR "Rehabilitation Nursing" OR "Rehabilitation Center" OR "Activities of Daily Living" OR "Animal Assisted Therapy" OR "Equine-Assisted Therapy" OR "Art Therapy" OR "Bibliotherapy" OR "Correction of Hearing Impairment" OR "Total Communication Methods" OR "Lipreading" OR "Manual Communication" OR "Dance Therapy" OR "Early Ambulation" OR "Exercise Therapy" OR "Endurance Training" OR "Continuous Passive Motion Therapy" OR "Muscle Stretching" OR "Plyometric Exercise" OR "Resistance Training" OR "Music Therapy" OR "Occupational Therapy" OR "Recreation Therapy" OR "Language Therapy" OR "Myofunctional Therapy" OR "Speech Therapy" OR "Alaryngeal Speech" OR "Voice

Training" OR "Telerehabilitation" OR "Activity of Daily Living" OR "Plyometric Exercises" OR "Exercise" OR "exercise" OR "exercises" OR "physical activity") AND ti=("elderly" OR "elder" OR "elders" OR geriatr* OR "Homes for the Aged" OR "Elderly Care" OR "Senior Centers" OR older person* OR old person* OR older patient* OR old patient* OR "older women" OR "old women" OR "older men" OR "old men" OR old adult* OR older adult* OR "Older individual" OR "Older individuals" OR "old people" OR "older people" OR "Oldest Old" OR "Nonagenarians" OR "Nonagenarian" OR "Octogenarians" OR "Octogenarian" OR "Centenarians" OR "Centenarian" OR "septuagenarian" OR "septuagenarians" OR "Aging" OR "aging" OR "ageing" OR "older population" OR "aging population" OR "aging population" OR geront* OR "old-aged" OR "old-age" OR "old aged" OR "old age") AND ti=("accelerometers" OR "accelerometer" OR "accelerometry" OR acceleromet* OR "Telehealth" OR "web portals" OR "web portal" OR "web portal" OR "e-consult" OR econsult* OR telemed* OR "ehealth" OR "e-health" OR "mhealth" OR "m-health" OR "mobile health" OR "telehealth" OR "tele-health" OR "tele health" OR "**electronic communication**" OR "remote consultation" OR "remote health care" OR "remote healthcare" OR "remote care" OR "remote monitoring" OR "teleconsultation" OR teleconsult* OR **mobile* OR "webbased" OR "web-based" OR "Electronic Mail" OR "Electronic Mail" OR "e-mail" OR email*** OR "Mobile Application" OR "mobile apps" OR "mobile app" OR webapp* OR "SMS" OR "Cell Phones" OR "Smartphone" OR "Text Messaging" OR "Mobile Phone" OR "Cell Phone" OR "Smartphones" OR iphon* OR "text messag" OR "texting" OR **"mobile"** OR "cellular phone" OR "cellular phones" OR "smart phone" OR "telemedicine" OR "tele-care" OR "telecare" OR "tele-monitoring" OR "telemonitoring" OR **"website" OR "websites"** OR "personal digital assistant" OR "computer-assisted instruction" OR "ipad" OR ipad* OR "telenursing" OR telenurs* OR "virtual community" **OR "webpage" OR "webpages"** OR "web application" OR "web applications" OR "short message service" OR **"Internet" OR "internet" OR "online" OR "digital" OR digital*** OR "Reminder System" OR "Reminder Systems" OR "Reminder System" OR "Reminder Device" OR "Reminder Devices" OR "reminder messages" OR "reminder message" OR **"web"** OR "Virtual Reality" OR "Virtual Reality" OR "smart technology" OR "smart technol" OR "wearable technology" OR "wearable technologies" OR "telerehabilitation" OR "computer-assisted therapy" OR "computer-assisted therapy" OR "computer assisted therapy" OR "online therapy" OR "Computer Mediated Communication" OR "Computer Mediated Communications" OR **"remote" OR "on-line" OR "on line"** OR "wearable devices" OR "wearable device" OR "healthsensor" OR "healthsensors" OR "health sensor" OR "health sensors" OR "Robotics" OR "robotics" OR robot* OR "exergame" OR "exergames" OR exergam* OR "Video Game" OR "app-based" OR "wearable app" OR "wearable apps" OR "wearables" OR "Nintendo" OR "Wii" OR "gaming console" OR "gaming consoles")) NOT dt=(meeting abstract)

(2) vier componenten in geval van usability

((ti=("Rehabilitation" OR rehab* OR "Rehabilitation Nursing" OR "Rehabilitation Center" OR "Activities of Daily Living" OR "Animal Assisted Therapy" OR "Equine-Assisted Therapy" OR "Art Therapy" OR "Bibliotherapy" OR "Correction of Hearing

Impairment" OR "Total Communication Methods" OR "Lipreading" OR "Manual Communication" OR "Dance Therapy" OR "Early Ambulation" OR "Exercise Therapy" OR "Endurance Training" OR "Continuous Passive Motion Therapy" OR "Muscle Stretching" OR "Plyometric Exercise" OR "Resistance Training" OR "Music Therapy" OR "Occupational Therapy" OR "Recreation Therapy" OR "Language Therapy" OR "Myofunctional Therapy" OR "Speech Therapy" OR "Alaryngeal Speech" OR "Voice Training" OR "Telerehabilitation" OR "Activity of Daily Living" OR "Plyometric Exercises" OR "Exercise" OR "exercise" OR "exercises" OR "physical activity") AND ti=("elderly" OR "elder" OR "elders" OR geriatr* OR "Homes for the Aged" OR "Elderly Care" OR "Senior Centers" OR older person* OR old person* OR older patient* OR old patient* OR "older women" OR "old women" OR "older men" OR "old men" OR old adult* OR older adult* OR "Older individual" OR "Older individuals" OR "old people" OR "older people" OR "Oldest Old" OR "Nonagenarians" OR "Nonagenarian" OR "Octogenarians" OR "Octogenarian" OR "Centenarians" OR "Centenarian" OR "septuagenarian" OR "septuagenarians" OR "Aging" OR "aging" OR "ageing" OR "older population" OR "aging population" OR "aging population" OR geront* OR "old-aged" OR "old-age" OR "old aged" OR "old age") AND ts=("accelerometers" OR "accelerometer" OR "accelerometry" OR acceleromet* OR "Telehealth" OR "web portals" OR "web portal" OR "web portal" OR "e-consult" OR econsult* OR telemed* OR "ehealth" OR "e-health" OR "mhealth" OR "m-health" OR "mobile health" OR "telehealth" OR "tele-health" OR "tele health" OR "**electronic communication"** OR "remote consultation" OR "remote health care" OR "remote healthcare" OR "remote care" OR "remote monitoring" OR "teleconsultation" OR teleconsult* OR **mobile* OR "webbased" OR "web-based" OR "Electronic Mail" OR "Electronic Mail" OR "e-mail" OR email*** OR "Mobile Application" OR "mobile apps" OR "mobile app" OR webapp* OR "SMS" OR "Cell Phones" OR "Smartphone" OR "Text Messaging" OR "Mobile Phone" OR "Cell Phone" OR "Smartphones" OR iphon* OR "text messag" OR "texting" OR **"mobile"** OR "cellular phone" OR "cellular phones" OR "smart phone" OR "telemedicine" OR "tele-care" OR "telecare" OR "tele-monitoring" OR "telemonitoring" OR **"website" OR "websites"** OR "personal digital assistant" OR "computer-assisted instruction" OR "ipad" OR ipad* OR "telenursing" OR telenurs* OR "virtual community" **OR "webpage" OR "webpages"** OR "web application" OR "web applications" OR "short message service" OR **"Internet" OR "internet" OR "online" OR "digital" OR digital*** OR "Reminder System" OR "Reminder Systems" OR "Reminder System" OR "Reminder Device" OR "Reminder Devices" OR "reminder messages" OR "reminder message" OR **"web"** OR "Virtual Reality" OR "Virtual Reality" OR "smart technology" OR "smart technol" OR "wearable technology" OR "wearable technologies" OR "telerehabilitation" OR "computer-assisted therapy" OR "computer-assisted therapy" OR "computer assisted therapy" OR "online therapy" OR "Computer Mediated Communication" OR "Computer Mediated Communications" OR **"remote" OR "on-line" OR "on line"** OR "wearable devices" OR "wearable device" OR "healthsensor" OR "healthsensors" OR "health sensor" OR "health sensors" OR "Robotics" OR "robotics" OR robot* OR "exergame" OR "exergames" OR exergam* OR "Video Game" OR "app-based" OR "wearable app" OR "wearable apps" OR "wearables" OR "Nintendo" OR "Wii" OR "gaming console" OR "gaming consoles"))
OR (ts=("Rehabilitation" OR rehab* OR "Rehabilitation Nursing" OR "Rehabilitation

Center" OR "Activities of Daily Living" OR "Animal Assisted Therapy" OR "Equine-Assisted Therapy" OR "Art Therapy" OR "Bibliotherapy" OR "Correction of Hearing Impairment" OR "Total Communication Methods" OR "Lipreading" OR "Manual Communication" OR "Dance Therapy" OR "Early Ambulation" OR "Exercise Therapy" OR "Endurance Training" OR "Continuous Passive Motion Therapy" OR "Muscle Stretching" OR "Plyometric Exercise" OR "Resistance Training" OR "Music Therapy" OR "Occupational Therapy" OR "Recreation Therapy" OR "Language Therapy" OR "Myofunctional Therapy" OR "Speech Therapy" OR "Alaryngeal Speech" OR "Voice Training" OR "Telerehabilitation" OR "Activity of Daily Living" OR "Plyometric Exercises" OR "Exercise" OR "exercise" OR "exercises" OR "physical activity") AND ti=("elderly" OR "elder" OR "elders" OR geriatr* OR "Homes for the Aged" OR "Elderly Care" OR "Senior Centers" OR older person* OR old person* OR older patient* OR old patient* OR "older women" OR "old women" OR "older men" OR "old men" OR old adult* OR older adult* OR "Older individual" OR "Older individuals" OR "old people" OR "older people" OR "Oldest Old" OR "Nonagenarians" OR "Nonagenarian" OR "Octogenarians" OR "Octogenarian" OR "Centenarians" OR "Centenarian" OR "septuagenarian" OR "septuagenarians" OR "Aging" OR "aging" OR "ageing" OR "older population" OR "aging population" OR "aging population" OR geront* OR "old-aged" OR "old-age" OR "old aged" OR "old age") AND ti=("accelerometers" OR "accelerometer" OR "accelerometry" OR acceleromet* OR "Telehealth" OR "web portals" OR "web portal" OR "web portal" OR "e-consult" OR econsult* OR telemed* OR "ehealth" OR "e-health" OR "mhealth" OR "m-health" OR "mobile health" OR "telehealth" OR "tele-health" OR "tele health" OR "**electronic communication**" OR "remote consultation" OR "remote health care" OR "remote healthcare" OR "remote care" OR "remote monitoring" OR "teleconsultation" OR teleconsult* OR **mobile* OR "webbased" OR "web-based" OR "Electronic Mail" OR "Electronic Mail" OR "e-mail" OR email*** OR "Mobile Application" OR "mobile apps" OR "mobile app" OR webapp* OR "SMS" OR "Cell Phones" OR "Smartphone" OR "Text Messaging" OR "Mobile Phone" OR "Cell Phone" OR "Smartphones" OR iphon* OR "text messag" OR "texting" OR **"mobile"** OR "cellular phone" OR "cellular phones" OR "smart phone" OR "telemedicine" OR "tele-care" OR "telecare" OR "tele-monitoring" OR "telemonitoring" OR **"website" OR "websites"** OR "personal digital assistant" OR "computer-assisted instruction" OR "ipad" OR ipad* OR "telenursing" OR telenurs* OR "virtual community" **OR "webpage" OR "webpages"** OR "web application" OR "web applications" OR "short message service" OR **"Internet" OR "internet" OR "online" OR "digital" OR digital*** OR "Reminder System" OR "Reminder Systems" OR "Reminder System" OR "Reminder Device" OR "Reminder Devices" OR "reminder messages" OR "reminder message" OR **"web"** OR "Virtual Reality" OR "Virtual Reality" OR "smart technology" OR "smart technol" OR "wearable technology" OR "wearable technologies" OR "telerehabilitation" OR "computer-assisted therapy" OR "computer-assisted therapy" OR "computer assisted therapy" OR "online therapy" OR "Computer Mediated Communication" OR "Computer Mediated Communications" OR **"remote" OR "on-line" OR "on line"** OR "wearable devices" OR "wearable device" OR "healthsensor" OR "healthsensors" OR "health sensor" OR "health sensors" OR "Robotics" OR "robotics" OR robot* OR "exergame" OR "exergames" OR exergam* OR "Video Game" OR "app-based" OR "wearable app" OR "wearable apps" OR

"wearables" OR "Nintendo" OR "Wii" OR "gaming console" OR "gaming consoles")))
AND ts=("usability" OR "Meaningful Use" OR "User experience" OR "User experiences" OR "usefulness")

(3) Een nieuwe zoekstring met een omschrijving van de term feasibility. Ook weer met de nadruk op alle vier de componenten

TS=("feasibility" OR "feasible") AND (ti=("Rehabilitation" OR rehab* OR "Rehabilitation Nursing" OR "Rehabilitation Center" OR "Activities of Daily Living" OR "Animal Assisted Therapy" OR "Equine-Assisted Therapy" OR "Art Therapy" OR "Bibliotherapy" OR "Correction of Hearing Impairment" OR "Total Communication Methods" OR "Lipreading" OR "Manual Communication" OR "Dance Therapy" OR "Early Ambulation" OR "Exercise Therapy" OR "Endurance Training" OR "Continuous Passive Motion Therapy" OR "Muscle Stretching" OR "Plyometric Exercise" OR "Resistance Training" OR "Music Therapy" OR "Occupational Therapy" OR "Recreation Therapy" OR "Language Therapy" OR "Myofunctional Therapy" OR "Speech Therapy" OR "Alaryngeal Speech" OR "Voice Training" OR "Telerehabilitation" OR "Activity of Daily Living" OR "Plyometric Exercises" OR "Exercise" OR "exercise" OR "exercises" OR "physical activity") AND ti=("elderly" OR "elder" OR "elders" OR geriatr* OR "Homes for the Aged" OR "Elderly Care" OR "Senior Centers" OR older person* OR old person* OR older patient* OR old patient* OR "older women" OR "old women" OR "older men" OR "old men" OR old adult* OR older adult* OR "Older individual" OR "Older individuals" OR "old people" OR "older people" OR "Oldest Old" OR "Nonagenarians" OR "Nonagenarian" OR "Octogenarians" OR "Octogenarian" OR "Centenarians" OR "Centenarian" OR "septuagenarian" OR "septuagenarians" OR "Aging" OR "aging" OR "ageing" OR "older population" OR "aging population" OR "aging population" OR geront* OR "old-aged" OR "old-age" OR "old aged" OR "old age") AND ti=("accelerometers" OR "accelerometer" OR "accelerometry" OR acceleromet* OR "Telehealth" OR "web portals" OR "web portal" OR "web portal" OR "e-consult" OR econsult* OR telemed* OR "ehealth" OR "e-health" OR "mhealth" OR "m-health" OR "mobile health" OR "telehealth" OR "tele-health" OR "tele health" OR **"electronic communication"** OR "remote consultation" OR "remote health care" OR "remote healthcare" OR "remote care" OR "remote monitoring" OR "teleconsultation" OR teleconsult* OR **mobile* OR "webbased" OR "web-based" OR "Electronic Mail" OR "Electronic Mail" OR "e-mail" OR email*** OR "Mobile Application" OR "mobile apps" OR "mobile app" OR webapp* OR "SMS" OR "Cell Phones" OR "Smartphone" OR "Text Messaging" OR "Mobile Phone" OR "Cell Phone" OR "Smartphones" OR iphon* OR "text messag" OR "texting" OR **"mobile"** OR "cellular phone" OR "cellular phones" OR "smart phone" OR "telemedicine" OR "tele-care" OR "telecare" OR "tele-monitoring" OR "telemonitoring" OR **"website" OR "websites"** OR "personal digital assistant" OR "computer-assisted instruction" OR "ipad" OR ipad* OR "telenursing" OR telenurs* OR "virtual community" **OR "webpage" OR "webpages"** OR "web application" OR "web applications" OR "short message service" OR **"Internet" OR "internet" OR "online" OR "digital" OR digital*** OR "Reminder System" OR "Reminder Systems" OR "Reminder System" OR "Reminder Device" OR "Reminder Devices" OR "reminder messages" OR "reminder message" OR **"web"** OR

"Virtual Reality" OR "Virtual Reality" OR "smart technology" OR "smart technol" OR "wearable technology" OR "wearable technologies" OR "telerehabilitation" OR "computer-assisted therapy" OR "computer-assisted therapy" OR "computer assisted therapy" OR "online therapy" OR "Computer Mediated Communication" OR "Computer Mediated Communications" OR **"remote" OR "on-line" OR "on line"** OR "wearable devices" OR "wearable device" OR "healthsensor" OR "healthsensors" OR "health sensor" OR "health sensors" OR "Robotics" OR "robotics" OR robot* OR "exergame" OR "exergames" OR exergam* OR "Video Game" OR "app-based" OR "wearable app" OR "wearable apps" OR "wearables" OR "Nintendo" OR "Wii" OR "gaming console" OR "gaming consoles")) NOT dt=(meeting abstract)

10-4-2019

B. Nadruk op Revalidatie en/óf E-health

((ti=("Rehabilitation" OR rehab* OR "Rehabilitation Nursing" OR "Rehabilitation Center" OR "Activities of Daily Living" OR "Animal Assisted Therapy" OR "Equine-Assisted Therapy" OR "Art Therapy" OR "Bibliotherapy" OR "Correction of Hearing Impairment" OR "Total Communication Methods" OR "Lipreading" OR "Manual Communication" OR "Dance Therapy" OR "Early Ambulation" OR "Exercise Therapy" OR "Endurance Training" OR "Continuous Passive Motion Therapy" OR "Muscle Stretching" OR "Plyometric Exercise" OR "Resistance Training" OR "Music Therapy" OR "Occupational Therapy" OR "Recreation Therapy" OR "Language Therapy" OR "Myofunctional Therapy" OR "Speech Therapy" OR "Alaryngeal Speech" OR "Voice Training" OR "Telerehabilitation" OR "Activity of Daily Living" OR "Plyometric Exercises" OR "Exercise" OR "exercise" OR "exercises" OR "physical activity") AND ti=("elderly" OR "elder" OR "elders" OR geriatr* OR "Homes for the Aged" OR "Elderly Care" OR "Senior Centers" OR older person* OR old person* OR older patient* OR old patient* OR "older women" OR "old women" OR "older men" OR "old men" OR old adult* OR older adult* OR "Older individual" OR "Older individuals" OR "old people" OR "older people" OR "Oldest Old" OR "Nonagenarians" OR "Nonagenarian" OR "Octogenarians" OR "Octogenarian" OR "Centenarians" OR "Centenarian" OR "septuagenarian" OR "septuagenarians" OR "Aging" OR "aging" OR "ageing" OR "older population" OR "aging population" OR "aging population" OR geront* OR "old-aged" OR "old-age" OR "old aged" OR "old age") AND ts=("accelerometers" OR "accelerometer" OR "accelerometry" OR acceleromet* OR "Telehealth" OR "web portals" OR "web portal" OR "web portal" OR "e-consult" OR econsult* OR telemed* OR "ehealth" OR "e-health" OR "mhealth" OR "m-health" OR "mobile health" OR "telehealth" OR "tele-health" OR "tele health" OR "**electronic communication"** OR "remote consultation" OR "remote health care" OR "remote healthcare" OR "remote care" OR "remote monitoring" OR "teleconsultation" OR teleconsult* OR **mobile* OR "webbased" OR "web-based" OR "Electronic Mail" OR "Electronic Mail" OR "e-mail" OR email*** OR "Mobile Application" OR "mobile apps" OR "mobile app" OR webapp* OR "SMS" OR "Cell Phones" OR "Smartphone" OR "Text Messaging" OR "Mobile Phone" OR "Cell Phone" OR "Smartphones" OR iphon* OR "text messag" OR

"texting" OR **"mobile"** OR "cellular phone" OR "cellular phones" OR "smart phone" OR "telemedicine" OR "tele-care" OR "telecare" OR "tele-monitoring" OR "telemonitoring" OR **"website" OR "websites"** OR "personal digital assistant" OR "computer-assisted instruction" OR "ipad" OR ipad* OR "telenursing" OR telenurs* OR "virtual community" **OR "webpage" OR "webpages"** OR "web application" OR "web applications" OR "short message service" OR **"Internet" OR "internet" OR "online" OR "digital" OR digital*** OR "Reminder System" OR "Reminder Systems" OR "Reminder System" OR "Reminder Device" OR "Reminder Devices" OR "reminder messages" OR "reminder message" OR **"web"** OR "Virtual Reality" OR "Virtual Reality" OR "smart technology" OR "smart technol" OR "wearable technology" OR "wearable technologies" OR "telerehabilitation" OR "computer-assisted therapy" OR "computer-assisted therapy" OR "computer assisted therapy" OR "online therapy" OR "Computer Mediated Communication" OR "Computer Mediated Communications" OR **"remote" OR "on-line" OR "on line"** OR "wearable devices" OR "wearable device" OR "healthsensor" OR "healthsensors" OR "health sensor" OR "health sensors" OR "Robotics" OR "robotics" OR robot* OR "exergame" OR "exergames" OR exergam* OR "Video Game" OR "app-based" OR "wearable app" OR "wearable apps" OR "wearables" OR "Nintendo" OR "Wii" OR "gaming console" OR "gaming consoles"))
OR (ts=("Rehabilitation" OR rehab* OR "Rehabilitation Nursing" OR "Rehabilitation Center" OR "Activities of Daily Living" OR "Animal Assisted Therapy" OR "Equine-Assisted Therapy" OR "Art Therapy" OR "Bibliotherapy" OR "Correction of Hearing Impairment" OR "Total Communication Methods" OR "Lipreading" OR "Manual Communication" OR "Dance Therapy" OR "Early Ambulation" OR "Exercise Therapy" OR "Endurance Training" OR "Continuous Passive Motion Therapy" OR "Muscle Stretching" OR "Plyometric Exercise" OR "Resistance Training" OR "Music Therapy" OR "Occupational Therapy" OR "Recreation Therapy" OR "Language Therapy" OR "Myofunctional Therapy" OR "Speech Therapy" OR "Alaryngeal Speech" OR "Voice Training" OR "Telerehabilitation" OR "Activity of Daily Living" OR "Plyometric Exercises" OR "Exercise" OR "exercise" OR "exercises" OR "physical activity") AND ti=("elderly" OR "elder" OR "elders" OR geriatr* OR "Homes for the Aged" OR "Elderly Care" OR "Senior Centers" OR older person* OR old person* OR older patient* OR old patient* OR "older women" OR "old women" OR "older men" OR "old men" OR old adult* OR older adult* OR "Older individual" OR "Older individuals" OR "old people" OR "older people" OR "Oldest Old" OR "Nonagenarians" OR "Nonagenarian" OR "Octogenarians" OR "Octogenarian" OR "Centenarians" OR "Centenarian" OR "septuagenarian" OR "septuagenarians" OR "Aging" OR "aging" OR "ageing" OR "older population" OR "aging population" OR "aging population" OR geront* OR "old-aged" OR "old-age" OR "old aged" OR "old age") AND ti=("accelerometers" OR "accelerometer" OR "accelerometry" OR acceleromet* OR "Telehealth" OR "web portals" OR "web portal" OR "web portal" OR "e-consult" OR econsult* OR telemed* OR "ehealth" OR "e-health" OR "mhealth" OR "m-health" OR "mobile health" OR "telehealth" OR "tele-health" OR "tele health" OR "**electronic communication"** OR "remote consultation" OR "remote health care" OR "remote healthcare" OR "remote care" OR "remote monitoring" OR "teleconsultation" OR teleconsult* OR **mobile* OR "webbased" OR "web-based" OR "Electronic Mail" OR "Electronic Mail" OR "e-mail" OR email*** OR "Mobile Application" OR "mobile apps" OR "mobile app" OR

webapp* OR "SMS" OR "Cell Phones" OR "Smartphone" OR "Text Messaging" OR "Mobile Phone" OR "Cell Phone" OR "Smartphones" OR iphon* OR "text messag" OR "texting" OR **"mobile"** OR "cellular phone" OR "cellular phones" OR "smart phone" OR "telemedicine" OR "tele-care" OR "telecare" OR "tele-monitoring" OR "telemonitoring" OR **"website" OR "websites"** OR "personal digital assistant" OR "computer-assisted instruction" OR "ipad" OR ipad* OR "telenursing" OR telenurs* OR "virtual community" **OR "webpage" OR "webpages"** OR "web application" OR "web applications" OR "short message service" OR **"Internet" OR "internet" OR "online" OR "digital" OR digital*** OR "Reminder System" OR "Reminder Systems" OR "Reminder System" OR "Reminder Device" OR "Reminder Devices" OR "reminder messages" OR "reminder message" OR **"web"** OR "Virtual Reality" OR "Virtual Reality" OR "smart technology" OR "smart technol" OR "wearable technology" OR "wearable technologies" OR "telerehabilitation" OR "computer-assisted therapy" OR "computer-assisted therapy" OR "computer assisted therapy" OR "online therapy" OR "Computer Mediated Communication" OR "Computer Mediated Communications" OR **"remote" OR "on-line" OR "on line"** OR "wearable devices" OR "wearable device" OR "healthsensor" OR "healthsensors" OR "health sensor" OR "health sensors" OR "Robotics" OR "robotics" OR robot* OR "exergame" OR "exergames" OR exergam* OR "Video Game" OR "app-based" OR "wearable app" OR "wearable apps" OR "wearables" OR "Nintendo" OR "Wii" OR "gaming console" OR "gaming consoles")))

NOT (conference review or conference abstract).pt

B.2 - Nadruk op Revalidatie en/óf E-health – usability

((ti=("Rehabilitation" OR rehab* OR "Rehabilitation Nursing" OR "Rehabilitation Center" OR "Activities of Daily Living" OR "Animal Assisted Therapy" OR "Equine-Assisted Therapy" OR "Art Therapy" OR "Bibliotherapy" OR "Correction of Hearing Impairment" OR "Total Communication Methods" OR "Lipreading" OR "Manual Communication" OR "Dance Therapy" OR "Early Ambulation" OR "Exercise Therapy" OR "Endurance Training" OR "Continuous Passive Motion Therapy" OR "Muscle Stretching" OR "Plyometric Exercise" OR "Resistance Training" OR "Music Therapy" OR "Occupational Therapy" OR "Recreation Therapy" OR "Language Therapy" OR "Myofunctional Therapy" OR "Speech Therapy" OR "Alaryngeal Speech" OR "Voice Training" OR "Telerehabilitation" OR "Activity of Daily Living" OR "Plyometric Exercises" OR "Exercise" OR "exercise" OR "exercises" OR "physical activity") AND ti=("elderly" OR "elder" OR "elders" OR geriatr* OR "Homes for the Aged" OR "Elderly Care" OR "Senior Centers" OR older person* OR old person* OR older patient* OR old patient* OR "older women" OR "old women" OR "older men" OR "old men" OR old adult* OR older adult* OR "Older individual" OR "Older individuals" OR "old people" OR "older people" OR "Oldest Old" OR "Nonagenarians" OR "Nonagenarian" OR "Octogenarians" OR "Octogenarian" OR "Centenarians" OR "Centenarian" OR "septuagenarian" OR "septuagenarians" OR "Aging" OR "aging" OR "ageing" OR "older population" OR "aging population" OR "aging population" OR geront* OR "old-aged" OR "old-age" OR "old aged" OR "old age") AND ts=("accelerometers" OR "accelerometer" OR "accelerometry" OR acceleromet* OR "Telehealth" OR "web

portals" OR "web portal" OR "web portal" OR "e-consult" OR econsult* OR telemed* OR "ehealth" OR "e-health" OR "mhealth" OR "m-health" OR "mobile health" OR "telehealth" OR "tele-health" OR "tele health" OR "**electronic communication"** OR "remote consultation" OR "remote health care" OR "remote healthcare" OR "remote care" OR "remote monitoring" OR "teleconsultation" OR teleconsult* OR **mobile* OR "webbased" OR "web-based" OR "Electronic Mail" OR "Electronic Mail" OR "e-mail" OR email*** OR "Mobile Application" OR "mobile apps" OR "mobile app" OR webapp* OR "SMS" OR "Cell Phones" OR "Smartphone" OR "Text Messaging" OR "Mobile Phone" OR "Cell Phone" OR "Smartphones" OR iphon* OR "text messag" OR "texting" OR **"mobile"** OR "cellular phone" OR "cellular phones" OR "smart phone" OR "telemedicine" OR "tele-care" OR "telecare" OR "tele-monitoring" OR "telemonitoring" OR **"website" OR "websites"** OR "personal digital assistant" OR "computer-assisted instruction" OR "ipad" OR ipad* OR "telenursing" OR telenurs* OR "virtual community" **OR "webpage" OR "webpages"** OR "web application" OR "web applications" OR "short message service" OR **"Internet" OR "internet" OR "online" OR "digital" OR digital*** OR "Reminder System" OR "Reminder Systems" OR "Reminder System" OR "Reminder Device" OR "Reminder Devices" OR "reminder messages" OR "reminder message" OR **"web"** OR "Virtual Reality" OR "Virtual Reality" OR "smart technology" OR "smart technol" OR "wearable technology" OR "wearable technologies" OR "telerehabilitation" OR "computer-assisted therapy" OR "computer-assisted therapy" OR "computer assisted therapy" OR "online therapy" OR "Computer Mediated Communication" OR "Computer Mediated Communications" OR **"remote" OR "on-line" OR "on line"** OR "wearable devices" OR "wearable device" OR "healthsensor" OR "healthsensors" OR "health sensor" OR "health sensors" OR "Robotics" OR "robotics" OR robot* OR "exergame" OR "exergames" OR exergam* OR "Video Game" OR "app-based" OR "wearable app" OR "wearable apps" OR "wearables" OR "Nintendo" OR "Wii" OR "gaming console" OR "gaming consoles"))

OR (ts=("Rehabilitation" OR rehab* OR "Rehabilitation Nursing" OR "Rehabilitation Center" OR "Activities of Daily Living" OR "Animal Assisted Therapy" OR "Equine-Assisted Therapy" OR "Art Therapy" OR "Bibliotherapy" OR "Correction of Hearing Impairment" OR "Total Communication Methods" OR "Lipreading" OR "Manual Communication" OR "Dance Therapy" OR "Early Ambulation" OR "Exercise Therapy" OR "Endurance Training" OR "Continuous Passive Motion Therapy" OR "Muscle Stretching" OR "Plyometric Exercise" OR "Resistance Training" OR "Music Therapy" OR "Occupational Therapy" OR "Recreation Therapy" OR "Language Therapy" OR "Myofunctional Therapy" OR "Speech Therapy" OR "Alaryngeal Speech" OR "Voice Training" OR "Telerehabilitation" OR "Activity of Daily Living" OR "Plyometric Exercises" OR "Exercise" OR "exercise" OR "exercises" OR "physical activity") AND ti=("elderly" OR "elder" OR "elders" OR geriatr* OR "Homes for the Aged" OR "Elderly Care" OR "Senior Centers" OR older person* OR old person* OR older patient* OR old patient* OR "older women" OR "old women" OR "older men" OR "old men" OR old adult* OR older adult* OR "Older individual" OR "Older individuals" OR "old people" OR "older people" OR "Oldest Old" OR "Nonagenarians" OR "Nonagenarian" OR "Octogenarians" OR "Octogenarian" OR "Centenarians" OR "Centenarian" OR "septuagenarian" OR "septuagenarians" OR "Aging" OR "aging" OR "ageing" OR "older population" OR "aging population" OR "aging population" OR geront* OR "old-aged"

OR "old-age" OR "old aged" OR "old age") AND ti=("accelerometers" OR "accelerometer" OR "accelerometry" OR acceleromet* OR "Telehealth" OR "web portals" OR "web portal" OR "web portal" OR "e-consult" OR econsult* OR telemed* OR "ehealth" OR "e-health" OR "mhealth" OR "m-health" OR "mobile health" OR "telehealth" OR "tele-health" OR "tele health" OR "**electronic communication"** OR "remote consultation" OR "remote health care" OR "remote healthcare" OR "remote care" OR "remote monitoring" OR "teleconsultation" OR teleconsult* OR **mobile* OR "webbased" OR "web-based" OR "Electronic Mail" OR "Electronic Mail" OR "e-mail" OR email*** OR "Mobile Application" OR "mobile apps" OR "mobile app" OR webapp* OR "SMS" OR "Cell Phones" OR "Smartphone" OR "Text Messaging" OR "Mobile Phone" OR "Cell Phone" OR "Smartphones" OR iphon* OR "text messag" OR "texting" OR **"mobile"** OR "cellular phone" OR "cellular phones" OR "smart phone" OR "telemedicine" OR "tele-care" OR "telecare" OR "tele-monitoring" OR "telemonitoring" OR **"website" OR "websites"** OR "personal digital assistant" OR "computer-assisted instruction" OR "ipad" OR ipad* OR "telenursing" OR telenurs* OR "virtual community" **OR "webpage" OR "webpages"** OR "web application" OR "web applications" OR "short message service" OR **"Internet" OR "internet" OR "online" OR "digital" OR digital*** OR "Reminder System" OR "Reminder Systems" OR "Reminder System" OR "Reminder Device" OR "Reminder Devices" OR "reminder messages" OR "reminder message" OR **"web"** OR "Virtual Reality" OR "Virtual Reality" OR "smart technology" OR "smart technol" OR "wearable technology" OR "wearable technologies" OR "telerehabilitation" OR "computer-assisted therapy" OR "computer-assisted therapy" OR "computer assisted therapy" OR "online therapy" OR "Computer Mediated Communication" OR "Computer Mediated Communications" OR **"remote" OR "on-line" OR "on line"** OR "wearable devices" OR "wearable device" OR "healthsensor" OR "healthsensors" OR "health sensor" OR "health sensors" OR "Robotics" OR "robotics" OR robot* OR "exergame" OR "exergames" OR exergam* OR "Video Game" OR "app-based" OR "wearable app" OR "wearable apps" OR "wearables" OR "Nintendo" OR "Wii" OR "gaming console" OR "gaming consoles"))**)** AND ts=("usability" OR "Meaningful Use" OR "User experience" OR "User experiences" OR "usefulness")

NOT (conference review or conference abstract).pt

Cochrane
http://www.cochranelibrary.com/

8-1-2021

1.

(("Rehabilitation" OR rehab* OR "Rehabilitation Nursing" OR "Rehabilitation Center" OR "Activities of Daily Living" OR "Animal Assisted Therapy" OR "Equine-Assisted Therapy" OR "Art Therapy" OR "Bibliotherapy" OR "Correction of Hearing Impairment" OR "Total Communication Methods" OR "Lipreading" OR "Manual Communication" OR "Dance Therapy" OR "Early Ambulation" OR "Exercise Therapy"

OR "Endurance Training" OR "Continuous Passive Motion Therapy" OR "Muscle Stretching" OR "Plyometric Exercise" OR "Resistance Training" OR "Music Therapy" OR "Occupational Therapy" OR "Recreation Therapy" OR "Language Therapy" OR "Myofunctional Therapy" OR "Speech Therapy" OR "Alaryngeal Speech" OR "Voice Training" OR "Telerehabilitation" OR "Activity of Daily Living" OR "Plyometric Exercises" OR "Exercise" OR "exercise" OR "exercises" OR "physical activity"):ti AND ("elderly" OR "elder" OR "elders" OR geriatr* OR "Homes for the Aged" OR "Elderly Care" OR "Senior Centers" OR older person* OR old person* OR older patient* OR old patient* OR "older women" OR "old women" OR "older men" OR "old men" OR old adult* OR older adult* OR "Older individual" OR "Older individuals" OR "old people" OR "older people" OR "Oldest Old" OR "Nonagenarians" OR "Nonagenarian" OR "Octogenarians" OR "Octogenarian" OR "Centenarians" OR "Centenarian" OR "septuagenarian" OR "septuagenarians" OR "Aging" OR "aging" OR "ageing" OR "older population" OR "aging population" OR "aging population" OR geront* OR "old-aged" OR "old-age" OR "old aged" OR "old age"):ti AND ("accelerometers" OR "accelerometer" OR "accelerometry" OR acceleromet* OR "Telehealth" OR "web portals" OR "web portal" OR "web portal" OR "e-consult" OR econsult* OR telemed* OR "ehealth" OR "e-health" OR "mhealth" OR "m-health" OR "mobile health" OR "telehealth" OR "tele-health" OR "tele health" OR "**electronic communication"** OR "remote consultation" OR "remote health care" OR "remote healthcare" OR "remote care" OR "remote monitoring" OR "teleconsultation" OR teleconsult* OR **mobile* OR "webbased" OR "web-based" OR "Electronic Mail" OR "Electronic Mail" OR "e-mail" OR email*** OR "Mobile Application" OR "mobile apps" OR "mobile app" OR webapp* OR "SMS" OR "Cell Phones" OR "Smartphone" OR "Text Messaging" OR "Mobile Phone" OR "Cell Phone" OR "Smartphones" OR iphon* OR "text messag" OR "texting" OR **"mobile"** OR "cellular phone" OR "cellular phones" OR "smart phone" OR "telemedicine" OR "tele-care" OR "telecare" OR "tele-monitoring" OR "telemonitoring" OR **"website" OR "websites"** OR "personal digital assistant" OR "computer-assisted instruction" OR "ipad" OR ipad* OR "telenursing" OR telenurs* OR "virtual community" **OR "webpage" OR "webpages"** OR "web application" OR "web applications" OR "short message service" OR **"Internet" OR "internet" OR "online" OR "digital" OR digital*** OR "Reminder System" OR "Reminder Systems" OR "Reminder System" OR "Reminder Device" OR "Reminder Devices" OR "reminder messages" OR "reminder message" OR **"web"** OR "Virtual Reality" OR "Virtual Reality" OR "smart technology" OR "smart technol" OR "wearable technology" OR "wearable technologies" OR "telerehabilitation" OR "computer-assisted therapy" OR "computer-assisted therapy" OR "computer assisted therapy" OR "online therapy" OR "Computer Mediated Communication" OR "Computer Mediated Communications" OR **"remote" OR "on-line" OR "on line"** OR "wearable devices" OR "wearable device" OR "healthsensor" OR "healthsensors" OR "health sensor" OR "health sensors" OR "Robotics" OR "robotics" OR robot* OR "exergame" OR "exergames" OR exergam* OR "Video Game" OR "app-based" OR "wearable app" OR "wearable apps" OR "wearables" OR "Nintendo" OR "Wii" OR "gaming console" OR "gaming consoles"):ti)

(2) vier componenten in geval van usability

((("Rehabilitation" OR rehab* OR "Rehabilitation Nursing" OR "Rehabilitation Center" OR "Activities of Daily Living" OR "Animal Assisted Therapy" OR "Equine-Assisted Therapy" OR "Art Therapy" OR "Bibliotherapy" OR "Correction of Hearing Impairment" OR "Total Communication Methods" OR "Lipreading" OR "Manual Communication" OR "Dance Therapy" OR "Early Ambulation" OR "Exercise Therapy" OR "Endurance Training" OR "Continuous Passive Motion Therapy" OR "Muscle Stretching" OR "Plyometric Exercise" OR "Resistance Training" OR "Music Therapy" OR "Occupational Therapy" OR "Recreation Therapy" OR "Language Therapy" OR "Myofunctional Therapy" OR "Speech Therapy" OR "Alaryngeal Speech" OR "Voice Training" OR "Telerehabilitation" OR "Activity of Daily Living" OR "Plyometric Exercises" OR "Exercise" OR "exercise" OR "exercises" OR "physical activity"):ti AND ("elderly" OR "elder" OR "elders" OR geriatr* OR "Homes for the Aged" OR "Elderly Care" OR "Senior Centers" OR older person* OR old person* OR older patient* OR old patient* OR "older women" OR "old women" OR "older men" OR "old men" OR old adult* OR older adult* OR "Older individual" OR "Older individuals" OR "old people" OR "older people" OR "Oldest Old" OR "Nonagenarians" OR "Nonagenarian" OR "Octogenarians" OR "Octogenarian" OR "Centenarians" OR "Centenarian" OR "septuagenarian" OR "septuagenarians" OR "Aging" OR "aging" OR "ageing" OR "older population" OR "aging population" OR "aging population" OR geront* OR "old-aged" OR "old-age" OR "old aged" OR "old age"):ti,ab,kw AND ("accelerometers" OR "accelerometer" OR "accelerometry" OR acceleromet* OR "Telehealth" OR "web portals" OR "web portal" OR "web portal" OR "e-consult" OR econsult* OR telemed* OR "ehealth" OR "e-health" OR "mhealth" OR "m-health" OR "mobile health" OR "telehealth" OR "tele-health" OR "tele health" OR "**electronic communication"** OR "remote consultation" OR "remote health care" OR "remote healthcare" OR "remote care" OR "remote monitoring" OR "teleconsultation" OR teleconsult* OR **mobile* OR "webbased" OR "web-based" OR "Electronic Mail" OR "Electronic Mail" OR "e-mail" OR email*** OR "Mobile Application" OR "mobile apps" OR "mobile app" OR webapp* OR "SMS" OR "Cell Phones" OR "Smartphone" OR "Text Messaging" OR "Mobile Phone" OR "Cell Phone" OR "Smartphones" OR iphon* OR "text messag" OR "texting" OR **"mobile"** OR "cellular phone" OR "cellular phones" OR "smart phone" OR "telemedicine" OR "tele-care" OR "telecare" OR "tele-monitoring" OR "telemonitoring" OR **"website" OR "websites"** OR "personal digital assistant" OR "computer-assisted instruction" OR "ipad" OR ipad* OR "telenursing" OR telenurs* OR "virtual community" **OR "webpage" OR "webpages"** OR "web application" OR "web applications" OR "short message service" OR **"Internet" OR "internet" OR "online" OR "digital" OR digital*** OR "Reminder System" OR "Reminder Systems" OR "Reminder System" OR "Reminder Device" OR "Reminder Devices" OR "reminder messages" OR "reminder message" OR **"web"** OR "Virtual Reality" OR "Virtual Reality" OR "smart technology" OR "smart technol" OR "wearable technology" OR "wearable technologies" OR "telerehabilitation" OR "computer-assisted therapy" OR "computer-assisted therapy" OR "computer assisted therapy" OR "online therapy" OR "Computer Mediated Communication" OR "Computer Mediated Communications" OR **"remote" OR "on-line" OR "on line"** OR "wearable devices" OR "wearable device" OR "healthsensor" OR "healthsensors" OR "health sensor" OR "health sensors" OR "Robotics" OR "robotics" OR robot* OR "exergame" OR "exergames" OR exergam* OR

"Video Game" OR "app-based" OR "wearable app" OR "wearable apps" OR "wearables" OR "Nintendo" OR "Wii" OR "gaming console" OR "gaming consoles"):ti,ab,kw) **OR** (("Rehabilitation" OR rehab* OR "Rehabilitation Nursing" OR "Rehabilitation Center" OR "Activities of Daily Living" OR "Animal Assisted Therapy" OR "Equine-Assisted Therapy" OR "Art Therapy" OR "Bibliotherapy" OR "Correction of Hearing Impairment" OR "Total Communication Methods" OR "Lipreading" OR "Manual Communication" OR "Dance Therapy" OR "Early Ambulation" OR "Exercise Therapy" OR "Endurance Training" OR "Continuous Passive Motion Therapy" OR "Muscle Stretching" OR "Plyometric Exercise" OR "Resistance Training" OR "Music Therapy" OR "Occupational Therapy" OR "Recreation Therapy" OR "Language Therapy" OR "Myofunctional Therapy" OR "Speech Therapy" OR "Alaryngeal Speech" OR "Voice Training" OR "Telerehabilitation" OR "Activity of Daily Living" OR "Plyometric Exercises" OR "Exercise" OR "exercise" OR "exercises" OR "physical activity"):ti,ab,kw AND ("elderly" OR "elder" OR "elders" OR geriatr* OR "Homes for the Aged" OR "Elderly Care" OR "Senior Centers" OR older person* OR old person* OR older patient* OR old patient* OR "older women" OR "old women" OR "older men" OR "old men" OR old adult* OR older adult* OR "Older individual" OR "Older individuals" OR "old people" OR "older people" OR "Oldest Old" OR "Nonagenarians" OR "Nonagenarian" OR "Octogenarians" OR "Octogenarian" OR "Centenarians" OR "Centenarian" OR "septuagenarian" OR "septuagenarians" OR "Aging" OR "aging" OR "ageing" OR "older population" OR "aging population" OR "aging population" OR geront* OR "old-aged" OR "old-age" OR "old aged" OR "old age"):ti,ab,kw AND ("accelerometers" OR "accelerometer" OR "accelerometry" OR acceleromet* OR "Telehealth" OR "web portals" OR "web portal" OR "web portal" OR "e-consult" OR econsult* OR telemed* OR "ehealth" OR "e-health" OR "mhealth" OR "m-health" OR "mobile health" OR "telehealth" OR "tele-health" OR "tele health" OR "**electronic communication"** OR "remote consultation" OR "remote health care" OR "remote healthcare" OR "remote care" OR "remote monitoring" OR "teleconsultation" OR teleconsult* OR **mobile* OR "webbased" OR "web-based" OR "Electronic Mail" OR "Electronic Mail" OR "e-mail" OR email*** OR "Mobile Application" OR "mobile apps" OR "mobile app" OR webapp* OR "SMS" OR "Cell Phones" OR "Smartphone" OR "Text Messaging" OR "Mobile Phone" OR "Cell Phone" OR "Smartphones" OR iphon* OR "text messag" OR "texting" OR **"mobile"** OR "cellular phone" OR "cellular phones" OR "smart phone" OR "telemedicine" OR "tele-care" OR "telecare" OR "tele-monitoring" OR "telemonitoring" OR **"website" OR "websites"** OR "personal digital assistant" OR "computer-assisted instruction" OR "ipad" OR ipad* OR "telenursing" OR telenurs* OR "virtual community" **OR "webpage" OR "webpages"** OR "web application" OR "web applications" OR "short message service" OR **"Internet" OR "internet" OR "online" OR "digital" OR digital*** OR "Reminder System" OR "Reminder Systems" OR "Reminder System" OR "Reminder Device" OR "Reminder Devices" OR "reminder messages" OR "reminder message" OR **"web"** OR "Virtual Reality" OR "Virtual Reality" OR "smart technology" OR "smart technol" OR "wearable technology" OR "wearable technologies" OR "telerehabilitation" OR "computer-assisted therapy" OR "computer-assisted therapy" OR "computer assisted therapy" OR "online therapy" OR "Computer Mediated Communication" OR "Computer Mediated Communications" OR **"remote" OR "on-line" OR "on line"** OR "wearable devices" OR "wearable device"

OR "healthsensor" OR "healthsensors" OR "health sensor" OR "health sensors" OR "Robotics" OR "robotics" OR robot* OR "exergame" OR "exergames" OR exergam* OR "Video Game" OR "app-based" OR "wearable app" OR "wearable apps" OR "wearables" OR "Nintendo" OR "Wii" OR "gaming console" OR "gaming consoles"):ti)**)** AND ("usability" OR "Meaningful Use" OR "User experience" OR "User experiences" OR "usefulness"):ti,ab,kw

(3) Een nieuwe zoekstring met een omschrijving van de term feasibility. Ook weer met de nadruk op alle vier de componenten

("feasibility" OR "feasible"):ti,ab,kw AND (("Rehabilitation" OR rehab* OR "Rehabilitation Nursing" OR "Rehabilitation Center" OR "Activities of Daily Living" OR "Animal Assisted Therapy" OR "Equine-Assisted Therapy" OR "Art Therapy" OR "Bibliotherapy" OR "Correction of Hearing Impairment" OR "Total Communication Methods" OR "Lipreading" OR "Manual Communication" OR "Dance Therapy" OR "Early Ambulation" OR "Exercise Therapy" OR "Endurance Training" OR "Continuous Passive Motion Therapy" OR "Muscle Stretching" OR "Plyometric Exercise" OR "Resistance Training" OR "Music Therapy" OR "Occupational Therapy" OR "Recreation Therapy" OR "Language Therapy" OR "Myofunctional Therapy" OR "Speech Therapy" OR "Alaryngeal Speech" OR "Voice Training" OR "Telerehabilitation" OR "Activity of Daily Living" OR "Plyometric Exercises" OR "Exercise" OR "exercise" OR "exercises" OR "physical activity"):ti AND ("elderly" OR "elder" OR "elders" OR geriatr* OR "Homes for the Aged" OR "Elderly Care" OR "Senior Centers" OR older person* OR old person* OR older patient* OR old patient* OR "older women" OR "old women" OR "older men" OR "old men" OR old adult* OR older adult* OR "Older individual" OR "Older individuals" OR "old people" OR "older people" OR "Oldest Old" OR "Nonagenarians" OR "Nonagenarian" OR "Octogenarians" OR "Octogenarian" OR "Centenarians" OR "Centenarian" OR "septuagenarian" OR "septuagenarians" OR "Aging" OR "aging" OR "ageing" OR "older population" OR "aging population" OR "aging population" OR geront* OR "old-aged" OR "old-age" OR "old aged" OR "old age"):ti AND ("accelerometers" OR "accelerometer" OR "accelerometry" OR acceleromet* OR "Telehealth" OR "web portals" OR "web portal" OR "web portal" OR "e-consult" OR econsult* OR telemed* OR "ehealth" OR "e-health" OR "mhealth" OR "m-health" OR "mobile health" OR "telehealth" OR "tele-health" OR "tele health" OR "**electronic communication"** OR "remote consultation" OR "remote health care" OR "remote healthcare" OR "remote care" OR "remote monitoring" OR "teleconsultation" OR teleconsult* OR **mobile* OR "webbased" OR "web-based" OR "Electronic Mail" OR "Electronic Mail" OR "e-mail" OR email*** OR "Mobile Application" OR "mobile apps" OR "mobile app" OR webapp* OR "SMS" OR "Cell Phones" OR "Smartphone" OR "Text Messaging" OR "Mobile Phone" OR "Cell Phone" OR "Smartphones" OR iphon* OR "text messag" OR "texting" OR **"mobile"** OR "cellular phone" OR "cellular phones" OR "smart phone" OR "telemedicine" OR "tele-care" OR "telecare" OR "tele-monitoring" OR "telemonitoring" OR **"website" OR "websites"** OR "personal digital assistant" OR "computer-assisted instruction" OR "ipad" OR ipad* OR "telenursing" OR telenurs* OR "virtual community" **OR "webpage" OR "webpages"** OR "web application" OR "web applications" OR "short message service" OR

"Internet" OR "internet" OR "online" OR "digital" OR digital* OR "Reminder System" OR "Reminder Systems" OR "Reminder System" OR "Reminder Device" OR "Reminder Devices" OR "reminder messages" OR "reminder message" OR **"web"** OR "Virtual Reality" OR "Virtual Reality" OR "smart technology" OR "smart technol" OR "wearable technology" OR "wearable technologies" OR "telerehabilitation" OR "computer-assisted therapy" OR "computer-assisted therapy" OR "computer assisted therapy" OR "online therapy" OR "Computer Mediated Communication" OR "Computer Mediated Communications" OR **"remote" OR "on-line" OR "on line"** OR "wearable devices" OR "wearable device" OR "healthsensor" OR "healthsensors" OR "health sensor" OR "health sensors" OR "Robotics" OR "robotics" OR robot* OR "exergame" OR "exergames" OR exergam* OR "Video Game" OR "app-based" OR "wearable app" OR "wearable apps" OR "wearables" OR "Nintendo" OR "Wii" OR "gaming console" OR "gaming consoles"):ti)

10-4-2019

B. Nadruk op Revalidatie en/óf E-health

(("Rehabilitation" OR rehab* OR "Rehabilitation Nursing" OR "Rehabilitation Center" OR "Activities of Daily Living" OR "Animal Assisted Therapy" OR "Equine-Assisted Therapy" OR "Art Therapy" OR "Bibliotherapy" OR "Correction of Hearing Impairment" OR "Total Communication Methods" OR "Lipreading" OR "Manual Communication" OR "Dance Therapy" OR "Early Ambulation" OR "Exercise Therapy" OR "Endurance Training" OR "Continuous Passive Motion Therapy" OR "Muscle Stretching" OR "Plyometric Exercise" OR "Resistance Training" OR "Music Therapy" OR "Occupational Therapy" OR "Recreation Therapy" OR "Language Therapy" OR "Myofunctional Therapy" OR "Speech Therapy" OR "Alaryngeal Speech" OR "Voice Training" OR "Telerehabilitation" OR "Activity of Daily Living" OR "Plyometric Exercises" OR "Exercise" OR "exercise" OR "exercises" OR "physical activity"):ti AND ("elderly" OR "elder" OR "elders" OR geriatr* OR "Homes for the Aged" OR "Elderly Care" OR "Senior Centers" OR older person* OR old person* OR older patient* OR old patient* OR "older women" OR "old women" OR "older men" OR "old men" OR old adult* OR older adult* OR "Older individual" OR "Older individuals" OR "old people" OR "older people" OR "Oldest Old" OR "Nonagenarians" OR "Nonagenarian" OR "Octogenarians" OR "Octogenarian" OR "Centenarians" OR "Centenarian" OR "septuagenarian" OR "septuagenarians" OR "Aging" OR "aging" OR "ageing" OR "older population" OR "aging population" OR "aging population" OR geront* OR "old-aged" OR "old-age" OR "old aged" OR "old age"):ti,ab,kw AND ("accelerometers" OR "accelerometer" OR "accelerometry" OR acceleromet* OR "Telehealth" OR "web portals" OR "web portal" OR "web portal" OR "e-consult" OR econsult* OR telemed* OR "ehealth" OR "e-health" OR "mhealth" OR "m-health" OR "mobile health" OR "telehealth" OR "tele-health" OR "tele health" OR "**electronic communication"** OR "remote consultation" OR "remote health care" OR "remote healthcare" OR "remote care" OR "remote monitoring" OR "teleconsultation" OR teleconsult* OR **mobile* OR "webbased" OR "web-based" OR "Electronic Mail" OR "Electronic Mail" OR "e-**

mail" OR email* OR "Mobile Application" OR "mobile apps" OR "mobile app" OR webapp* OR "SMS" OR "Cell Phones" OR "Smartphone" OR "Text Messaging" OR "Mobile Phone" OR "Cell Phone" OR "Smartphones" OR iphon* OR "text messag" OR "texting" OR **"mobile"** OR "cellular phone" OR "cellular phones" OR "smart phone" OR "telemedicine" OR "tele-care" OR "telecare" OR "tele-monitoring" OR "telemonitoring" OR **"website" OR "websites"** OR "personal digital assistant" OR "computer-assisted instruction" OR "ipad" OR ipad* OR "telenursing" OR telenurs* OR "virtual community" **OR "webpage" OR "webpages"** OR "web application" OR "web applications" OR "short message service" OR **"Internet" OR "internet" OR "online" OR "digital" OR digital*** OR "Reminder System" OR "Reminder Systems" OR "Reminder System" OR "Reminder Device" OR "Reminder Devices" OR "reminder messages" OR "reminder message" OR **"web"** OR "Virtual Reality" OR "Virtual Reality" OR "smart technology" OR "smart technol" OR "wearable technology" OR "wearable technologies" OR "telerehabilitation" OR "computer-assisted therapy" OR "computer-assisted therapy" OR "computer assisted therapy" OR "online therapy" OR "Computer Mediated Communication" OR "Computer Mediated Communications" OR **"remote" OR "on-line" OR "on line"** OR "wearable devices" OR "wearable device" OR "healthsensor" OR "healthsensors" OR "health sensor" OR "health sensors" OR "Robotics" OR "robotics" OR robot* OR "exergame" OR "exergames" OR exergam* OR "Video Game" OR "app-based" OR "wearable app" OR "wearable apps" OR "wearables" OR "Nintendo" OR "Wii" OR "gaming console" OR "gaming consoles"):ti,ab,kw) OR (("Rehabilitation" OR rehab* OR "Rehabilitation Nursing" OR "Rehabilitation Center" OR "Activities of Daily Living" OR "Animal Assisted Therapy" OR "Equine-Assisted Therapy" OR "Art Therapy" OR "Bibliotherapy" OR "Correction of Hearing Impairment" OR "Total Communication Methods" OR "Lipreading" OR "Manual Communication" OR "Dance Therapy" OR "Early Ambulation" OR "Exercise Therapy" OR "Endurance Training" OR "Continuous Passive Motion Therapy" OR "Muscle Stretching" OR "Plyometric Exercise" OR "Resistance Training" OR "Music Therapy" OR "Occupational Therapy" OR "Recreation Therapy" OR "Language Therapy" OR "Myofunctional Therapy" OR "Speech Therapy" OR "Alaryngeal Speech" OR "Voice Training" OR "Telerehabilitation" OR "Activity of Daily Living" OR "Plyometric Exercises" OR "Exercise" OR "exercise" OR "exercises" OR "physical activity"):ti,ab,kw AND ("elderly" OR "elder" OR "elders" OR geriatr* OR "Homes for the Aged" OR "Elderly Care" OR "Senior Centers" OR older person* OR old person* OR older patient* OR old patient* OR "older women" OR "old women" OR "older men" OR "old men" OR old adult* OR older adult* OR "Older individual" OR "Older individuals" OR "old people" OR "older people" OR "Oldest Old" OR "Nonagenarians" OR "Nonagenarian" OR "Octogenarians" OR "Octogenarian" OR "Centenarians" OR "Centenarian" OR "septuagenarian" OR "septuagenarians" OR "Aging" OR "aging" OR "ageing" OR "older population" OR "aging population" OR "aging population" OR geront* OR "old-aged" OR "old-age" OR "old aged" OR "old age"):ti,ab,kw AND ("accelerometers" OR "accelerometer" OR "accelerometry" OR acceleromet* OR "Telehealth" OR "web portals" OR "web portal" OR "web portal" OR "e-consult" OR econsult* OR telemed* OR "ehealth" OR "e-health" OR "mhealth" OR "m-health" OR "mobile health" OR "telehealth" OR "tele-health" OR "tele health" OR "**electronic communication"** OR "remote consultation" OR "remote health care" OR "remote healthcare" OR "remote

care" OR "remote monitoring" OR "teleconsultation" OR teleconsult* OR **mobile* OR "webbased" OR "web-based" OR "Electronic Mail" OR "Electronic Mail" OR "e-mail" OR email*** OR "Mobile Application" OR "mobile apps" OR "mobile app" OR webapp* OR "SMS" OR "Cell Phones" OR "Smartphone" OR "Text Messaging" OR "Mobile Phone" OR "Cell Phone" OR "Smartphones" OR iphon* OR "text messag" OR "texting" OR **"mobile"** OR "cellular phone" OR "cellular phones" OR "smart phone" OR "telemedicine" OR "tele-care" OR "telecare" OR "tele-monitoring" OR "telemonitoring" OR **"website" OR "websites"** OR "personal digital assistant" OR "computer-assisted instruction" OR "ipad" OR ipad* OR "telenursing" OR telenurs* OR "virtual community" **OR "webpage" OR "webpages"** OR "web application" OR "web applications" OR "short message service" OR **"Internet" OR "internet" OR "online" OR "digital" OR digital*** OR "Reminder System" OR "Reminder Systems" OR "Reminder System" OR "Reminder Device" OR "Reminder Devices" OR "reminder messages" OR "reminder message" OR **"web"** OR "Virtual Reality" OR "Virtual Reality" OR "smart technology" OR "smart technol" OR "wearable technology" OR "wearable technologies" OR "telerehabilitation" OR "computer-assisted therapy" OR "computer-assisted therapy" OR "computer assisted therapy" OR "online therapy" OR "Computer Mediated Communication" OR "Computer Mediated Communications" OR **"remote" OR "on-line" OR "on line"** OR "wearable devices" OR "wearable device" OR "healthsensor" OR "healthsensors" OR "health sensor" OR "health sensors" OR "Robotics" OR "robotics" OR robot* OR "exergame" OR "exergames" OR exergam* OR "Video Game" OR "app-based" OR "wearable app" OR "wearable apps" OR "wearables" OR "Nintendo" OR "Wii" OR "gaming console" OR "gaming consoles"):ti)

B.2 - Nadruk op Revalidatie en/óf E-health – usability

((("Rehabilitation" OR rehab* OR "Rehabilitation Nursing" OR "Rehabilitation Center" OR "Activities of Daily Living" OR "Animal Assisted Therapy" OR "Equine-Assisted Therapy" OR "Art Therapy" OR "Bibliotherapy" OR "Correction of Hearing Impairment" OR "Total Communication Methods" OR "Lipreading" OR "Manual Communication" OR "Dance Therapy" OR "Early Ambulation" OR "Exercise Therapy" OR "Endurance Training" OR "Continuous Passive Motion Therapy" OR "Muscle Stretching" OR "Plyometric Exercise" OR "Resistance Training" OR "Music Therapy" OR "Occupational Therapy" OR "Recreation Therapy" OR "Language Therapy" OR "Myofunctional Therapy" OR "Speech Therapy" OR "Alaryngeal Speech" OR "Voice Training" OR "Telerehabilitation" OR "Activity of Daily Living" OR "Plyometric Exercises" OR "Exercise" OR "exercise" OR "exercises" OR "physical activity"):ti AND ("elderly" OR "elder" OR "elders" OR geriatr* OR "Homes for the Aged" OR "Elderly Care" OR "Senior Centers" OR older person* OR old person* OR older patient* OR old patient* OR "older women" OR "old women" OR "older men" OR "old men" OR old adult* OR older adult* OR "Older individual" OR "Older individuals" OR "old people" OR "older people" OR "Oldest Old" OR "Nonagenarians" OR "Nonagenarian" OR "Octogenarians" OR "Octogenarian" OR "Centenarians" OR "Centenarian" OR "septuagenarian" OR "septuagenarians" OR "Aging" OR "aging" OR "ageing" OR "older population" OR "aging population" OR "aging population" OR geront* OR "old-aged" OR "old-age" OR "old aged" OR "old age"):ti,ab,kw AND ("accelerometers" OR

"accelerometer" OR "accelerometry" OR acceleromet* OR "Telehealth" OR "web portals" OR "web portal" OR "web portal" OR "e-consult" OR econsult* OR telemed* OR "ehealth" OR "e-health" OR "mhealth" OR "m-health" OR "mobile health" OR "telehealth" OR "tele-health" OR "tele health" OR "**electronic communication"** OR "remote consultation" OR "remote health care" OR "remote healthcare" OR "remote care" OR "remote monitoring" OR "teleconsultation" OR teleconsult* OR **mobile* OR "webbased" OR "web-based" OR "Electronic Mail" OR "Electronic Mail" OR "e-mail" OR email*** OR "Mobile Application" OR "mobile apps" OR "mobile app" OR webapp* OR "SMS" OR "Cell Phones" OR "Smartphone" OR "Text Messaging" OR "Mobile Phone" OR "Cell Phone" OR "Smartphones" OR iphon* OR "text messag" OR "texting" OR **"mobile"** OR "cellular phone" OR "cellular phones" OR "smart phone" OR "telemedicine" OR "tele-care" OR "telecare" OR "tele-monitoring" OR "telemonitoring" OR **"website" OR "websites"** OR "personal digital assistant" OR "computer-assisted instruction" OR "ipad" OR ipad* OR "telenursing" OR telenurs* OR "virtual community" **OR "webpage" OR "webpages"** OR "web application" OR "web applications" OR "short message service" OR **"Internet" OR "internet" OR "online" OR "digital" OR digital*** OR "Reminder System" OR "Reminder Systems" OR "Reminder System" OR "Reminder Device" OR "Reminder Devices" OR "reminder messages" OR "reminder message" OR **"web"** OR "Virtual Reality" OR "Virtual Reality" OR "smart technology" OR "smart technol" OR "wearable technology" OR "wearable technologies" OR "telerehabilitation" OR "computer-assisted therapy" OR "computer-assisted therapy" OR "computer assisted therapy" OR "online therapy" OR "Computer Mediated Communication" OR "Computer Mediated Communications" OR **"remote" OR "on-line" OR "on line"** OR "wearable devices" OR "wearable device" OR "healthsensor" OR "healthsensors" OR "health sensor" OR "health sensors" OR "Robotics" OR "robotics" OR robot* OR "exergame" OR "exergames" OR exergam* OR "Video Game" OR "app-based" OR "wearable app" OR "wearable apps" OR "wearables" OR "Nintendo" OR "Wii" OR "gaming console" OR "gaming consoles"):ti,ab,kw) **OR** (("Rehabilitation" OR rehab* OR "Rehabilitation Nursing" OR "Rehabilitation Center" OR "Activities of Daily Living" OR "Animal Assisted Therapy" OR "Equine-Assisted Therapy" OR "Art Therapy" OR "Bibliotherapy" OR "Correction of Hearing Impairment" OR "Total Communication Methods" OR "Lipreading" OR "Manual Communication" OR "Dance Therapy" OR "Early Ambulation" OR "Exercise Therapy" OR "Endurance Training" OR "Continuous Passive Motion Therapy" OR "Muscle Stretching" OR "Plyometric Exercise" OR "Resistance Training" OR "Music Therapy" OR "Occupational Therapy" OR "Recreation Therapy" OR "Language Therapy" OR "Myofunctional Therapy" OR "Speech Therapy" OR "Alaryngeal Speech" OR "Voice Training" OR "Telerehabilitation" OR "Activity of Daily Living" OR "Plyometric Exercises" OR "Exercise" OR "exercise" OR "exercises" OR "physical activity"):ti,ab,kw AND ("elderly" OR "elder" OR "elders" OR geriatr* OR "Homes for the Aged" OR "Elderly Care" OR "Senior Centers" OR older person* OR old person* OR older patient* OR old patient* OR "older women" OR "old women" OR "older men" OR "old men" OR old adult* OR older adult* OR "Older individual" OR "Older individuals" OR "old people" OR "older people" OR "Oldest Old" OR "Nonagenarians" OR "Nonagenarian" OR "Octogenarians" OR "Octogenarian" OR "Centenarians" OR "Centenarian" OR "septuagenarian" OR "septuagenarians" OR "Aging" OR "aging" OR "ageing" OR "older

population" OR "aging population" OR "aging population" OR geront* OR "old-aged" OR "old-age" OR "old aged" OR "old age"):ti,ab,kw AND ("accelerometers" OR "accelerometer" OR "accelerometry" OR acceleromet* OR "Telehealth" OR "web portals" OR "web portal" OR "web portal" OR "e-consult" OR econsult* OR telemed* OR "ehealth" OR "e-health" OR "mhealth" OR "m-health" OR "mobile health" OR "telehealth" OR "tele-health" OR "tele health" OR "**electronic communication"** OR "remote consultation" OR "remote health care" OR "remote healthcare" OR "remote care" OR "remote monitoring" OR "teleconsultation" OR teleconsult* OR **mobile* OR "webbased" OR "web-based" OR "Electronic Mail" OR "Electronic Mail" OR "e-mail" OR email*** OR "Mobile Application" OR "mobile apps" OR "mobile app" OR webapp* OR "SMS" OR "Cell Phones" OR "Smartphone" OR "Text Messaging" OR "Mobile Phone" OR "Cell Phone" OR "Smartphones" OR iphon* OR "text messag" OR "texting" OR **"mobile"** OR "cellular phone" OR "cellular phones" OR "smart phone" OR "telemedicine" OR "tele-care" OR "telecare" OR "tele-monitoring" OR "telemonitoring" OR **"website" OR "websites"** OR "personal digital assistant" OR "computer-assisted instruction" OR "ipad" OR ipad* OR "telenursing" OR telenurs* OR "virtual community" **OR "webpage" OR "webpages"** OR "web application" OR "web applications" OR "short message service" OR **"Internet" OR "internet" OR "online" OR "digital" OR digital*** OR "Reminder System" OR "Reminder Systems" OR "Reminder System" OR "Reminder Device" OR "Reminder Devices" OR "reminder messages" OR "reminder message" OR **"web"** OR "Virtual Reality" OR "Virtual Reality" OR "smart technology" OR "smart technol" OR "wearable technology" OR "wearable technologies" OR "telerehabilitation" OR "computer-assisted therapy" OR "computer-assisted therapy" OR "computer assisted therapy" OR "online therapy" OR "Computer Mediated Communication" OR "Computer Mediated Communications" OR **"remote" OR "on-line" OR "on line"** OR "wearable devices" OR "wearable device" OR "healthsensor" OR "healthsensors" OR "health sensor" OR "health sensors" OR "Robotics" OR "robotics" OR robot* OR "exergame" OR "exergames" OR exergam* OR "Video Game" OR "app-based" OR "wearable app" OR "wearable apps" OR "wearables" OR "Nintendo" OR "Wii" OR "gaming console" OR "gaming consoles"):ti)**)** AND ("usability" OR "Meaningful Use" OR "User experience" OR "User experiences" OR "usefulness"):ti,ab,kw

Emcare
http://ovidsp.ovid.com/ovidweb.cgi?T=JS&NEWS=n&CSC=Y&PAGE=main&D=emcr

8-1-2021

1.

((exp *"Rehabilitation"/ OR rehab*.ti OR exp *"Rehabilitation Nursing"/ OR *"Rehabilitation Center"/ OR "Activities of Daily Living".ti OR "Animal Assisted Therapy".ti OR "Equine-Assisted Therapy".ti OR "Art Therapy".ti OR "Bibliotherapy".ti OR "Correction of Hearing Impairment".ti OR "Total Communication Methods".ti OR "Lipreading".ti OR "Manual Communication".ti OR "Dance Therapy".ti OR "Early

Ambulation".ti OR "Exercise Therapy".ti OR "Endurance Training".ti OR "Continuous Passive Motion Therapy".ti OR "Muscle Stretching".ti OR "Plyometric Exercise".ti OR "Resistance Training".ti OR "Music Therapy".ti OR "Occupational Therapy".ti OR "Recreation Therapy".ti OR "Language Therapy".ti OR "Myofunctional Therapy".ti OR "Speech Therapy".ti OR "Alaryngeal Speech".ti OR "Voice Training".ti OR "Telerehabilitation".ti OR "Activity of Daily Living".ti OR "Plyometric Exercises".ti OR exp *"Exercise"/ OR "exercise".ti OR "exercises".ti OR "physical activity".ti) AND (exp "Aged"/ OR "elderly".ti OR "elder".ti OR "elders".ti OR geriatr*.ti OR exp *"Homes for the Aged"/ OR exp *"Elderly Care"/ OR exp *"Senior Centers"/ OR older person*.ti OR old person*.ti OR older patient*.ti OR old patient*.ti OR "older women".ti OR "old women".ti OR "older men".ti OR "old men".ti OR old adult*.ti OR older adult*.ti OR "Older individual".ti OR "Older individuals".ti OR "old people".ti OR "older people".ti OR "Oldest Old".ti OR "Nonagenarians".ti OR "Nonagenarian".ti OR "Octogenarians".ti OR "Octogenarian".ti OR "Centenarians".ti OR "Centenarian".ti OR "septuagenarian".ti OR "septuagenarians".ti OR exp *"Aging"/ OR "aging".ti OR "ageing".ti OR "older population".ti OR "aging population".ti OR "aging population".ti OR geront*.ti OR "old-aged".ti OR "old-age".ti OR "old aged".ti OR "old age".ti) AND ("accelerometers".ti OR "accelerometer".ti OR "accelerometry".ti OR acceleromet*.ti OR exp *"Telehealth"/ OR "web portals".ti OR "web portal".ti OR "web portal*".ti OR "e-consult*".ti OR econsult*.ti OR telemed*.ti OR "ehealth".ti OR "e-health".ti OR "mhealth".ti OR "m-health".ti OR "mobile health".ti OR "telehealth".ti OR "tele-health".ti OR "tele health".ti OR "**electronic communication*".ti** OR "remote consultation".ti OR "remote health care".ti OR "remote healthcare".ti OR "remote care".ti OR "remote monitoring".ti OR "teleconsultation".ti OR teleconsult*.ti OR **mobile*.ti OR "webbased".ti OR "web-based".ti OR exp *"Electronic Mail"/ OR "Electronic Mail".ti OR "e-mail*".ti OR email*.ti** OR exp *"Mobile Application"/ OR "mobile apps".ti OR "mobile app".ti OR webapp*.ti OR "SMS".ti OR "Cell Phones".ti OR "Smartphone".ti OR "Text Messaging".ti OR exp *"Mobile Phone"/ OR "Cell Phone".ti OR "Smartphones".ti OR iphon*.ti OR "text messag*".ti OR "texting".ti OR **"mobile".ti** OR "cellular phone".ti OR "cellular phones".ti OR "smart phone".ti OR "telemedicine".ti OR "tele-care".ti OR "telecare".ti OR "tele-monitoring".ti OR "telemonitoring".ti OR **"website".ti OR "websites".ti** OR "personal digital assistant".ti OR "computer-assisted instruction".ti OR "ipad".ti OR ipad*.ti OR "telenursing".ti OR telenurs*.ti OR "virtual community".ti **OR "webpage".ti OR "webpages".ti** OR "web application".ti OR "web applications".ti OR "short message service".ti OR **exp *"Internet"/ OR "internet".ti OR "online".ti OR "digital".ti OR digital*.ti** OR exp *"Reminder System"/ OR "Reminder Systems".ti OR "Reminder System".ti OR "Reminder Device".ti OR "Reminder Devices".ti OR "reminder messages".ti OR "reminder message".ti OR **"web".ti** OR exp *"Virtual Reality"/ OR "Virtual Reality".ti OR "smart technology".ti OR "smart technol*".ti OR "wearable technology".ti OR "wearable technologies".ti OR "telerehabilitation".ti OR "computer-assisted therapy"/ OR "computer-assisted therapy".ti OR "computer assisted therapy".ti OR "online therapy".ti OR "Computer Mediated Communication".ti OR "Computer Mediated Communications".ti OR **"remote".ti OR "on-line".ti OR "on line".ti** OR "wearable devices".ti OR "wearable device".ti OR "healthsensor".ti OR "healthsensors".ti OR "health sensor".ti OR "health sensors".ti OR exp *"Robotics"/ OR "robotics".ti OR robot*.ti OR "exergame".ti OR "exergames".ti OR exergam*.ti OR exp

*"Video Game"/ OR "app-based".ti OR "wearable app".ti OR "wearable apps".ti OR "wearables".ti OR "Nintendo".ti OR "Wii".ti OR "gaming console".ti OR "gaming consoles".ti)) NOT (conference review or conference abstract).pt

(2) vier componenten in geval van usability

(((exp *"Rehabilitation"/ OR rehab*.ti OR exp *"Rehabilitation Nursing"/ OR *"Rehabilitation Center"/ OR "Activities of Daily Living".ti OR "Animal Assisted Therapy".ti OR "Equine-Assisted Therapy".ti OR "Art Therapy".ti OR "Bibliotherapy".ti OR "Correction of Hearing Impairment".ti OR "Total Communication Methods".ti OR "Lipreading".ti OR "Manual Communication".ti OR "Dance Therapy".ti OR "Early Ambulation".ti OR "Exercise Therapy".ti OR "Endurance Training".ti OR "Continuous Passive Motion Therapy".ti OR "Muscle Stretching".ti OR "Plyometric Exercise".ti OR "Resistance Training".ti OR "Music Therapy".ti OR "Occupational Therapy".ti OR "Recreation Therapy".ti OR "Language Therapy".ti OR "Myofunctional Therapy".ti OR "Speech Therapy".ti OR "Alaryngeal Speech".ti OR "Voice Training".ti OR "Telerehabilitation".ti OR "Activity of Daily Living".ti OR "Plyometric Exercises".ti OR exp *"Exercise"/ OR "exercise".ti OR "exercises".ti OR "physical activity".ti) AND (exp "Aged"/ OR "elderly".ti OR "elder".ti OR "elders".ti OR geriatr*.ti OR exp *"Homes for the Aged"/ OR exp *"Elderly Care"/ OR exp *"Senior Centers"/ OR older person*.ti OR old person*.ti OR older patient*.ti OR old patient*.ti OR "older women".ti OR "old women".ti OR "older men".ti OR "old men".ti OR old adult*.ti OR older adult*.ti OR "Older individual".ti OR "Older individuals".ti OR "old people".ti OR "older people".ti OR "Oldest Old".ti OR "Nonagenarians".ti OR "Nonagenarian".ti OR "Octogenarians".ti OR "Octogenarian".ti OR "Centenarians".ti OR "Centenarian".ti OR "septuagenarian".ti OR "septuagenarians".ti OR exp *"Aging"/ OR "aging".ti OR "ageing".ti OR "older population".ti OR "aging population".ti OR "aging population".ti OR geront*.ti OR "old-aged".ti OR "old-age".ti OR "old aged".ti OR "old age".ti) AND ("accelerometers".ti,ab OR "accelerometer".ti,ab OR "accelerometry".ti,ab OR acceleromet*.ti,ab OR exp *"Telehealth"/ OR "web portals".ti,ab OR "web portal".ti,ab OR "web portal*".ti,ab OR "e-consult*".ti,ab OR econsult*.ti,ab OR telemed*.ti,ab OR "ehealth".ti,ab OR "e-health".ti,ab OR "mhealth".ti,ab OR "m-health".ti,ab OR "mobile health".ti,ab OR "telehealth".ti,ab OR "tele-health".ti,ab OR "tele health".ti,ab OR "**electronic communication*".ti** OR "remote consultation".ti,ab OR "remote health care".ti,ab OR "remote healthcare".ti,ab OR "remote care".ti,ab OR "remote monitoring".ti,ab OR "teleconsultation".ti,ab OR teleconsult*.ti,ab OR **mobile*.ti OR "webbased".ti OR "web-based".ti OR exp *"Electronic Mail"/ OR "Electronic Mail".ti OR "e-mail*".ti OR email*.ti** OR exp *"Mobile Application"/ OR "mobile apps".ti,ab OR "mobile app".ti,ab OR webapp*.ti,ab OR "SMS".ti,ab OR "Cell Phones".ti,ab OR "Smartphone".ti,ab OR "Text Messaging".ti,ab OR exp *"Mobile Phone"/ OR "Cell Phone".ti,ab OR "Smartphones".ti,ab OR iphon*.ti,ab OR "text messag*".ti,ab OR "texting".ti,ab OR **"mobile".ti** OR "cellular phone".ti,ab OR "cellular phones".ti,ab OR "smart phone".ti,ab OR "telemedicine".ti,ab OR "tele-care".ti,ab OR "telecare".ti,ab OR "tele-monitoring".ti,ab OR "telemonitoring".ti,ab OR **"website".ti OR "websites".ti** OR "personal digital assistant".ti,ab OR "computer-assisted instruction".ti,ab OR "ipad".ti,ab

OR ipad*.ti,ab OR "telenursing".ti,ab OR telenurs*.ti,ab OR "virtual community".ti,ab **OR "webpage".ti OR "webpages".ti** OR "web application".ti,ab OR "web applications".ti,ab OR "short message service".ti,ab OR **exp *"Internet"/ OR "internet".ti OR "online".ti OR "digital".ti OR digital*.ti** OR exp *"Reminder System"/ OR "Reminder Systems".ti,ab OR "Reminder System".ti,ab OR "Reminder Device".ti,ab OR "Reminder Devices".ti,ab OR "reminder messages".ti,ab OR "reminder message".ti,ab OR **"web".ti** OR exp *"Virtual Reality"/ OR "Virtual Reality".ti,ab OR "smart technology".ti,ab OR "smart technol*".ti,ab OR "wearable technology".ti,ab OR "wearable technologies".ti,ab OR "telerehabilitation".ti,ab OR *"computer-assisted therapy"/ OR "computer-assisted therapy".ti,ab OR "computer assisted therapy".ti,ab OR "online therapy".ti,ab OR "Computer Mediated Communication".ti,ab OR "Computer Mediated Communications".ti,ab OR **"remote".ti OR "on-line".ti OR "on line".ti** OR "wearable devices".ti,ab OR "wearable device".ti,ab OR "healthsensor".ti,ab OR "healthsensors".ti,ab OR "health sensor".ti,ab OR "health sensors".ti,ab OR exp *"Robotics"/ OR "robotics".ti,ab OR robot*.ti,ab OR "exergame".ti,ab OR "exergames".ti,ab OR exergam*.ti,ab OR exp *"Video Game"/ OR "app-based".ti,ab OR "wearable app".ti,ab OR "wearable apps".ti,ab OR "wearables".ti,ab OR "Nintendo".ti,ab OR "Wii".ti,ab OR "gaming console".ti,ab OR "gaming consoles".ti,ab)) **OR** ((exp *"Rehabilitation"/ OR rehab*.ti,ab OR exp *"Rehabilitation Nursing"/ OR exp *"Rehabilitation Center"/ OR "Activities of Daily Living".ti,ab OR "Animal Assisted Therapy".ti,ab OR "Equine-Assisted Therapy".ti,ab OR "Art Therapy".ti,ab OR "Bibliotherapy".ti,ab OR "Correction of Hearing Impairment".ti,ab OR "Total Communication Methods".ti,ab OR "Lipreading".ti,ab OR "Manual Communication".ti,ab OR "Dance Therapy".ti,ab OR "Early Ambulation".ti,ab OR "Exercise Therapy".ti,ab OR "Endurance Training".ti,ab OR "Continuous Passive Motion Therapy".ti,ab OR "Muscle Stretching".ti,ab OR "Plyometric Exercise".ti,ab OR "Resistance Training".ti,ab OR "Music Therapy".ti,ab OR "Occupational Therapy".ti,ab OR "Recreation Therapy".ti,ab OR "Language Therapy".ti,ab OR "Myofunctional Therapy".ti,ab OR "Speech Therapy".ti,ab OR "Alaryngeal Speech".ti,ab OR "Voice Training".ti,ab OR "Telerehabilitation".ti,ab OR "Activity of Daily Living".ti,ab OR "Plyometric Exercises".ti,ab OR exp *"Exercise"/ OR "exercise".ti,ab OR "exercises".ti,ab OR "physical activity".ti,ab) AND (exp "Aged"/ OR "elderly".ti OR "elder".ti OR "elders".ti OR geriatr*.ti OR exp "Homes for the Aged"/ OR exp "Elderly Care"/ OR exp "Senior Centers"/ OR older person*.ti OR old person*.ti OR older patient*.ti OR old patient*.ti OR "older women".ti OR "old women".ti OR "older men".ti OR "old men".ti OR old adult*.ti OR older adult*.ti OR "Older individual".ti OR "Older individuals".ti OR "old people".ti OR "older people".ti OR "Oldest Old".ti OR "Nonagenarians".ti OR "Nonagenarian".ti OR "Octogenarians".ti OR "Octogenarian".ti OR "Centenarians".ti OR "Centenarian".ti OR "septuagenarian".ti OR "septuagenarians".ti OR exp "Aging"/ OR "aging".ti OR "ageing".ti OR "older population".ti OR "aging population".ti OR "aging population".ti OR geront*.ti OR "old-aged".ti OR "old-age".ti OR "old aged".ti OR "old age".ti) AND ("accelerometers".ti,ab OR "accelerometer".ti,ab OR "accelerometry".ti,ab OR acceleromet*.ti,ab OR exp *"Telehealth"/ OR "web portals".ti,ab OR "web portal".ti,ab OR "web portal*".ti,ab OR "e-consult*".ti,ab OR econsult*.ti,ab OR telemed*.ti,ab OR "ehealth".ti,ab OR "e-health".ti,ab OR "mhealth".ti,ab OR "m-health".ti,ab OR "mobile health".ti,ab OR

"telehealth".ti,ab OR "tele-health".ti,ab OR "tele health".ti,ab OR "**electronic communication*".ti** OR "remote consultation".ti,ab OR "remote health care".ti,ab OR "remote healthcare".ti,ab OR "remote care".ti,ab OR "remote monitoring".ti,ab OR "teleconsultation".ti,ab OR teleconsult*.ti,ab OR **mobile*.ti OR "webbased".ti OR "web-based".ti OR exp *"Electronic Mail"/ OR "Electronic Mail".ti OR "e-mail*".ti OR email*.ti** OR exp *"Mobile Application"/ OR "mobile apps".ti,ab OR "mobile app".ti,ab OR webapp*.ti,ab OR "SMS".ti,ab OR "Cell Phones".ti,ab OR "Smartphone".ti,ab OR "Text Messaging".ti,ab OR exp *"Mobile Phone"/ OR "Cell Phone".ti,ab OR "Smartphones".ti,ab OR iphon*.ti,ab OR "text messag*".ti,ab OR "texting".ti,ab OR **"mobile".ti** OR "cellular phone".ti,ab OR "cellular phones".ti,ab OR "smart phone".ti,ab OR "telemedicine".ti,ab OR "tele-care".ti,ab OR "telecare".ti,ab OR "tele-monitoring".ti,ab OR "telemonitoring".ti,ab OR **"website".ti OR "websites".ti** OR "personal digital assistant".ti,ab OR "computer-assisted instruction".ti,ab OR "ipad".ti,ab OR ipad*.ti,ab OR "telenursing".ti,ab OR telenurs*.ti,ab OR "virtual community".ti,ab **OR "webpage".ti OR "webpages".ti** OR "web application".ti,ab OR "web applications".ti,ab OR "short message service".ti,ab OR **exp *"Internet"/ OR "internet".ti OR "online".ti OR "digital".ti OR digital*.ti** OR exp *"Reminder System"/ OR "Reminder Systems".ti,ab OR "Reminder System".ti,ab OR "Reminder Device".ti,ab OR "Reminder Devices".ti,ab OR "reminder messages".ti,ab OR "reminder message".ti,ab OR **"web".ti** OR exp *"Virtual Reality"/ OR "Virtual Reality".ti,ab OR "smart technology".ti,ab OR "smart technol*".ti,ab OR "wearable technology".ti,ab OR "wearable technologies".ti,ab OR "telerehabilitation".ti,ab OR "computer-assisted therapy"/ OR "computer-assisted therapy".ti,ab OR "computer assisted therapy".ti,ab OR "online therapy".ti,ab OR "Computer Mediated Communication".ti,ab OR "Computer Mediated Communications".ti,ab OR **"remote".ti OR "on-line".ti OR "on line".ti** OR "wearable devices".ti,ab OR "wearable device".ti,ab OR "healthsensor".ti,ab OR "healthsensors".ti,ab OR "health sensor".ti,ab OR "health sensors".ti,ab OR exp *"Robotics"/ OR "robotics".ti,ab OR robot*.ti,ab OR "exergame".ti,ab OR "exergames".ti,ab OR exergam*.ti,ab OR exp *"Video Game"/ OR "app-based".ti,ab OR "wearable app".ti,ab OR "wearable apps".ti,ab OR "wearables".ti,ab OR "Nintendo".ti,ab OR "Wii".ti,ab OR "gaming console".ti,ab OR "gaming consoles".ti,ab))**)** NOT (conference review or conference abstract).pt AND ("usability".mp OR "Meaningful Use".mp OR "User experience".mp OR "User experiences".mp OR "usefulness".mp)

(3) Een nieuwe zoekstring met een omschrijving van de term feasibility. Ook weer met de nadruk op alle vier de componenten

("feasibility".mp OR "feasible".mp OR "Feasibility Study"/ OR "program feasibility"/) AND ((exp *"Rehabilitation"/ OR rehab*.ti OR exp *"Rehabilitation Nursing"/ OR *"Rehabilitation Center"/ OR "Activities of Daily Living".ti OR "Animal Assisted Therapy".ti OR "Equine-Assisted Therapy".ti OR "Art Therapy".ti OR "Bibliotherapy".ti OR "Correction of Hearing Impairment".ti OR "Total Communication Methods".ti OR "Lipreading".ti OR "Manual Communication".ti OR "Dance Therapy".ti OR "Early Ambulation".ti OR "Exercise Therapy".ti OR "Endurance Training".ti OR "Continuous Passive Motion Therapy".ti OR "Muscle Stretching".ti OR "Plyometric Exercise".ti OR "Resistance Training".ti OR "Music Therapy".ti OR "Occupational Therapy".ti OR

"Recreation Therapy".ti OR "Language Therapy".ti OR "Myofunctional Therapy".ti OR "Speech Therapy".ti OR "Alaryngeal Speech".ti OR "Voice Training".ti OR "Telerehabilitation".ti OR "Activity of Daily Living".ti OR "Plyometric Exercises".ti OR exp *"Exercise"/ OR "exercise".ti OR "exercises".ti OR "physical activity".ti) AND (exp "Aged"/ OR "elderly".ti OR "elder".ti OR "elders".ti OR geriatr*.ti OR exp *"Homes for the Aged"/ OR exp *"Elderly Care"/ OR exp *"Senior Centers"/ OR older person*.ti OR old person*.ti OR older patient*.ti OR old patient*.ti OR "older women".ti OR "old women".ti OR "older men".ti OR "old men".ti OR old adult*.ti OR older adult*.ti OR "Older individual".ti OR "Older individuals".ti OR "old people".ti OR "older people".ti OR "Oldest Old".ti OR "Nonagenarians".ti OR "Nonagenarian".ti OR "Octogenarians".ti OR "Octogenarian".ti OR "Centenarians".ti OR "Centenarian".ti OR "septuagenarian".ti OR "septuagenarians".ti OR exp *"Aging"/ OR "aging".ti OR "ageing".ti OR "older population".ti OR "aging population".ti OR "aging population".ti OR geront*.ti OR "old-aged".ti OR "old-age".ti OR "old aged".ti OR "old age".ti) AND ("accelerometers".ti OR "accelerometer".ti OR "accelerometry".ti OR acceleromet*.ti OR exp *"Telehealth"/ OR "web portals".ti OR "web portal".ti OR "web portal*".ti OR "e-consult*".ti OR econsult*.ti OR telemed*.ti OR "ehealth".ti OR "e-health".ti OR "mhealth".ti OR "m-health".ti OR "mobile health".ti OR "telehealth".ti OR "tele-health".ti OR "tele health".ti OR "**electronic communication*".ti** OR "remote consultation".ti OR "remote health care".ti OR "remote healthcare".ti OR "remote care".ti OR "remote monitoring".ti OR "teleconsultation".ti OR teleconsult*.ti OR **mobile*.ti OR "webbased".ti OR "web-based".ti OR exp *"Electronic Mail"/ OR "Electronic Mail".ti OR "e-mail*".ti OR email*.ti** OR exp *"Mobile Application"/ OR "mobile apps".ti OR "mobile app".ti OR webapp*.ti OR "SMS".ti OR "Cell Phones".ti OR "Smartphone".ti OR "Text Messaging".ti OR exp *"Mobile Phone"/ OR "Cell Phone".ti OR "Smartphones".ti OR iphon*.ti OR "text messag*".ti OR "texting".ti OR **"mobile".ti** OR "cellular phone".ti OR "cellular phones".ti OR "smart phone".ti OR "telemedicine".ti OR "tele-care".ti OR "telecare".ti OR "tele-monitoring".ti OR "telemonitoring".ti OR **"website".ti OR "websites".ti** OR "personal digital assistant".ti OR "computer-assisted instruction".ti OR "ipad".ti OR ipad*.ti OR "telenursing".ti OR telenurs*.ti OR "virtual community".ti **OR "webpage".ti OR "webpages".ti** OR "web application".ti OR "web applications".ti OR "short message service".ti OR **exp *"Internet"/ OR "internet".ti OR "online".ti OR "digital".ti OR digital*.ti** OR exp *"Reminder System"/ OR "Reminder Systems".ti OR "Reminder System".ti OR "Reminder Device".ti OR "Reminder Devices".ti OR "reminder messages".ti OR "reminder message".ti OR **"web".ti** OR exp *"Virtual Reality"/ OR "Virtual Reality".ti OR "smart technology".ti OR "smart technol*".ti OR "wearable technology".ti OR "wearable technologies".ti OR "telerehabilitation".ti OR "computer-assisted therapy"/ OR "computer-assisted therapy".ti OR "computer assisted therapy".ti OR "online therapy".ti OR "Computer Mediated Communication".ti OR "Computer Mediated Communications".ti OR **"remote".ti OR "on-line".ti OR "on line".ti** OR "wearable devices".ti OR "wearable device".ti OR "healthsensor".ti OR "healthsensors".ti OR "health sensor".ti OR "health sensors".ti OR exp *"Robotics"/ OR "robotics".ti OR robot*.ti OR "exergame".ti OR "exergames".ti OR exergam*.ti OR exp *"Video Game"/ OR "app-based".ti OR "wearable app".ti OR "wearable apps".ti OR "wearables".ti OR "Nintendo".ti OR "Wii".ti OR "gaming console".ti OR "gaming consoles".ti)) NOT (conference review or conference abstract).pt

PsycINFO
http://search.ebscohost.com/login.aspx?authtype=ip,uid&profile=lumc&defaultdb=psyh

8-1-2021

1.

(TI("Rehabilitation" OR rehab* OR "Rehabilitation Nursing" OR "Rehabilitation Center" OR "Activities of Daily Living" OR "Animal Assisted Therapy" OR "Equine-Assisted Therapy" OR "Art Therapy" OR "Bibliotherapy" OR "Correction of Hearing Impairment" OR "Total Communication Methods" OR "Lipreading" OR "Manual Communication" OR "Dance Therapy" OR "Early Ambulation" OR "Exercise Therapy" OR "Endurance Training" OR "Continuous Passive Motion Therapy" OR "Muscle Stretching" OR "Plyometric Exercise" OR "Resistance Training" OR "Music Therapy" OR "Occupational Therapy" OR "Recreation Therapy" OR "Language Therapy" OR "Myofunctional Therapy" OR "Speech Therapy" OR "Alaryngeal Speech" OR "Voice Training" OR "Telerehabilitation" OR "Activity of Daily Living" OR "Plyometric Exercises" OR "Exercise" OR "exercise" OR "exercises" OR "physical activity") AND TI("elderly" OR "elder" OR "elders" OR geriatr* OR "Homes for the Aged" OR "Elderly Care" OR "Senior Centers" OR older person* OR old person* OR older patient* OR old patient* OR "older women" OR "old women" OR "older men" OR "old men" OR old adult* OR older adult* OR "Older individual" OR "Older individuals" OR "old people" OR "older people" OR "Oldest Old" OR "Nonagenarians" OR "Nonagenarian" OR "Octogenarians" OR "Octogenarian" OR "Centenarians" OR "Centenarian" OR "septuagenarian" OR "septuagenarians" OR "Aging" OR "aging" OR "ageing" OR "older population" OR "aging population" OR "aging population" OR geront* OR "old-aged" OR "old-age" OR "old aged" OR "old age") AND TI("accelerometers" OR "accelerometer" OR "accelerometry" OR acceleromet* OR "Telehealth" OR "web portals" OR "web portal" OR "web portal" OR "e-consult" OR econsult* OR telemed* OR "ehealth" OR "e-health" OR "mhealth" OR "m-health" OR "mobile health" OR "telehealth" OR "tele-health" OR "tele health" OR "**electronic communication**" OR "remote consultation" OR "remote health care" OR "remote healthcare" OR "remote care" OR "remote monitoring" OR "teleconsultation" OR teleconsult* OR **mobile* OR "webbased" OR "web-based" OR "Electronic Mail" OR "Electronic Mail" OR "e-mail" OR email*** OR "Mobile Application" OR "mobile apps" OR "mobile app" OR webapp* OR "SMS" OR "Cell Phones" OR "Smartphone" OR "Text Messaging" OR "Mobile Phone" OR "Cell Phone" OR "Smartphones" OR iphon* OR "text messag" OR "texting" OR **"mobile"** OR "cellular phone" OR "cellular phones" OR "smart phone" OR "telemedicine" OR "tele-care" OR "telecare" OR "tele-monitoring" OR "telemonitoring" OR **"website" OR "websites"** OR "personal digital assistant" OR "computer-assisted instruction" OR "ipad" OR ipad* OR "telenursing" OR telenurs* OR "virtual community" **OR "webpage" OR "webpages"** OR "web application" OR "web applications" OR "short message service" OR **"Internet" OR "internet" OR "online" OR "digital" OR digital*** OR "Reminder System" OR "Reminder Systems" OR

"Reminder System" OR "Reminder Device" OR "Reminder Devices" OR "reminder messages" OR "reminder message" OR **"web"** OR "Virtual Reality" OR "Virtual Reality" OR "smart technology" OR "smart technol" OR "wearable technology" OR "wearable technologies" OR "telerehabilitation" OR "computer-assisted therapy" OR "computer-assisted therapy" OR "computer assisted therapy" OR "online therapy" OR "Computer Mediated Communication" OR "Computer Mediated Communications" OR **"remote" OR "on-line" OR "on line"** OR "wearable devices" OR "wearable device" OR "healthsensor" OR "healthsensors" OR "health sensor" OR "health sensors" OR "Robotics" OR "robotics" OR robot* OR "exergame" OR "exergames" OR exergam* OR "Video Game" OR "app-based" OR "wearable app" OR "wearable apps" OR "wearables" OR "Nintendo" OR "Wii" OR "gaming console" OR "gaming consoles"))

(2) vier componenten in geval van usability

(TI("Rehabilitation" OR rehab* OR "Rehabilitation Nursing" OR "Rehabilitation Center" OR "Activities of Daily Living" OR "Animal Assisted Therapy" OR "Equine-Assisted Therapy" OR "Art Therapy" OR "Bibliotherapy" OR "Correction of Hearing Impairment" OR "Total Communication Methods" OR "Lipreading" OR "Manual Communication" OR "Dance Therapy" OR "Early Ambulation" OR "Exercise Therapy" OR "Endurance Training" OR "Continuous Passive Motion Therapy" OR "Muscle Stretching" OR "Plyometric Exercise" OR "Resistance Training" OR "Music Therapy" OR "Occupational Therapy" OR "Recreation Therapy" OR "Language Therapy" OR "Myofunctional Therapy" OR "Speech Therapy" OR "Alaryngeal Speech" OR "Voice Training" OR "Telerehabilitation" OR "Activity of Daily Living" OR "Plyometric Exercises" OR "Exercise" OR "exercise" OR "exercises" OR "physical activity") AND TI("elderly" OR "elder" OR "elders" OR geriatr* OR "Homes for the Aged" OR "Elderly Care" OR "Senior Centers" OR older person* OR old person* OR older patient* OR old patient* OR "older women" OR "old women" OR "older men" OR "old men" OR old adult* OR older adult* OR "Older individual" OR "Older individuals" OR "old people" OR "older people" OR "Oldest Old" OR "Nonagenarians" OR "Nonagenarian" OR "Octogenarians" OR "Octogenarian" OR "Centenarians" OR "Centenarian" OR "septuagenarian" OR "septuagenarians" OR "Aging" OR "aging" OR "ageing" OR "older population" OR "aging population" OR "aging population" OR geront* OR "old-aged" OR "old-age" OR "old aged" OR "old age") AND TX("accelerometers" OR "accelerometer" OR "accelerometry" OR acceleromet* OR "Telehealth" OR "web portals" OR "web portal" OR "web portal" OR "e-consult" OR econsult* OR telemed* OR "ehealth" OR "e-health" OR "mhealth" OR "m-health" OR "mobile health" OR "telehealth" OR "tele-health" OR "tele health" OR "**electronic communication"** OR "remote consultation" OR "remote health care" OR "remote healthcare" OR "remote care" OR "remote monitoring" OR "teleconsultation" OR teleconsult* OR **mobile* OR "webbased" OR "web-based" OR "Electronic Mail" OR "Electronic Mail" OR "e-mail" OR email*** OR "Mobile Application" OR "mobile apps" OR "mobile app" OR webapp* OR "SMS" OR "Cell Phones" OR "Smartphone" OR "Text Messaging" OR "Mobile Phone" OR "Cell Phone" OR "Smartphones" OR iphon* OR "text messag" OR "texting" OR **"mobile"** OR "cellular phone" OR "cellular phones" OR "smart phone" OR "telemedicine" OR "tele-care" OR "telecare" OR "tele-monitoring" OR

"telemonitoring" OR **"website" OR "websites"** OR "personal digital assistant" OR "computer-assisted instruction" OR "ipad" OR ipad* OR "telenursing" OR telenurs* OR "virtual community" **OR "webpage" OR "webpages"** OR "web application" OR "web applications" OR "short message service" OR **"Internet" OR "internet" OR "online" OR "digital" OR digital*** OR "Reminder System" OR "Reminder Systems" OR "Reminder System" OR "Reminder Device" OR "Reminder Devices" OR "reminder messages" OR "reminder message" OR **"web"** OR "Virtual Reality" OR "Virtual Reality" OR "smart technology" OR "smart technol" OR "wearable technology" OR "wearable technologies" OR "telerehabilitation" OR "computer-assisted therapy" OR "computer-assisted therapy" OR "computer assisted therapy" OR "online therapy" OR "Computer Mediated Communication" OR "Computer Mediated Communications" OR **"remote" OR "on-line" OR "on line"** OR "wearable devices" OR "wearable device" OR "healthsensor" OR "healthsensors" OR "health sensor" OR "health sensors" OR "Robotics" OR "robotics" OR robot* OR "exergame" OR "exergames" OR exergam* OR "Video Game" OR "app-based" OR "wearable app" OR "wearable apps" OR "wearables" OR "Nintendo" OR "Wii" OR "gaming console" OR "gaming consoles"))
OR (TX("Rehabilitation" OR rehab* OR "Rehabilitation Nursing" OR "Rehabilitation Center" OR "Activities of Daily Living" OR "Animal Assisted Therapy" OR "Equine-Assisted Therapy" OR "Art Therapy" OR "Bibliotherapy" OR "Correction of Hearing Impairment" OR "Total Communication Methods" OR "Lipreading" OR "Manual Communication" OR "Dance Therapy" OR "Early Ambulation" OR "Exercise Therapy" OR "Endurance Training" OR "Continuous Passive Motion Therapy" OR "Muscle Stretching" OR "Plyometric Exercise" OR "Resistance Training" OR "Music Therapy" OR "Occupational Therapy" OR "Recreation Therapy" OR "Language Therapy" OR "Myofunctional Therapy" OR "Speech Therapy" OR "Alaryngeal Speech" OR "Voice Training" OR "Telerehabilitation" OR "Activity of Daily Living" OR "Plyometric Exercises" OR "Exercise" OR "exercise" OR "exercises" OR "physical activity") AND TI("elderly" OR "elder" OR "elders" OR geriatr* OR "Homes for the Aged" OR "Elderly Care" OR "Senior Centers" OR older person* OR old person* OR older patient* OR old patient* OR "older women" OR "old women" OR "older men" OR "old men" OR old adult* OR older adult* OR "Older individual" OR "Older individuals" OR "old people" OR "older people" OR "Oldest Old" OR "Nonagenarians" OR "Nonagenarian" OR "Octogenarians" OR "Octogenarian" OR "Centenarians" OR "Centenarian" OR "septuagenarian" OR "septuagenarians" OR "Aging" OR "aging" OR "ageing" OR "older population" OR "aging population" OR "aging population" OR geront* OR "old-aged" OR "old-age" OR "old aged" OR "old age") AND TI("accelerometers" OR "accelerometer" OR "accelerometry" OR acceleromet* OR "Telehealth" OR "web portals" OR "web portal" OR "web portal" OR "e-consult" OR econsult* OR telemed* OR "ehealth" OR "e-health" OR "mhealth" OR "m-health" OR "mobile health" OR "telehealth" OR "tele-health" OR "tele health" OR "**electronic communication"** OR "remote consultation" OR "remote health care" OR "remote healthcare" OR "remote care" OR "remote monitoring" OR "teleconsultation" OR teleconsult* OR **mobile* OR "webbased" OR "web-based" OR "Electronic Mail" OR "Electronic Mail" OR "e-mail" OR email*** OR "Mobile Application" OR "mobile apps" OR "mobile app" OR webapp* OR "SMS" OR "Cell Phones" OR "Smartphone" OR "Text Messaging" OR "Mobile Phone" OR "Cell Phone" OR "Smartphones" OR iphon* OR "text messag" OR

"texting" OR **"mobile"** OR "cellular phone" OR "cellular phones" OR "smart phone" OR "telemedicine" OR "tele-care" OR "telecare" OR "tele-monitoring" OR "telemonitoring" OR **"website" OR "websites"** OR "personal digital assistant" OR "computer-assisted instruction" OR "ipad" OR ipad* OR "telenursing" OR telenurs* OR "virtual community" **OR "webpage" OR "webpages"** OR "web application" OR "web applications" OR "short message service" OR **"Internet" OR "internet" OR "online" OR "digital" OR digital*** OR "Reminder System" OR "Reminder Systems" OR "Reminder System" OR "Reminder Device" OR "Reminder Devices" OR "reminder messages" OR "reminder message" OR **"web"** OR "Virtual Reality" OR "Virtual Reality" OR "smart technology" OR "smart technol" OR "wearable technology" OR "wearable technologies" OR "telerehabilitation" OR "computer-assisted therapy" OR "computer-assisted therapy" OR "computer assisted therapy" OR "online therapy" OR "Computer Mediated Communication" OR "Computer Mediated Communications" OR **"remote" OR "on-line" OR "on line"** OR "wearable devices" OR "wearable device" OR "healthsensor" OR "healthsensors" OR "health sensor" OR "health sensors" OR "Robotics" OR "robotics" OR robot* OR "exergame" OR "exergames" OR exergam* OR "Video Game" OR "app-based" OR "wearable app" OR "wearable apps" OR "wearables" OR "Nintendo" OR "Wii" OR "gaming console" OR "gaming consoles"))

OR (TI("Rehabilitation" OR rehab* OR "Rehabilitation Nursing" OR "Rehabilitation Center" OR "Activities of Daily Living" OR "Animal Assisted Therapy" OR "Equine-Assisted Therapy" OR "Art Therapy" OR "Bibliotherapy" OR "Correction of Hearing Impairment" OR "Total Communication Methods" OR "Lipreading" OR "Manual Communication" OR "Dance Therapy" OR "Early Ambulation" OR "Exercise Therapy" OR "Endurance Training" OR "Continuous Passive Motion Therapy" OR "Muscle Stretching" OR "Plyometric Exercise" OR "Resistance Training" OR "Music Therapy" OR "Occupational Therapy" OR "Recreation Therapy" OR "Language Therapy" OR "Myofunctional Therapy" OR "Speech Therapy" OR "Alaryngeal Speech" OR "Voice Training" OR "Telerehabilitation" OR "Activity of Daily Living" OR "Plyometric Exercises" OR "Exercise" OR "exercise" OR "exercises" OR "physical activity") AND SU("elderly" OR "elder" OR "elders" OR geriatr* OR "Homes for the Aged" OR "Elderly Care" OR "Senior Centers" OR older person* OR old person* OR older patient* OR old patient* OR "older women" OR "old women" OR "older men" OR "old men" OR old adult* OR older adult* OR "Older individual" OR "Older individuals" OR "old people" OR "older people" OR "Oldest Old" OR "Nonagenarians" OR "Nonagenarian" OR "Octogenarians" OR "Octogenarian" OR "Centenarians" OR "Centenarian" OR "septuagenarian" OR "septuagenarians" OR "Aging" OR "aging" OR "ageing" OR "older population" OR "aging population" OR "aging population" OR geront* OR "old-aged" OR "old-age" OR "old aged" OR "old age") AND TX("accelerometers" OR "accelerometer" OR "accelerometry" OR acceleromet* OR "Telehealth" OR "web portals" OR "web portal" OR "web portal" OR "e-consult" OR econsult* OR telemed* OR "ehealth" OR "e-health" OR "mhealth" OR "m-health" OR "mobile health" OR "telehealth" OR "tele-health" OR "tele health" OR "**electronic communication"** OR "remote consultation" OR "remote health care" OR "remote healthcare" OR "remote care" OR "remote monitoring" OR "teleconsultation" OR teleconsult* OR **mobile* OR "webbased" OR "web-based" OR "Electronic Mail" OR "Electronic Mail" OR "e-mail" OR email*** OR "Mobile Application" OR "mobile apps" OR "mobile app" OR

webapp* OR "SMS" OR "Cell Phones" OR "Smartphone" OR "Text Messaging" OR "Mobile Phone" OR "Cell Phone" OR "Smartphones" OR iphon* OR "text messag" OR "texting" OR **"mobile"** OR "cellular phone" OR "cellular phones" OR "smart phone" OR "telemedicine" OR "tele-care" OR "telecare" OR "tele-monitoring" OR "telemonitoring" OR **"website" OR "websites"** OR "personal digital assistant" OR "computer-assisted instruction" OR "ipad" OR ipad* OR "telenursing" OR telenurs* OR "virtual community" **OR "webpage" OR "webpages"** OR "web application" OR "web applications" OR "short message service" OR **"Internet" OR "internet" OR "online" OR "digital" OR digital*** OR "Reminder System" OR "Reminder Systems" OR "Reminder System" OR "Reminder Device" OR "Reminder Devices" OR "reminder messages" OR "reminder message" OR **"web"** OR "Virtual Reality" OR "Virtual Reality" OR "smart technology" OR "smart technol" OR "wearable technology" OR "wearable technologies" OR "telerehabilitation" OR "computer-assisted therapy" OR "computer-assisted therapy" OR "computer assisted therapy" OR "online therapy" OR "Computer Mediated Communication" OR "Computer Mediated Communications" OR **"remote" OR "on-line" OR "on line"** OR "wearable devices" OR "wearable device" OR "healthsensor" OR "healthsensors" OR "health sensor" OR "health sensors" OR "Robotics" OR "robotics" OR robot* OR "exergame" OR "exergames" OR exergam* OR "Video Game" OR "app-based" OR "wearable app" OR "wearable apps" OR "wearables" OR "Nintendo" OR "Wii" OR "gaming console" OR "gaming consoles"))
OR (TX("Rehabilitation" OR rehab* OR "Rehabilitation Nursing" OR "Rehabilitation Center" OR "Activities of Daily Living" OR "Animal Assisted Therapy" OR "Equine-Assisted Therapy" OR "Art Therapy" OR "Bibliotherapy" OR "Correction of Hearing Impairment" OR "Total Communication Methods" OR "Lipreading" OR "Manual Communication" OR "Dance Therapy" OR "Early Ambulation" OR "Exercise Therapy" OR "Endurance Training" OR "Continuous Passive Motion Therapy" OR "Muscle Stretching" OR "Plyometric Exercise" OR "Resistance Training" OR "Music Therapy" OR "Occupational Therapy" OR "Recreation Therapy" OR "Language Therapy" OR "Myofunctional Therapy" OR "Speech Therapy" OR "Alaryngeal Speech" OR "Voice Training" OR "Telerehabilitation" OR "Activity of Daily Living" OR "Plyometric Exercises" OR "Exercise" OR "exercise" OR "exercises" OR "physical activity") AND SU("elderly" OR "elder" OR "elders" OR geriatr* OR "Homes for the Aged" OR "Elderly Care" OR "Senior Centers" OR older person* OR old person* OR older patient* OR old patient* OR "older women" OR "old women" OR "older men" OR "old men" OR old adult* OR older adult* OR "Older individual" OR "Older individuals" OR "old people" OR "older people" OR "Oldest Old" OR "Nonagenarians" OR "Nonagenarian" OR "Octogenarians" OR "Octogenarian" OR "Centenarians" OR "Centenarian" OR "septuagenarian" OR "septuagenarians" OR "Aging" OR "aging" OR "ageing" OR "older population" OR "aging population" OR "aging population" OR geront* OR "old-aged" OR "old-age" OR "old aged" OR "old age") AND TI("accelerometers" OR "accelerometer" OR "accelerometry" OR acceleromet* OR "Telehealth" OR "web portals" OR "web portal" OR "web portal" OR "e-consult" OR econsult* OR telemed* OR "ehealth" OR "e-health" OR "mhealth" OR "m-health" OR "mobile health" OR "telehealth" OR "tele-health" OR "tele health" OR "**electronic communication"** OR "remote consultation" OR "remote health care" OR "remote healthcare" OR "remote care" OR "remote monitoring" OR "teleconsultation" OR teleconsult* OR **mobile* OR**

"webbased" OR "web-based" OR "Electronic Mail" OR "Electronic Mail" OR "e-mail" OR email* OR "Mobile Application" OR "mobile apps" OR "mobile app" OR webapp* OR "SMS" OR "Cell Phones" OR "Smartphone" OR "Text Messaging" OR "Mobile Phone" OR "Cell Phone" OR "Smartphones" OR iphon* OR "text messag" OR "texting" OR **"mobile"** OR "cellular phone" OR "cellular phones" OR "smart phone" OR "telemedicine" OR "tele-care" OR "telecare" OR "tele-monitoring" OR "telemonitoring" OR **"website" OR "websites"** OR "personal digital assistant" OR "computer-assisted instruction" OR "ipad" OR ipad* OR "telenursing" OR telenurs* OR "virtual community" **OR "webpage" OR "webpages"** OR "web application" OR "web applications" OR "short message service" OR **"Internet" OR "internet" OR "online" OR "digital" OR digital*** OR "Reminder System" OR "Reminder Systems" OR "Reminder System" OR "Reminder Device" OR "Reminder Devices" OR "reminder messages" OR "reminder message" OR **"web"** OR "Virtual Reality" OR "Virtual Reality" OR "smart technology" OR "smart technol" OR "wearable technology" OR "wearable technologies" OR "telerehabilitation" OR "computer-assisted therapy" OR "computer-assisted therapy" OR "computer assisted therapy" OR "online therapy" OR "Computer Mediated Communication" OR "Computer Mediated Communications" OR **"remote" OR "on-line" OR "on line"** OR "wearable devices" OR "wearable device" OR "healthsensor" OR "healthsensors" OR "health sensor" OR "health sensors" OR "Robotics" OR "robotics" OR robot* OR "exergame" OR "exergames" OR exergam* OR "Video Game" OR "app-based" OR "wearable app" OR "wearable apps" OR "wearables" OR "Nintendo" OR "Wii" OR "gaming console" OR "gaming consoles"))

AND (TX("usability" OR "Meaningful Use" OR "User experience" OR "User experiences" OR "usefulness") OR DE "Human Factors Engineering")

(3) Een nieuwe zoekstring met een omschrijving van de term feasibility. Ook weer met de nadruk op alle vier de componenten

TX("feasibility" OR "feasible") AND (TI("Rehabilitation" OR rehab* OR "Rehabilitation Nursing" OR "Rehabilitation Center" OR "Activities of Daily Living" OR "Animal Assisted Therapy" OR "Equine-Assisted Therapy" OR "Art Therapy" OR "Bibliotherapy" OR "Correction of Hearing Impairment" OR "Total Communication Methods" OR "Lipreading" OR "Manual Communication" OR "Dance Therapy" OR "Early Ambulation" OR "Exercise Therapy" OR "Endurance Training" OR "Continuous Passive Motion Therapy" OR "Muscle Stretching" OR "Plyometric Exercise" OR "Resistance Training" OR "Music Therapy" OR "Occupational Therapy" OR "Recreation Therapy" OR "Language Therapy" OR "Myofunctional Therapy" OR "Speech Therapy" OR "Alaryngeal Speech" OR "Voice Training" OR "Telerehabilitation" OR "Activity of Daily Living" OR "Plyometric Exercises" OR "Exercise" OR "exercise" OR "exercises" OR "physical activity") AND TI("elderly" OR "elder" OR "elders" OR geriatr* OR "Homes for the Aged" OR "Elderly Care" OR "Senior Centers" OR older person* OR old person* OR older patient* OR old patient* OR "older women" OR "old women" OR "older men" OR "old men" OR old adult* OR older adult* OR "Older individual" OR "Older individuals" OR "old people" OR "older people" OR "Oldest Old" OR "Nonagenarians" OR "Nonagenarian" OR "Octogenarians" OR "Octogenarian" OR

"Centenarians" OR "Centenarian" OR "septuagenarian" OR "septuagenarians" OR "Aging" OR "aging" OR "ageing" OR "older population" OR "aging population" OR "aging population" OR geront* OR "old-aged" OR "old-age" OR "old aged" OR "old age") AND TI("accelerometers" OR "accelerometer" OR "accelerometry" OR acceleromet* OR "Telehealth" OR "web portals" OR "web portal" OR "web portal" OR "e-consult" OR econsult* OR telemed* OR "ehealth" OR "e-health" OR "mhealth" OR "m-health" OR "mobile health" OR "telehealth" OR "tele-health" OR "tele health" OR "**electronic communication"** OR "remote consultation" OR "remote health care" OR "remote healthcare" OR "remote care" OR "remote monitoring" OR "teleconsultation" OR teleconsult* OR **mobile* OR "webbased" OR "web-based" OR "Electronic Mail" OR "Electronic Mail" OR "e-mail" OR email*** OR "Mobile Application" OR "mobile apps" OR "mobile app" OR webapp* OR "SMS" OR "Cell Phones" OR "Smartphone" OR "Text Messaging" OR "Mobile Phone" OR "Cell Phone" OR "Smartphones" OR iphon* OR "text messag" OR "texting" OR **"mobile"** OR "cellular phone" OR "cellular phones" OR "smart phone" OR "telemedicine" OR "tele-care" OR "telecare" OR "tele-monitoring" OR "telemonitoring" OR **"website" OR "websites"** OR "personal digital assistant" OR "computer-assisted instruction" OR "ipad" OR ipad* OR "telenursing" OR telenurs* OR "virtual community" **OR "webpage" OR "webpages"** OR "web application" OR "web applications" OR "short message service" OR **"Internet" OR "internet" OR "online" OR "digital" OR digital*** OR "Reminder System" OR "Reminder Systems" OR "Reminder System" OR "Reminder Device" OR "Reminder Devices" OR "reminder messages" OR "reminder message" OR **"web"** OR "Virtual Reality" OR "Virtual Reality" OR "smart technology" OR "smart technol" OR "wearable technology" OR "wearable technologies" OR "telerehabilitation" OR "computer-assisted therapy" OR "computer-assisted therapy" OR "computer assisted therapy" OR "online therapy" OR "Computer Mediated Communication" OR "Computer Mediated Communications" OR **"remote" OR "on-line" OR "on line"** OR "wearable devices" OR "wearable device" OR "healthsensor" OR "healthsensors" OR "health sensor" OR "health sensors" OR "Robotics" OR "robotics" OR robot* OR "exergame" OR "exergames" OR exergam* OR "Video Game" OR "app-based" OR "wearable app" OR "wearable apps" OR "wearables" OR "Nintendo" OR "Wii" OR "gaming console" OR "gaming consoles"))

10-4-2019

B. Nadruk op Revalidatie en/óf E-health

(TI("Rehabilitation" OR rehab* OR "Rehabilitation Nursing" OR "Rehabilitation Center" OR "Activities of Daily Living" OR "Animal Assisted Therapy" OR "Equine-Assisted Therapy" OR "Art Therapy" OR "Bibliotherapy" OR "Correction of Hearing Impairment" OR "Total Communication Methods" OR "Lipreading" OR "Manual Communication" OR "Dance Therapy" OR "Early Ambulation" OR "Exercise Therapy" OR "Endurance Training" OR "Continuous Passive Motion Therapy" OR "Muscle Stretching" OR "Plyometric Exercise" OR "Resistance Training" OR "Music Therapy"

OR "Occupational Therapy" OR "Recreation Therapy" OR "Language Therapy" OR "Myofunctional Therapy" OR "Speech Therapy" OR "Alaryngeal Speech" OR "Voice Training" OR "Telerehabilitation" OR "Activity of Daily Living" OR "Plyometric Exercises" OR "Exercise" OR "exercise" OR "exercises" OR "physical activity") AND TI("elderly" OR "elder" OR "elders" OR geriatr* OR "Homes for the Aged" OR "Elderly Care" OR "Senior Centers" OR older person* OR old person* OR older patient* OR old patient* OR "older women" OR "old women" OR "older men" OR "old men" OR old adult* OR older adult* OR "Older individual" OR "Older individuals" OR "old people" OR "older people" OR "Oldest Old" OR "Nonagenarians" OR "Nonagenarian" OR "Octogenarians" OR "Octogenarian" OR "Centenarians" OR "Centenarian" OR "septuagenarian" OR "septuagenarians" OR "Aging" OR "aging" OR "ageing" OR "older population" OR "aging population" OR "aging population" OR geront* OR "old-aged" OR "old-age" OR "old aged" OR "old age") AND TX("accelerometers" OR "accelerometer" OR "accelerometry" OR acceleromet* OR "Telehealth" OR "web portals" OR "web portal" OR "web portal" OR "e-consult" OR econsult* OR telemed* OR "ehealth" OR "e-health" OR "mhealth" OR "m-health" OR "mobile health" OR "telehealth" OR "tele-health" OR "tele health" OR "**electronic communication**" OR "remote consultation" OR "remote health care" OR "remote healthcare" OR "remote care" OR "remote monitoring" OR "teleconsultation" OR teleconsult* OR **mobile* OR "webbased" OR "web-based" OR "Electronic Mail" OR "Electronic Mail" OR "e-mail" OR email*** OR "Mobile Application" OR "mobile apps" OR "mobile app" OR webapp* OR "SMS" OR "Cell Phones" OR "Smartphone" OR "Text Messaging" OR "Mobile Phone" OR "Cell Phone" OR "Smartphones" OR iphon* OR "text messag" OR "texting" OR **"mobile"** OR "cellular phone" OR "cellular phones" OR "smart phone" OR "telemedicine" OR "tele-care" OR "telecare" OR "tele-monitoring" OR "telemonitoring" OR **"website" OR "websites"** OR "personal digital assistant" OR "computer-assisted instruction" OR "ipad" OR ipad* OR "telenursing" OR telenurs* OR "virtual community" **OR "webpage" OR "webpages"** OR "web application" OR "web applications" OR "short message service" OR **"Internet" OR "internet" OR "online" OR "digital" OR digital*** OR "Reminder System" OR "Reminder Systems" OR "Reminder System" OR "Reminder Device" OR "Reminder Devices" OR "reminder messages" OR "reminder message" OR **"web"** OR "Virtual Reality" OR "Virtual Reality" OR "smart technology" OR "smart technol" OR "wearable technology" OR "wearable technologies" OR "telerehabilitation" OR "computer-assisted therapy" OR "computer-assisted therapy" OR "computer assisted therapy" OR "online therapy" OR "Computer Mediated Communication" OR "Computer Mediated Communications" OR **"remote" OR "on-line" OR "on line"** OR "wearable devices" OR "wearable device" OR "healthsensor" OR "healthsensors" OR "health sensor" OR "health sensors" OR "Robotics" OR "robotics" OR robot* OR "exergame" OR "exergames" OR exergam* OR "Video Game" OR "app-based" OR "wearable app" OR "wearable apps" OR "wearables" OR "Nintendo" OR "Wii" OR "gaming console" OR "gaming consoles"))
OR (TX("Rehabilitation" OR rehab* OR "Rehabilitation Nursing" OR "Rehabilitation Center" OR "Activities of Daily Living" OR "Animal Assisted Therapy" OR "Equine-Assisted Therapy" OR "Art Therapy" OR "Bibliotherapy" OR "Correction of Hearing Impairment" OR "Total Communication Methods" OR "Lipreading" OR "Manual Communication" OR "Dance Therapy" OR "Early Ambulation" OR "Exercise Therapy"

OR "Endurance Training" OR "Continuous Passive Motion Therapy" OR "Muscle Stretching" OR "Plyometric Exercise" OR "Resistance Training" OR "Music Therapy" OR "Occupational Therapy" OR "Recreation Therapy" OR "Language Therapy" OR "Myofunctional Therapy" OR "Speech Therapy" OR "Alaryngeal Speech" OR "Voice Training" OR "Telerehabilitation" OR "Activity of Daily Living" OR "Plyometric Exercises" OR "Exercise" OR "exercise" OR "exercises" OR "physical activity") AND TI("elderly" OR "elder" OR "elders" OR geriatr* OR "Homes for the Aged" OR "Elderly Care" OR "Senior Centers" OR older person* OR old person* OR older patient* OR old patient* OR "older women" OR "old women" OR "older men" OR "old men" OR old adult* OR older adult* OR "Older individual" OR "Older individuals" OR "old people" OR "older people" OR "Oldest Old" OR "Nonagenarians" OR "Nonagenarian" OR "Octogenarians" OR "Octogenarian" OR "Centenarians" OR "Centenarian" OR "septuagenarian" OR "septuagenarians" OR "Aging" OR "aging" OR "ageing" OR "older population" OR "aging population" OR "aging population" OR geront* OR "old-aged" OR "old-age" OR "old aged" OR "old age") AND TI("accelerometers" OR "accelerometer" OR "accelerometry" OR acceleromet* OR "Telehealth" OR "web portals" OR "web portal" OR "web portal" OR "e-consult" OR econsult* OR telemed* OR "ehealth" OR "e-health" OR "mhealth" OR "m-health" OR "mobile health" OR "telehealth" OR "tele-health" OR "tele health" OR "**electronic communication**" OR "remote consultation" OR "remote health care" OR "remote healthcare" OR "remote care" OR "remote monitoring" OR "teleconsultation" OR teleconsult* OR **mobile* OR "webbased" OR "web-based" OR "Electronic Mail" OR "Electronic Mail" OR "e-mail" OR email*** OR "Mobile Application" OR "mobile apps" OR "mobile app" OR webapp* OR "SMS" OR "Cell Phones" OR "Smartphone" OR "Text Messaging" OR "Mobile Phone" OR "Cell Phone" OR "Smartphones" OR iphon* OR "text messag" OR "texting" OR **"mobile"** OR "cellular phone" OR "cellular phones" OR "smart phone" OR "telemedicine" OR "tele-care" OR "telecare" OR "tele-monitoring" OR "telemonitoring" OR **"website" OR "websites"** OR "personal digital assistant" OR "computer-assisted instruction" OR "ipad" OR ipad* OR "telenursing" OR telenurs* OR "virtual community" **OR "webpage" OR "webpages"** OR "web application" OR "web applications" OR "short message service" OR **"Internet" OR "internet" OR "online" OR "digital" OR digital*** OR "Reminder System" OR "Reminder Systems" OR "Reminder System" OR "Reminder Device" OR "Reminder Devices" OR "reminder messages" OR "reminder message" OR **"web"** OR "Virtual Reality" OR "Virtual Reality" OR "smart technology" OR "smart technol" OR "wearable technology" OR "wearable technologies" OR "telerehabilitation" OR "computer-assisted therapy" OR "computer-assisted therapy" OR "computer assisted therapy" OR "online therapy" OR "Computer Mediated Communication" OR "Computer Mediated Communications" OR **"remote" OR "on-line" OR "on line"** OR "wearable devices" OR "wearable device" OR "healthsensor" OR "healthsensors" OR "health sensor" OR "health sensors" OR "Robotics" OR "robotics" OR robot* OR "exergame" OR "exergames" OR exergam* OR "Video Game" OR "app-based" OR "wearable app" OR "wearable apps" OR "wearables" OR "Nintendo" OR "Wii" OR "gaming console" OR "gaming consoles"))

OR (TI("Rehabilitation" OR rehab* OR "Rehabilitation Nursing" OR "Rehabilitation Center" OR "Activities of Daily Living" OR "Animal Assisted Therapy" OR "Equine-Assisted Therapy" OR "Art Therapy" OR "Bibliotherapy" OR "Correction of Hearing

Impairment" OR "Total Communication Methods" OR "Lipreading" OR "Manual Communication" OR "Dance Therapy" OR "Early Ambulation" OR "Exercise Therapy" OR "Endurance Training" OR "Continuous Passive Motion Therapy" OR "Muscle Stretching" OR "Plyometric Exercise" OR "Resistance Training" OR "Music Therapy" OR "Occupational Therapy" OR "Recreation Therapy" OR "Language Therapy" OR "Myofunctional Therapy" OR "Speech Therapy" OR "Alaryngeal Speech" OR "Voice Training" OR "Telerehabilitation" OR "Activity of Daily Living" OR "Plyometric Exercises" OR "Exercise" OR "exercise" OR "exercises" OR "physical activity") AND SU("elderly" OR "elder" OR "elders" OR geriatr* OR "Homes for the Aged" OR "Elderly Care" OR "Senior Centers" OR older person* OR old person* OR older patient* OR old patient* OR "older women" OR "old women" OR "older men" OR "old men" OR old adult* OR older adult* OR "Older individual" OR "Older individuals" OR "old people" OR "older people" OR "Oldest Old" OR "Nonagenarians" OR "Nonagenarian" OR "Octogenarians" OR "Octogenarian" OR "Centenarians" OR "Centenarian" OR "septuagenarian" OR "septuagenarians" OR "Aging" OR "aging" OR "ageing" OR "older population" OR "aging population" OR "aging population" OR geront* OR "old-aged" OR "old-age" OR "old aged" OR "old age") AND TX("accelerometers" OR "accelerometer" OR "accelerometry" OR acceleromet* OR "Telehealth" OR "web portals" OR "web portal" OR "web portal" OR "e-consult" OR econsult* OR telemed* OR "ehealth" OR "e-health" OR "mhealth" OR "m-health" OR "mobile health" OR "telehealth" OR "tele-health" OR "tele health" OR "**electronic communication**" OR "remote consultation" OR "remote health care" OR "remote healthcare" OR "remote care" OR "remote monitoring" OR "teleconsultation" OR teleconsult* OR **mobile* OR "webbased" OR "web-based" OR "Electronic Mail" OR "Electronic Mail" OR "e-mail" OR email*** OR "Mobile Application" OR "mobile apps" OR "mobile app" OR webapp* OR "SMS" OR "Cell Phones" OR "Smartphone" OR "Text Messaging" OR "Mobile Phone" OR "Cell Phone" OR "Smartphones" OR iphon* OR "text messag" OR "texting" OR **"mobile"** OR "cellular phone" OR "cellular phones" OR "smart phone" OR "telemedicine" OR "tele-care" OR "telecare" OR "tele-monitoring" OR "telemonitoring" OR **"website" OR "websites"** OR "personal digital assistant" OR "computer-assisted instruction" OR "ipad" OR ipad* OR "telenursing" OR telenurs* OR "virtual community" **OR "webpage" OR "webpages"** OR "web application" OR "web applications" OR "short message service" OR **"Internet" OR "internet" OR "online" OR "digital" OR digital*** OR "Reminder System" OR "Reminder Systems" OR "Reminder System" OR "Reminder Device" OR "Reminder Devices" OR "reminder messages" OR "reminder message" OR **"web"** OR "Virtual Reality" OR "Virtual Reality" OR "smart technology" OR "smart technol" OR "wearable technology" OR "wearable technologies" OR "telerehabilitation" OR "computer-assisted therapy" OR "computer-assisted therapy" OR "computer assisted therapy" OR "online therapy" OR "Computer Mediated Communication" OR "Computer Mediated Communications" OR **"remote" OR "on-line" OR "on line"** OR "wearable devices" OR "wearable device" OR "healthsensor" OR "healthsensors" OR "health sensor" OR "health sensors" OR "Robotics" OR "robotics" OR robot* OR "exergame" OR "exergames" OR exergam* OR "Video Game" OR "app-based" OR "wearable app" OR "wearable apps" OR "wearables" OR "Nintendo" OR "Wii" OR "gaming console" OR "gaming consoles"))

OR (TX("Rehabilitation" OR rehab* OR "Rehabilitation Nursing" OR "Rehabilitation

Center" OR "Activities of Daily Living" OR "Animal Assisted Therapy" OR "Equine-Assisted Therapy" OR "Art Therapy" OR "Bibliotherapy" OR "Correction of Hearing Impairment" OR "Total Communication Methods" OR "Lipreading" OR "Manual Communication" OR "Dance Therapy" OR "Early Ambulation" OR "Exercise Therapy" OR "Endurance Training" OR "Continuous Passive Motion Therapy" OR "Muscle Stretching" OR "Plyometric Exercise" OR "Resistance Training" OR "Music Therapy" OR "Occupational Therapy" OR "Recreation Therapy" OR "Language Therapy" OR "Myofunctional Therapy" OR "Speech Therapy" OR "Alaryngeal Speech" OR "Voice Training" OR "Telerehabilitation" OR "Activity of Daily Living" OR "Plyometric Exercises" OR "Exercise" OR "exercise" OR "exercises" OR "physical activity") AND SU("elderly" OR "elder" OR "elders" OR geriatr* OR "Homes for the Aged" OR "Elderly Care" OR "Senior Centers" OR older person* OR old person* OR older patient* OR old patient* OR "older women" OR "old women" OR "older men" OR "old men" OR old adult* OR older adult* OR "Older individual" OR "Older individuals" OR "old people" OR "older people" OR "Oldest Old" OR "Nonagenarians" OR "Nonagenarian" OR "Octogenarians" OR "Octogenarian" OR "Centenarians" OR "Centenarian" OR "septuagenarian" OR "septuagenarians" OR "Aging" OR "aging" OR "ageing" OR "older population" OR "aging population" OR "aging population" OR geront* OR "old-aged" OR "old-age" OR "old aged" OR "old age") AND TI("accelerometers" OR "accelerometer" OR "accelerometry" OR acceleromet* OR "Telehealth" OR "web portals" OR "web portal" OR "web portal" OR "e-consult" OR econsult* OR telemed* OR "ehealth" OR "e-health" OR "mhealth" OR "m-health" OR "mobile health" OR "telehealth" OR "tele-health" OR "tele health" OR "**electronic communication**" OR "remote consultation" OR "remote health care" OR "remote healthcare" OR "remote care" OR "remote monitoring" OR "teleconsultation" OR teleconsult* OR **mobile* OR "webbased" OR "web-based" OR "Electronic Mail" OR "Electronic Mail" OR "e-mail" OR email*** OR "Mobile Application" OR "mobile apps" OR "mobile app" OR webapp* OR "SMS" OR "Cell Phones" OR "Smartphone" OR "Text Messaging" OR "Mobile Phone" OR "Cell Phone" OR "Smartphones" OR iphon* OR "text messag" OR "texting" OR **"mobile"** OR "cellular phone" OR "cellular phones" OR "smart phone" OR "telemedicine" OR "tele-care" OR "telecare" OR "tele-monitoring" OR "telemonitoring" OR **"website" OR "websites"** OR "personal digital assistant" OR "computer-assisted instruction" OR "ipad" OR ipad* OR "telenursing" OR telenurs* OR "virtual community" **OR "webpage" OR "webpages"** OR "web application" OR "web applications" OR "short message service" OR **"Internet" OR "internet" OR "online" OR "digital" OR digital*** OR "Reminder System" OR "Reminder Systems" OR "Reminder System" OR "Reminder Device" OR "Reminder Devices" OR "reminder messages" OR "reminder message" OR **"web"** OR "Virtual Reality" OR "Virtual Reality" OR "smart technology" OR "smart technol" OR "wearable technology" OR "wearable technologies" OR "telerehabilitation" OR "computer-assisted therapy" OR "computer-assisted therapy" OR "computer assisted therapy" OR "online therapy" OR "Computer Mediated Communication" OR "Computer Mediated Communications" OR **"remote" OR "on-line" OR "on line"** OR "wearable devices" OR "wearable device" OR "healthsensor" OR "healthsensors" OR "health sensor" OR "health sensors" OR "Robotics" OR "robotics" OR robot* OR "exergame" OR "exergames" OR exergam*

OR "Video Game" OR "app-based" OR "wearable app" OR "wearable apps" OR "wearables" OR "Nintendo" OR "Wii" OR "gaming console" OR "gaming consoles")))

B.2 - Nadruk op Revalidatie en/óf E-health – usability

(TI("Rehabilitation" OR rehab* OR "Rehabilitation Nursing" OR "Rehabilitation Center" OR "Activities of Daily Living" OR "Animal Assisted Therapy" OR "Equine-Assisted Therapy" OR "Art Therapy" OR "Bibliotherapy" OR "Correction of Hearing Impairment" OR "Total Communication Methods" OR "Lipreading" OR "Manual Communication" OR "Dance Therapy" OR "Early Ambulation" OR "Exercise Therapy" OR "Endurance Training" OR "Continuous Passive Motion Therapy" OR "Muscle Stretching" OR "Plyometric Exercise" OR "Resistance Training" OR "Music Therapy" OR "Occupational Therapy" OR "Recreation Therapy" OR "Language Therapy" OR "Myofunctional Therapy" OR "Speech Therapy" OR "Alaryngeal Speech" OR "Voice Training" OR "Telerehabilitation" OR "Activity of Daily Living" OR "Plyometric Exercises" OR "Exercise" OR "exercise" OR "exercises" OR "physical activity") AND TI("elderly" OR "elder" OR "elders" OR geriatr* OR "Homes for the Aged" OR "Elderly Care" OR "Senior Centers" OR older person* OR old person* OR older patient* OR old patient* OR "older women" OR "old women" OR "older men" OR "old men" OR old adult* OR older adult* OR "Older individual" OR "Older individuals" OR "old people" OR "older people" OR "Oldest Old" OR "Nonagenarians" OR "Nonagenarian" OR "Octogenarians" OR "Octogenarian" OR "Centenarians" OR "Centenarian" OR "septuagenarian" OR "septuagenarians" OR "Aging" OR "aging" OR "ageing" OR "older population" OR "aging population" OR "aging population" OR geront* OR "old-aged" OR "old-age" OR "old aged" OR "old age") AND TX("accelerometers" OR "accelerometer" OR "accelerometry" OR acceleromet* OR "Telehealth" OR "web portals" OR "web portal" OR "web portal" OR "e-consult" OR econsult* OR telemed* OR "ehealth" OR "e-health" OR "mhealth" OR "m-health" OR "mobile health" OR "telehealth" OR "tele-health" OR "tele health" OR "**electronic communication**" OR "remote consultation" OR "remote health care" OR "remote healthcare" OR "remote care" OR "remote monitoring" OR "teleconsultation" OR teleconsult* OR **mobile* OR "webbased" OR "web-based" OR "Electronic Mail" OR "Electronic Mail" OR "e-mail" OR email*** OR "Mobile Application" OR "mobile apps" OR "mobile app" OR webapp* OR "SMS" OR "Cell Phones" OR "Smartphone" OR "Text Messaging" OR "Mobile Phone" OR "Cell Phone" OR "Smartphones" OR iphon* OR "text messag" OR "texting" OR **"mobile"** OR "cellular phone" OR "cellular phones" OR "smart phone" OR "telemedicine" OR "tele-care" OR "telecare" OR "tele-monitoring" OR "telemonitoring" OR **"website" OR "websites"** OR "personal digital assistant" OR "computer-assisted instruction" OR "ipad" OR ipad* OR "telenursing" OR telenurs* OR "virtual community" **OR "webpage" OR "webpages"** OR "web application" OR "web applications" OR "short message service" OR **"Internet" OR "internet" OR "online" OR "digital" OR digital*** OR "Reminder System" OR "Reminder Systems" OR "Reminder System" OR "Reminder Device" OR "Reminder Devices" OR "reminder messages" OR "reminder message" OR **"web"** OR "Virtual Reality" OR "Virtual

Reality" OR "smart technology" OR "smart technol" OR "wearable technology" OR "wearable technologies" OR "telerehabilitation" OR "computer-assisted therapy" OR "computer-assisted therapy" OR "computer assisted therapy" OR "online therapy" OR "Computer Mediated Communication" OR "Computer Mediated Communications" OR **"remote" OR "on-line" OR "on line"** OR "wearable devices" OR "wearable device" OR "healthsensor" OR "healthsensors" OR "health sensor" OR "health sensors" OR "Robotics" OR "robotics" OR robot* OR "exergame" OR "exergames" OR exergam* OR "Video Game" OR "app-based" OR "wearable app" OR "wearable apps" OR "wearables" OR "Nintendo" OR "Wii" OR "gaming console" OR "gaming consoles"))
OR (TX("Rehabilitation" OR rehab* OR "Rehabilitation Nursing" OR "Rehabilitation Center" OR "Activities of Daily Living" OR "Animal Assisted Therapy" OR "Equine-Assisted Therapy" OR "Art Therapy" OR "Bibliotherapy" OR "Correction of Hearing Impairment" OR "Total Communication Methods" OR "Lipreading" OR "Manual Communication" OR "Dance Therapy" OR "Early Ambulation" OR "Exercise Therapy" OR "Endurance Training" OR "Continuous Passive Motion Therapy" OR "Muscle Stretching" OR "Plyometric Exercise" OR "Resistance Training" OR "Music Therapy" OR "Occupational Therapy" OR "Recreation Therapy" OR "Language Therapy" OR "Myofunctional Therapy" OR "Speech Therapy" OR "Alaryngeal Speech" OR "Voice Training" OR "Telerehabilitation" OR "Activity of Daily Living" OR "Plyometric Exercises" OR "Exercise" OR "exercise" OR "exercises" OR "physical activity") AND TI("elderly" OR "elder" OR "elders" OR geriatr* OR "Homes for the Aged" OR "Elderly Care" OR "Senior Centers" OR older person* OR old person* OR older patient* OR old patient* OR "older women" OR "old women" OR "older men" OR "old men" OR old adult* OR older adult* OR "Older individual" OR "Older individuals" OR "old people" OR "older people" OR "Oldest Old" OR "Nonagenarians" OR "Nonagenarian" OR "Octogenarians" OR "Octogenarian" OR "Centenarians" OR "Centenarian" OR "septuagenarian" OR "septuagenarians" OR "Aging" OR "aging" OR "ageing" OR "older population" OR "aging population" OR "aging population" OR geront* OR "old-aged" OR "old-age" OR "old aged" OR "old age") AND TI("accelerometers" OR "accelerometer" OR "accelerometry" OR acceleromet* OR "Telehealth" OR "web portals" OR "web portal" OR "web portal" OR "e-consult" OR econsult* OR telemed* OR "ehealth" OR "e-health" OR "mhealth" OR "m-health" OR "mobile health" OR "telehealth" OR "tele-health" OR "tele health" OR "**electronic communication"** OR "remote consultation" OR "remote health care" OR "remote healthcare" OR "remote care" OR "remote monitoring" OR "teleconsultation" OR teleconsult* OR **mobile* OR "webbased" OR "web-based" OR "Electronic Mail" OR "Electronic Mail" OR "e-mail" OR email*** OR "Mobile Application" OR "mobile apps" OR "mobile app" OR webapp* OR "SMS" OR "Cell Phones" OR "Smartphone" OR "Text Messaging" OR "Mobile Phone" OR "Cell Phone" OR "Smartphones" OR iphon* OR "text messag" OR "texting" OR **"mobile"** OR "cellular phone" OR "cellular phones" OR "smart phone" OR "telemedicine" OR "tele-care" OR "telecare" OR "tele-monitoring" OR "telemonitoring" OR **"website" OR "websites"** OR "personal digital assistant" OR "computer-assisted instruction" OR "ipad" OR ipad* OR "telenursing" OR telenurs* OR "virtual community" **OR "webpage" OR "webpages"** OR "web application" OR "web applications" OR "short message service" OR **"Internet" OR "internet" OR "online" OR "digital" OR digital*** OR "Reminder System" OR "Reminder Systems" OR

"Reminder System" OR "Reminder Device" OR "Reminder Devices" OR "reminder messages" OR "reminder message" OR **"web"** OR "Virtual Reality" OR "Virtual Reality" OR "smart technology" OR "smart technol" OR "wearable technology" OR "wearable technologies" OR "telerehabilitation" OR "computer-assisted therapy" OR "computer-assisted therapy" OR "computer assisted therapy" OR "online therapy" OR "Computer Mediated Communication" OR "Computer Mediated Communications" OR **"remote" OR "on-line" OR "on line"** OR "wearable devices" OR "wearable device" OR "healthsensor" OR "healthsensors" OR "health sensor" OR "health sensors" OR "Robotics" OR "robotics" OR robot* OR "exergame" OR "exergames" OR exergam* OR "Video Game" OR "app-based" OR "wearable app" OR "wearable apps" OR "wearables" OR "Nintendo" OR "Wii" OR "gaming console" OR "gaming consoles"))

OR (TI("Rehabilitation" OR rehab* OR "Rehabilitation Nursing" OR "Rehabilitation Center" OR "Activities of Daily Living" OR "Animal Assisted Therapy" OR "Equine-Assisted Therapy" OR "Art Therapy" OR "Bibliotherapy" OR "Correction of Hearing Impairment" OR "Total Communication Methods" OR "Lipreading" OR "Manual Communication" OR "Dance Therapy" OR "Early Ambulation" OR "Exercise Therapy" OR "Endurance Training" OR "Continuous Passive Motion Therapy" OR "Muscle Stretching" OR "Plyometric Exercise" OR "Resistance Training" OR "Music Therapy" OR "Occupational Therapy" OR "Recreation Therapy" OR "Language Therapy" OR "Myofunctional Therapy" OR "Speech Therapy" OR "Alaryngeal Speech" OR "Voice Training" OR "Telerehabilitation" OR "Activity of Daily Living" OR "Plyometric Exercises" OR "Exercise" OR "exercise" OR "exercises" OR "physical activity") AND SU("elderly" OR "elder" OR "elders" OR geriatr* OR "Homes for the Aged" OR "Elderly Care" OR "Senior Centers" OR older person* OR old person* OR older patient* OR old patient* OR "older women" OR "old women" OR "older men" OR "old men" OR old adult* OR older adult* OR "Older individual" OR "Older individuals" OR "old people" OR "older people" OR "Oldest Old" OR "Nonagenarians" OR "Nonagenarian" OR "Octogenarians" OR "Octogenarian" OR "Centenarians" OR "Centenarian" OR "septuagenarian" OR "septuagenarians" OR "Aging" OR "aging" OR "ageing" OR "older population" OR "aging population" OR "aging population" OR geront* OR "old-aged" OR "old-age" OR "old aged" OR "old age") AND TX("accelerometers" OR "accelerometer" OR "accelerometry" OR acceleromet* OR "Telehealth" OR "web portals" OR "web portal" OR "web portal" OR "e-consult" OR econsult* OR telemed* OR "ehealth" OR "e-health" OR "mhealth" OR "m-health" OR "mobile health" OR "telehealth" OR "tele-health" OR "tele health" OR "**electronic communication"** OR "remote consultation" OR "remote health care" OR "remote healthcare" OR "remote care" OR "remote monitoring" OR "teleconsultation" OR teleconsult* OR **mobile* OR "webbased" OR "web-based" OR "Electronic Mail" OR "Electronic Mail" OR "e-mail" OR email*** OR "Mobile Application" OR "mobile apps" OR "mobile app" OR webapp* OR "SMS" OR "Cell Phones" OR "Smartphone" OR "Text Messaging" OR "Mobile Phone" OR "Cell Phone" OR "Smartphones" OR iphon* OR "text messag" OR "texting" OR **"mobile"** OR "cellular phone" OR "cellular phones" OR "smart phone" OR "telemedicine" OR "tele-care" OR "telecare" OR "tele-monitoring" OR "telemonitoring" OR **"website" OR "websites"** OR "personal digital assistant" OR "computer-assisted instruction" OR "ipad" OR ipad* OR "telenursing" OR telenurs* OR "virtual community" **OR "webpage" OR "webpages"** OR "web application" OR "web

applications" OR "short message service" OR **"Internet" OR "internet" OR "online" OR "digital" OR digital*** OR "Reminder System" OR "Reminder Systems" OR "Reminder System" OR "Reminder Device" OR "Reminder Devices" OR "reminder messages" OR "reminder message" OR **"web"** OR "Virtual Reality" OR "Virtual Reality" OR "smart technology" OR "smart technol" OR "wearable technology" OR "wearable technologies" OR "telerehabilitation" OR "computer-assisted therapy" OR "computer-assisted therapy" OR "computer assisted therapy" OR "online therapy" OR "Computer Mediated Communication" OR "Computer Mediated Communications" OR **"remote" OR "on-line" OR "on line"** OR "wearable devices" OR "wearable device" OR "healthsensor" OR "healthsensors" OR "health sensor" OR "health sensors" OR "Robotics" OR "robotics" OR robot* OR "exergame" OR "exergames" OR exergam* OR "Video Game" OR "app-based" OR "wearable app" OR "wearable apps" OR "wearables" OR "Nintendo" OR "Wii" OR "gaming console" OR "gaming consoles"))

OR (TX("Rehabilitation" OR rehab* OR "Rehabilitation Nursing" OR "Rehabilitation Center" OR "Activities of Daily Living" OR "Animal Assisted Therapy" OR "Equine-Assisted Therapy" OR "Art Therapy" OR "Bibliotherapy" OR "Correction of Hearing Impairment" OR "Total Communication Methods" OR "Lipreading" OR "Manual Communication" OR "Dance Therapy" OR "Early Ambulation" OR "Exercise Therapy" OR "Endurance Training" OR "Continuous Passive Motion Therapy" OR "Muscle Stretching" OR "Plyometric Exercise" OR "Resistance Training" OR "Music Therapy" OR "Occupational Therapy" OR "Recreation Therapy" OR "Language Therapy" OR "Myofunctional Therapy" OR "Speech Therapy" OR "Alaryngeal Speech" OR "Voice Training" OR "Telerehabilitation" OR "Activity of Daily Living" OR "Plyometric Exercises" OR "Exercise" OR "exercise" OR "exercises" OR "physical activity") AND SU("elderly" OR "elder" OR "elders" OR geriatr* OR "Homes for the Aged" OR "Elderly Care" OR "Senior Centers" OR older person* OR old person* OR older patient* OR old patient* OR "older women" OR "old women" OR "older men" OR "old men" OR old adult* OR older adult* OR "Older individual" OR "Older individuals" OR "old people" OR "older people" OR "Oldest Old" OR "Nonagenarians" OR "Nonagenarian" OR "Octogenarians" OR "Octogenarian" OR "Centenarians" OR "Centenarian" OR "septuagenarian" OR "septuagenarians" OR "Aging" OR "aging" OR "ageing" OR "older population" OR "aging population" OR "aging population" OR geront* OR "old-aged" OR "old-age" OR "old aged" OR "old age") AND TI("accelerometers" OR "accelerometer" OR "accelerometry" OR acceleromet* OR "Telehealth" OR "web portals" OR "web portal" OR "web portal" OR "e-consult" OR econsult* OR telemed* OR "ehealth" OR "e-health" OR "mhealth" OR "m-health" OR "mobile health" OR "telehealth" OR "tele-health" OR "tele health" OR "**electronic communication"** OR "remote consultation" OR "remote health care" OR "remote healthcare" OR "remote care" OR "remote monitoring" OR "teleconsultation" OR teleconsult* OR **mobile* OR "webbased" OR "web-based" OR "Electronic Mail" OR "Electronic Mail" OR "e-mail" OR email*** OR "Mobile Application" OR "mobile apps" OR "mobile app" OR webapp* OR "SMS" OR "Cell Phones" OR "Smartphone" OR "Text Messaging" OR "Mobile Phone" OR "Cell Phone" OR "Smartphones" OR iphon* OR "text messag" OR "texting" OR **"mobile"** OR "cellular phone" OR "cellular phones" OR "smart phone" OR "telemedicine" OR "tele-care" OR "telecare" OR "tele-monitoring" OR "telemonitoring" OR **"website" OR "websites"** OR "personal digital assistant" OR

"computer-assisted instruction" OR "ipad" OR ipad* OR "telenursing" OR telenurs* OR "virtual community" **OR "webpage" OR "webpages"** OR "web application" OR "web applications" OR "short message service" OR **"Internet" OR "internet" OR "online" OR "digital" OR digital*** OR "Reminder System" OR "Reminder Systems" OR "Reminder System" OR "Reminder Device" OR "Reminder Devices" OR "reminder messages" OR "reminder message" OR **"web"** OR "Virtual Reality" OR "Virtual Reality" OR "smart technology" OR "smart technol" OR "wearable technology" OR "wearable technologies" OR "telerehabilitation" OR "computer-assisted therapy" OR "computer-assisted therapy" OR "computer assisted therapy" OR "online therapy" OR "Computer Mediated Communication" OR "Computer Mediated Communications" OR **"remote" OR "on-line" OR "on line"** OR "wearable devices" OR "wearable device" OR "healthsensor" OR "healthsensors" OR "health sensor" OR "health sensors" OR "Robotics" OR "robotics" OR robot* OR "exergame" OR "exergames" OR exergam* OR "Video Game" OR "app-based" OR "wearable app" OR "wearable apps" OR "wearables" OR "Nintendo" OR "Wii" OR "gaming console" OR "gaming consoles")))
AND (TX("usability" OR "Meaningful Use" OR "User experience" OR "User experiences" OR "usefulness") OR DE "Human Factors Engineering")
